# Improved adaptive EWMA control chart for process location with applications in groundwater physicochemical parameters and glass manufacturing industry

**DOI:** 10.1371/journal.pone.0272584

**Published:** 2022-08-22

**Authors:** Muhammad Arslan, Syed Masroor Anwar, Showkat Ahmad Lone, Zahid Rasheed, Majid Khan, Saddam Akbar Abbasi

**Affiliations:** 1 Department of Mathematics Air University Pakistan, Islamabad, Pakistan; 2 Department of Statistics, University of Azad Jammu and Kashmir, Muzaffarabad, Pakistan; 3 Department of Basic Sciences, College of Science and Theoretical Studies, Saudi Electronic University, Jeddah Branch, Riyadh, Kingdom of Saudi Arabia; 4 Department of Mathematics, Women University of Azad Jammu and Kashmir, Muzaffarabad, Pakistan; 5 Department of Mathematics and Statistics, Riphah International University, Islamabad, Pakistan; 6 Department of Mathematics, Statistics and Physics, Qatar University, Doha, Qatar; Universidad Rey Juan Carlos, SPAIN

## Abstract

The adaptive exponentially weighted moving average (AEWMA) control charts are the advanced form of classical memory control charts used for efficiently monitoring small-to-large shifts in the process parameters (location and/or dispersion). These AEWMA control charts estimate the unknown shifts using exponentially weighted moving average (EWMA) or cumulative sum (CUSUM) control charts statistics. The hybrid EWMA (HEWMA) control chart is preferred over classical memory control charts to detect early shifts in process parameters. So, this study presents a new auxiliary information-based (AIB) AEWMA (IAEWMA_AIB_) control chart for process location that estimates the unknown location shift using HEWMA statistic. The objective is to develop an unbiased location shift estimator using HEWMA statistic and then adaptively update the smoothing constant. The shift estimation using HEWMA statistic instead of EWMA or CUSUM statistics boosts the performance of the proposed IAEWMA_AIB_ control chart. The Monte Carlo simulation technique is used to get the numerical results. Famous performance evaluation measures like average run length, extra quadratic loss, relative average run length, and performance comparison index are used to evaluate the performance of the proposed chart with existing counterparts. The comparison reveals the superiority of the proposed control chart. Finally, two real-life applications from the glass manufacturing industry and physicochemical parameters of groundwater are considered to show the proposed control chart’s implementation procedure and dominance.

## 1: Introduction

Ever since Shewhart [1] presented a conventional Shewhart control chart, it has become a typical practice to utilize these control charts for monitoring the assignable cause variations (shifts) in different manufacturing/production processes [2]. The Shewhart control charts are referred to as memoryless control charts; these charts do not carry previous information. The key limitation of these control charts is that they generally monitor large shift sizes in the process parameters (location and/or dispersion). On the other hand, the memory control chart, like the exponentially weighted moving average (EWMA) control chart proposed by Roberts [3], is incredibly beneficial for monitoring small to moderate process shifts.

Recently, different processes demand balanced and sufficient safety against the unknown shifts of large and small sizes in industries. The adaptive control charts are often suggested to catch both Shewhart and EWMA control charts’ desirable properties and simultaneously disclose large and small shifts. In this regard, Capizzi and Masarotto [4] introduced an adaptive EWMA (AEWMA) control chart based on score (Huber’s and Tukey’s bi-square) functions, which are very effective for monitoring small and large shifts simultaneously. Likewise, Zaman *et al*. [5] extended the existing structure of the AEWMA control chart with CUSUM accumulation error using score functions that signal out shift more precisely than the AEWMA control chart. Similarly, Haq *et al*. [6] introduced the AEWMA control chart by utilizing the unbiased estimator of the process location shift through EWMA statistic, and then the EWMA control chart’s smoothing constant is adaptively updated. Also, Haq [7] introduced the auxiliary information-based (AIB) estimator AEWMA (AEWMA_AIB_) control chart for the process location by estimating the unknown process location shift.

Mixing or combining the features of various control charts improves the conventional control charts’ performance. For instance, Haq [8] presented a hybrid EWMA (HEWMA) control chart by combining two EWMA statistics. The one EWMA plotting statistic serves as an input for the other EWMA control chart. The HEWMA control chart is more efficient than the classical EWMA control chart in monitoring small-to-moderate shifts in the process location. Later on, different researchers examined the HEWMA control chart with some additional features. For example, Azam *et al*. [9] provided a HEWMA control chart for process location under the assumption of repetitive sampling. Similarly, Aslam *et al*. [10] presented the HEWMA chart for Com-Poisson distribution. Likewise, Noor-ul-Amin *et al*. [11] demonstrated an AIB HEWMA (HEWMA_AIB_) chart under the assumption of phase-II process location monitoring. Similarly, Aslam *et al*. [12] suggested a HEWMA-CUSUM chart by combining the HEWMA and CUSUM statistics. Recently, Anwar *et al*. [13] introduced AIB double homogeneously weighted moving average (AIB DHWMA) control chart to monitor process location. Also, Rasheed *et al*. [14] and Rasheed *et al*. [15] suggested mixed memory and nonparametric triple EWMA control charts for improved process location monitoring.

Over the last few years, auxiliary information has been highly esteemed for process monitoring. Many authors have studied various features of the AIB EWMA type control charts. For example, Abbas *et al*. [16] designed an AIB EWMA control chart. Similarly, Adegoke *et al*. [17] introduced an AIB EWMA control chart using different sampling schemes. Also, Haq [18] proposed nonparametric EWMA_AIB_ sign control chart. Later, Abbasi and Haq [19] recommended an auxiliary-based optimal and adaptive CUSUM control chart for process location. Likewise, Anwar *et al*. [20] and Anwar *et al*. [21] introduced modified-EWMA_AIB_ and AIB mixed control charts, respectively, for improved process location monitoring. Recently, Haq *et al*. [22] suggested AIB adaptive Crosier CUSUM (ACC_AIB_) under fixed and variable sampling intervals and AIB adaptive EWMA (AE_AIB_) control charts for the monitoring of process mean. For more details about adaptive EWMA, hybrid EWMA, and AIB memory control charts, see [23–27] and reference therein.

As mentioned before, Zaman *et al*. [5] suggested the enhanced AEWMA control chart that estimates the unknown location shift using CUSUM statistic instead of EWMA statistic. Inspired by the innovation in AEWMA structure of [5], we intend to present an improved AEWMA_AIB_ (symbolized as IAEWMA_AIB_) control chart for enhanced monitoring of the process location. The proposed IAEWMA_AIB_ control chart uses the HEWMA statistic and an unbiased shift estimator to estimate the process location shift. Then, depending on the magnitude of the shift, it determines an appropriate value for the smoothing constant. The shift estimation with the HEWMA statistic rather than the CUSUM or EWMA statistic boosts the detection ability of the proposed IAEWMA_AIB_ control chart. To evaluate the performance of the proposed IAEWMA_AIB_ control chart against other control charts, performance evaluation measures such as average run length (ARL), extra quadratic loss (EQL), performance comparison index (PCI), and relative ARL (RARL) measures are considered. Besides, the Monte Carlo simulation method is used to calculate the performance evaluation measures. Existing control chart such as AIB CUSUM (CUSUM_AIB_), EWMA_AIB_, HEWMA, AEWMA_AIB_, AIB improved adaptive Crosier CUSUM (IACCUSUM_AIB_), ACC_AIB_, AE_AIB_, and mixed HWMA-CUSUM (MHC) control charts are considered for comparison. Moreover, to demonstrate the utility of the proposed IAEWMA_AIB_ control chart for practical importance, two real-life applications are also provided.

The remainder of the article is arranged as follows: The existing memory control charts are described in Section 2. Similarly, Section 3 illustrates the construction of the proposed IAEWMA_AIB_ control chart, and also special cases of the proposed IAEWMA_AIB_ control chart. Additionally, the performance evaluation measures and parameter choices are available in Section 4. Similarly, Section 5 provides the performance comparison of the proposed IAEWMA_AIB_ control chart against CUSUM_AIB_, EWMA_AIB_, HEWMA, AEWMA_AIB_, and IACCUSUM_AIB_ control charts. Additionally, the real-life applications of the proposed IAEWMA_AIB_ control chart are provided in section 6. The last section presents the overall summary, conclusions, and recommendations.

## 2: Existing methods

This section provides insight into the variable of interest and AIB estimator in Subsection 2.1. Likewise, the methodologies of the classical EWMA and HEWMA control charts are presented in Subsections 2.2 and 2.3, respectively.

### 2.1: Variable of interest and transformation

Let $Y∼N(\mu_{Y}+\delta \sigma_{Y},\sigma_{Y}^{2})$ be the variable of interest from a normal distribution. Let ${\bar{Y}}_{t}=\sum_{i=1}^{n}Y_{it}/n$ be the sample mean and $S_{Yt}=\sqrt{\sum_{i=1}^{n}{\left(Y_{it}-{\bar{Y}}_{t}\right)}^{2}/\left(n-1\right)}$ be the sample standard deviation of *Y*. So, for the in-control (IC) situation, *δ* = 0, ${\bar{Y}}_{t}∼N(\mu_{Y},\sigma_{Y}^{2}/n)$ and $S_{Yt}^{2}∼\sigma_{Y}^{2}/\left(n-1\right)(\chi_{n-1}^{2})$. Let *X* be an auxiliary variable of *Y*. The variables *X* and *Y* follow a bivariate normal distribution (BND) (i.e., (*Y*, *X*)~*N*(*μ*_*Y*_, *μ*_*X*_, *σ*_*Y*_, *σ*_*X*_, *ρ*), where *μ*_*X*_ represents the mean and *σ*_*X*_ represents the standard deviation of *X*. Also, the *ρ* is the correlation coefficient corresponding to *X* and *Y*. Let (*Y*_*it*_, *X*_*it*_), *i* = 1,2,…*n* be a random sample of size *n* at time *t*, for *t*≥1. Following Haq and Khoo [28] and Haq [29], the AIB difference estimator for monitoring the process location is

$${\hat{\mu }}_{Y}=Y+b_{YX}(\mu_{X}-X),$$ (1)

where $b_{YX}=\rho (\frac{\sigma_{Y}}{\sigma_{X}})$, Here, ${\hat{\mu }}_{Y}$ follows a normal distribution with mean *μ*_*Y*_ and the variance $\sigma_{Y}^{2}(1-\rho^{2})$, that is, ${\hat{\mu }}_{Y}∼N(\mu_{Y},\sigma_{Y}^{2}(1-\rho^{2}))$. Following Haq [29], we assume $T_{t}=\left({\hat{\mu }}_{Y}-\mu_{Y}\right)/\sqrt{\sigma_{Y}^{2}(1-\rho^{2})}$, then *T*_*t*_~*N*(δ*, 1), where $\delta^{*}=\delta /\sqrt{\left(1-\rho^{2}\right)}$ and known as the process mean shift. When a process is IC then *T*_*t*_~*N*(0,1), that is, δ* = 0. Let *δ* = |*μ*_*Y*_−*μ*_*Y*,1_|/*σ*_*Y*_ be the standardized shift in the *σ*_*Y*_ units, where *μ*_*Y*,1_ is OOC process mean. The process is IC for *δ* = 0, else, out-of-control (OOC).

### 2.2: Classical EWMA control chart

Roberts [3] suggested the classic EWMA control chart for monitoring small-to-moderate shifts in the process location. Based on {*Y*_*t*_} the plotting statistic of the classical EWMA control chart is designed as follows:

$$E_{t}=\lambda_{1}Y_{t}+(1-\lambda_{1})E_{t-1},$$ (2)

where, *E*_0_ = 0 and *λ*_1_ (0<*λ*_1_≤1) is the smoothing constant. If *μ*_*Y*_ and *σ*_*Y*_ are known, then the upper control limit (*UCL*) and lower control limit (*LCL*) are defined as:

$$({UCL}_{t},{LCL}_{t})=\mu_{0}\pm L\sigma \sqrt{\frac{\lambda_{1}}{2-\lambda_{1}}(1-{\left(1-\lambda_{1}\right)}^{2t})},$$ (3)

for large *t*, the control limits can be written as:

$$(UCL,LCL)=\mu_{0}\pm L\sigma \sqrt{\frac{\lambda_{1}}{2-\lambda_{1}}},$$ (4)

where *L* denotes the control chart coefficient for a predefined false alarm rate. The EWMA statistic *Z*_*t*_ is plotted along *UCL*_*t*_ and *LCL*_*t*_, the process is considered to be OOC when *Z*_*t*_<*LCL*_*t*_ or *Z*_*t*_>*UCL*_*t*_; otherwise, it is IC.

### 2.3: HEWMA control chart

Haq [8] introduced the HEWMA control chart for process location. This control chart is constructed by using one EWMA statistic as an input for another EWMA statistic given as:

$$\begin{array}{c} E_{t}=(1-\lambda_{1})E_{t-1}+\lambda_{1}Y_{t},\;0<\lambda_{1}\leq 1\\ {HE}_{t}=(1-\lambda_{2}){HE}_{t-1}+\lambda_{2}E_{t},\;0<\lambda_{2}\leq 1 \end{array}\},$$ (5)

Here, the *HE*_*t*_ is called HEWMA statistic at time *t*. The starting values of *HE*_*t*_ and *E*_0_ are assumed to be *μ*_0_, that is *HE*_0_ = *E*_0_ = *μ*_0_. Based on the statistic *HE*_*t*_ the *LCL* and *UCL* for HEWMA control chart are given by

$$({UCL}_{t},{LCL}_{t})=\mu_{0}\pm L\sigma \sqrt{{\left(\frac{\lambda_{1}\lambda_{2}}{\lambda_{1}-\lambda_{2}}\right)}^{2}[\sum_{i=1}^{2}\frac{{\left(1-\lambda_{i}\right)}^{2}\{1-{\left(1-\lambda_{i}\right)}^{2t}\}}{1-{\left(1-\lambda_{i}\right)}^{2}}-\frac{2(1-\lambda_{1})(1-\lambda_{2})\{1-{\left(1-\lambda_{1}\right)}^{t}{\left(1-\lambda_{2}\right)}^{t}\}}{1-(1-\lambda_{1})(1-\lambda_{2})}]},$$ (6)

The control limits of the HEWMA control chart when *t* is very large.

$$(UCL,LCL)=\mu_{0}\pm L\sigma \sqrt{{\left(\frac{\lambda_{1}\lambda_{2}}{\lambda_{1}-\lambda_{2}}\right)}^{2}[\sum_{i=1}^{2}{\left(1-\lambda_{i}\right)}^{2}-\frac{2(1-\lambda_{1})(1-\lambda_{2})}{1-(1-\lambda_{1})(1-\lambda_{2})}]},$$ (7)

where the *L* is the control chart coefficient, and it is decided in such a way that the IC ARL (ARL_0_) of the HEWMA control chart is determined at a pre-specified desired level. The process is considered to be OOC when *HE*_*t*_<*LCL*_*t*_ or *HE*_*t*_>*UCL*_*t*_; otherwise, it is IC.

## 3: Proposed methods

This section contains the methodology of the proposed IAEWMA_AIB_ control chart for monitoring the process location. Subsection 3.1 covers the design structure of the proposed IAEWMA_AIB_ control chart. Besides, the special cases of the IAEWMA_AIB_ control chart are given in Subsection 3.2.

### 3.1: Proposed IAEWMA_AIB_ control chart

Here, we extend the work of Haq [29] and propose IAEWMA_AIB_ control chart for the improved monitoring of process location. The proposed IAEWMA_AIB_ control chart first estimates the shift estimator δ* using HEWMA statistic and based on the estimated value of the shift estimator, appropriate values of smoothing parameters are selected to construct the proposed chart. Let $\left\{{\hat{\delta }}_{t}^{*}\right\}$ is a sequence based on {*T*_*t*_} for IC process and $\left\{{\hat{\delta }}_{t}^{**}\right\}$ is another sequance defined on $\left\{{\hat{\delta }}_{t}^{*}\right\}$, then the estimator of ${\hat{\delta }}_{t}^{**}$ using the HEWMA statistic is defined as:

$$\begin{array}{c} {\hat{\delta }}_{t}^{*}=\lambda_{1}T_{t}+(1-\lambda_{1}){\hat{\delta }}_{t-1}^{*},\;0<\lambda_{1}\leq 1\\ {\hat{\delta }}_{t}^{**}=\lambda_{2}{\hat{\delta }}_{t}^{*}+(1-\lambda_{2}){\hat{\delta }}_{t-1}^{**},\;0<\lambda_{2}\leq 1 \end{array}\},$$ (8)

where *λ*_1_ and *λ*_2_ are the smoothing constants for the HEWMA statistic. The initial values of ${\hat{\delta }}_{t}^{*}$ and ${\hat{\delta }}_{t}^{**}$ are zero, i.e., ${\hat{\delta }}_{0}^{*}={\hat{\delta }}_{0}^{**}=0$. The shift estimator ${\hat{\delta }}_{t}^{**}$ is unbiased for the IC process and biased for the OOC process. The unbiased estimator of ${\hat{\delta }}_{t}^{**}$, even if the process is either IC or OOC, it is given by

$${\hat{\delta }}_{t}^{\left(2\right)}=\frac{{\hat{\delta }}_{t}^{**}}{c},$$ (9)

where $c=\frac{\lambda_{1}-\lambda_{2}+\lambda_{2}{\left(1-\lambda_{1}\right)}^{t}-\lambda_{1}{\left(1-\lambda_{2}\right)}^{t+1}-\lambda_{1}{\lambda_{2}(1-\lambda_{1})}^{t}}{\lambda_{1}-\lambda_{2}}$. Following Haq [29], we assumed ${\tilde{\delta }}_{t}^{\left(2\right)}=|{\hat{\delta }}_{t}^{\left(2\right)}|$. Based on a sequence of IID random variable {*T*_*t*_}, the plotting-statistic of the proposed IAEWMA_AIB_ control chart is defined as:

$${AHE}_{t}=g({\tilde{\delta }}_{t}^{\left(2\right)})T_{t}+\left\{1-g({\tilde{\delta }}_{t}^{\left(2\right)})\right\}{AHE}_{t-1},$$ (10)

where *AHE*_0_ = 0 and $g({\tilde{\delta }}_{t}^{\left(2\right)})\in (0,1]$ is defined by

$$g({\tilde{\delta }}_{t}^{\left(2\right)})=\left\{ \begin{array}{l} 0.015\;if\;0.00<{\tilde{\delta }}_{t}^{\left(2\right)}\leq 0.25\\ 0.10\;if\;0.25<{\tilde{\delta }}_{t}^{\left(2\right)}\leq 0.75\\ 0.20\;if\;0.75<{\tilde{\delta }}_{t}^{\left(2\right)}\leq 1.00\\ 0.25\;if\;1.00<{\tilde{\delta }}_{t}^{\left(2\right)}\leq 1.50\\ 0.50\;if\;1.50<{\tilde{\delta }}_{t}^{\left(2\right)}\leq 2.50\\ 0.80\;if\;2.50<{\tilde{\delta }}_{t}^{\left(2\right)}\leq 3.50\\ 1.00\;if\;3.50<{\tilde{\delta }}_{t}^{\left(2\right)} \end{array}\right..$$ (11)

Here $g({\tilde{\delta }}_{t}^{\left(2\right)})$ is a smoothing multiplier of the proposed IAEWMA_AIB_ control chart, which is a function of the shift estimator ${\tilde{\delta }}_{t}^{\left(2\right)}$. The control chart chooses smaller values of $g({\tilde{\delta }}_{t}^{\left(2\right)})$ when ${\tilde{\delta }}_{t}^{\left(2\right)}$ is small and larger values of $g({\tilde{\delta }}_{t}^{\left(2\right)})$ when ${\tilde{\delta }}_{t}^{\left(2\right)}$ is large. The proposed IAEWMA_AIB_ control chart detects an OOC signal when |*AHE*_*t*_| exceeds than threshold says $h_{IAEWMA_{AIB}}$.

### 3.2: Special cases of IAEWMA_AIB_ control chart

The AEWMA and AEWMA_AIB_ control charts are special cases of the proposed IAEWMA_AIB_ control chart by considering special values of parameters. The special cases with their proofs are provided here.

**Case 1:** When *ρ* = 0 and *λ*_2_ = 1, the proposed IAEWMA_AIB_ control chart tends to the AEWMA control chart.

**Proof:** When *ρ* = 0, then the difference ${\hat{\mu }}_{Y}$ estimator reduces to

$${\hat{\mu }}_{t}^{\left(1\right)}={\bar{Y}}_{t}.$$ (12)

The transformed *T* can be written as: $T^{\left(1\right)}=\left({\hat{\mu }}_{t}^{\left(1\right)}-\mu_{Y}\right)/\sqrt{\sigma_{Y}^{2}}$.

Now substitute the resulting ${\hat{\mu }}_{t}^{\left(1\right)}$ in ${\hat{\delta }}_{t}^{*}$ to obtain

$${\hat{\delta }}_{t}^{*(1)}=\lambda_{1}T^{\left(1\right)}+(1-\lambda_{1}){\hat{\delta }}_{t-1}^{*(1)}.$$ (13)

Also, put *λ*_2_ = 1 in Eq (8), then the ${\hat{\delta }}_{t}^{**}$ reduced as follows:

$${\hat{\delta }}_{t}^{**(1)}={\hat{\delta }}_{t}^{*(1)},$$ (14)

which is the estimated shift estimator of the existing AEWMA control chart. Based on Eq (10), the IAEWMA_AIB_ chart can be expressed as:

$$AHE_{t}^{*(1)}=g({\tilde{\delta }}_{t}^{*(2)})T^{\left(1\right)}+\left\{1-g({\tilde{\delta }}_{t}^{*(2)})\right\}AHE_{t-1}^{*(1)},$$ (15)

where $g({\tilde{\delta }}_{t}^{*(2)})$ can be obtained in similar ways as described in Eq (11). The $AHE_{t}^{*(1)}$ statistic and shift estimator ${\hat{\delta }}_{t}^{**(1)}$ are similar to the plotting statistic and shift estimator of AEWMA proposed by Haq *et al*. [6] except for their notations. Hence, the statistic of the proposed IAEWMA_AIB_ control chart becomes the statistic of AEWMA when *ρ* = 0 and *λ*_2_ = 1.

**Case 2:** When *λ*_2_ = 1, the IAEWMA_AIB_ control chart tends to the AEWMA_AIB_ control chart.

**Proof:** When *λ*_2_ = 1, the shift estimator ${\hat{\delta }}_{t}^{**}$ in Eq (8) reduced as follows:

$${\hat{\delta }}_{t}^{**(2)}={\hat{\delta }}_{t}^{*}.$$ (16)

Then the plotting statistic of the proposed control chart can be written as

$$AHE_{t}^{*(2)}=g({\tilde{\delta }}_{t}^{**(2)})T_{t}+\left\{1-{\tilde{\delta }}_{t}^{**(2)}\right\}AHE_{t-1}^{**(2)}$$ (17)

where $g({\tilde{\delta }}_{t}^{**(2)})$ can be obtained in similar ways as described in Eq (11). The shift estimator ${\hat{\delta }}_{t}^{**(2)}$ and plotting statistic $AHE_{t}^{*(2)}$ in Eqs (16) and (17) are similar to the shift estimator and plotting statistic of AEWMA_AIB_ control chart, which indicates that the proposed IAEWMA_AIB_ control chart reduced to the AEWMA_AIB_ control chart for *λ*_2_ = 1.

## 4: Performance evaluation measures

This section introduces the performance evaluation measures to analyze the control charts’ performance. The Monte Carlo simulation detail is given in Subsection 4.1. Likewise, the description of the ARL is enlisted in Subsection 4.2. Similarly, the overall performance evaluation measures are defined in Subsection 4.3. The choices of parameters of the proposed IAEWMA_AIB_ control chart is given in Subsection 4.4.

### 4.1: Monte Carlo simulation

The Monte Carlo simulation procedure is regarded as a computational technique for obtaining numerical results for evaluating the performance of the proposed IAEWMA_AIB_ control chart. Monte Carlo simulation with 10^5^ iterations is conducted for each displacement of *δ* using R software to obtain the ARL and standard deviation of RL (SDRL) of the proposed control chart. The sample values of (*Y*_*t*_, *X*_*t*_), for *t*>1 are generated from BND. The shift reflected in the process location is considered here *δ* = 0.10, 0.20, 0.25, 0.30, 0.40, 0.50, 0.75, 1.00, 1.50, 2.00, 2.5, 3, 3.5, 4, 5, 6. The simulation algorithm of the proposed IAEWMA_AIB_ control chart is described as follows:

- Generate a random sample from BND.
- Calculate the ${\hat{\mu }}_{Y}$ estimator from Eq (1) and *T*_*t*_.
- Calculate the ${\hat{\delta }}_{t}^{*}$ statistic from Eq (8) using *T*_*t*_ estimator.
- Use the ${\hat{\delta }}_{t}^{*}$ statistic as an input in ${\hat{\delta }}_{t}^{**}$.
- Estimate ${\hat{\delta }}_{t}^{\left(2\right)}$ from Eq (9) and estimate $g({\tilde{\delta }}_{t}^{\left(2\right)})$ using Eq (11).
- Estimate the IAEWMA_AIB_ statistic *AHE*_*t*_ from Eq (10).
- Compute threshold $h_{IAEWMA_{AIB}}$ for desired in-control ARL denoted as ARL_0_.
- Plot the |*AHE*_*t*_| statistic against the threshold $h_{IAEWMA_{AIB}}$.
- If $|{AHE}_{t}|>h_{IAEWMA_{AIB}}$, record sequence order known as run-length (RL).
- Record RL_s_ after repeating steps (i)-(ix) 10^5^ times.
- Determine the average of 10^5^ RL, which is ARL_0_.
- For out-of-control ARL values, considered $\left(Y_{it},X_{it})∼N(\mu_{Y+\delta \sigma_{Y}},\mu_{X},\;\sigma_{Y},\sigma_{X},\rho \right)$ and repeat from steps (ii)-(x).

### 4.2: The ARL measure

The average run length (ARL) is commonly used to evaluate a control chart’s performance at a shift. The ARL is listed as IC ARL (ARL_0_) and OOC ARL (ARL_1_). If a process is functioning in an in-control state, the ARL_0_ is chosen to be sufficiently large to eliminate the effect of the false alarm rate. On the other hand, the ARL_1_ should be small enough to detect a shift quickly. A control chart is preferred over the other competing charts if it should have a smaller ARL_1_ value at predefined ARL_0_ [30].

### 4.3: Overall performance measures

The EQL, RARL, and PCI performance evaluation measures are used to evaluate a control chart’s overall effectiveness. The EQL is the weighted average ARL over the domain of shifts with *δ*^2^ as a weight [31]. It is defined as

$$EQL={\left(\delta_{max}-\delta_{min}\right)}^{-1}\int_{\delta_{min}}^{\delta_{max}}\delta^{2}\;ARL(\delta )d\delta ,$$

where *ARL*(*δ*) is the ARL at specific *δ*; *δ*_*min*_ and *δ*_*max*_ are the smallest and largest shift values of the domain, respectively. The lower the EQL value signifies, the better the performance of the control chart [20].

The RARL, like EQL, is also used to assess the efficiency of a control chart. It can be defined as follows:

$$RARL={\left(\delta_{max}-\delta_{min}\right)}^{-1}\int_{\delta_{min}}^{\delta_{max}}\frac{ARL(\delta )}{ARL^{*}(\delta )}d\delta .$$

The *ARL*(*δ*) is ARL of the competing control chart. The ARL*(*δ*) is ARL of benchmark control chart at a *δ*. A control chart is decided as a benchmark control chart for a smaller ARL at specific *δ* [21]. The RARL value of the benchmark control chart is assumed to be one. If the competing control chart has RARL>1, the benchmark control chart is more efficient than the competing one.

The PCI corresponds to the EQL ratio of the specific chart to theEQL of the benchmark chart. Here EQL* represents the EQL of the benchmark chart, whereas the *EQL* represents the EQL of the competing control chart. According to the [32], the PCI is presented as:

$$PCI=\frac{EQL}{EQL^{*}}.$$

The PCI of the benchmark control chart should be one. If the PCI>1, the benchmark chart is superior to the competing control chart [20].

### 4.4: Effect of parameters choices

The parameters (*λ*_1_, *λ*_2_, and *ρ*) has their effects on the performance of the proposed IAEWMA_AIB_ control chart. Various combinations of these parameters are chosen; hence, corresponding ARL and SDRL are computed. The parameter *λ*_1_ is set as 0.10, 0.20, and 0.50, whereas the parameter *λ*_2_ is set as 0.10, 0.20,0.50, 0.75,0.90 to obtain ARL_0_ = 500. Similarly, the values of *ρ* are assumed as 0.25, 0.50, 0.75, and 0.95. Tables 1–3 present the numerical results of the proposed IAEWMA_AIB_ chart.

**Table 1:** The run-length profile of proposed IAEWMA_AIB_ control chart under various choices of *λ*_2_ when *λ*_1_ is small and ARL_0_ = 500.

*ρ*		*λ*_1_ = 0.10, *λ*_2_ = 0.10, $h_{IAEWMA_{AIB}}$ = 0.5172																
	*δ*	0.00	0.10	0.20	0.25	0.30	0.40	0.50	0.75	1.00	1.50	2.00	2.50	3.00	3.50	4.00	5.00	6.00
0.25	ARL	498.44	215.28	71.84	44.73	30.00	16.66	11.19	5.15	2.92	1.54	1.18	1.05	1.02	1.00	1.00	1.00	1.00
	SDRL	826.51	348.65	114.63	68.90	46.55	24.95	15.15	6.61	3.39	1.23	0.53	0.24	0.12	0.06	0.03	0.00	0.00
0.50	ARL	502.08	156.51	45.92	28.64	19.42	10.76	7.26	3.23	1.96	1.22	1.05	1.01	1.00	1.00	1.00	1.00	1.00
	SDRL	820.70	251.81	71.17	43.87	29.04	14.91	9.73	3.90	1.90	0.62	0.25	0.11	0.04	0.01	0.00	0.00	0.00
0.75	ARL	501.48	60.61	15.13	9.81	6.71	3.93	2.63	1.42	1.13	1.01	1.00	1.00	1.00	1.00	1.00	1.00	1.00
	SDRL	817.83	94.58	21.50	13.28	9.00	4.73	2.96	1.00	0.44	0.09	0.00	0.00	0.00	0.00	0.00	0.00	0.00
0.95	ARL	504.29	3.21	1.22	1.08	1.02	1.00	1.00	1.00	1.00	1.00	1.00	1.00	1.00	1.00	1.00	1.00	1.00
	SDRL	814.30	3.83	0.62	0.31	0.16	0.03	0.00	0.00	0.00	0.00	0.00	0.00	0.00	0.00	0.00	0.00	0.00
		*λ*_1_ = 0.10, *λ*_2_ = 0.20, $h_{IAEWMA_{AIB}}=0.5704$																
	*δ*	0.00	0.10	0.20	0.25	0.30	0.40	0.50	0.75	1.00	1.50	2.00	2.50	3.00	3.50	4.00	5.00	6.00
0.25	ARL	498.60	233.43	81.49	51.27	35.70	19.34	12.13	5.27	3.05	1.54	1.19	1.05	1.01	1.00	1.00	1.00	1.00
	SDRL	775.38	369.70	126.79	77.02	54.65	27.72	16.32	6.87	3.68	1.20	0.54	0.25	0.12	0.06	0.03	0.00	0.00
0.50	ARL	501.33	171.85	51.95	33.90	22.83	11.96	7.67	3.39	2.04	1.24	1.06	1.01	1.00	1.00	1.00	1.00	1.00
	SDRL	781.87	273.18	79.59	50.34	32.53	16.39	10.28	4.15	2.04	0.67	0.27	0.11	0.05	0.00	0.00	0.00	0.00
0.75	ARL	502.27	71.49	16.27	10.75	7.34	4.07	2.70	1.49	1.12	1.01	1.00	1.00	1.00	1.00	1.00	1.00	1.00
	SDRL	777.55	110.52	23.26	14.85	9.62	5.16	3.05	1.11	0.42	0.10	0.01	0.00	0.00	0.00	0.00	0.00	0.00
0.95	ARL	498.60	3.29	1.20	1.06	1.02	1.00	1.00	1.00	1.00	1.00	1.00	1.00	1.00	1.00	1.00	1.00	1.00
	SDRL	775.38	3.98	0.56	0.28	0.16	0.02	0.00	0.00	0.00	0.00	0.00	0.00	0.00	0.00	0.00	0.00	0.00
		*λ*_1_ = 0.10, *λ*_2_ = 0.50, $h_{IAEWMA_{AIB}}=0.6339$																
	*δ*	0.00	0.10	0.20	0.25	0.30	0.40	0.50	0.75	1.00	1.50	2.00	2.50	3.00	3.50	4.00	5.00	6.00
0.25	ARL	497.33	219.83	81.35	53.49	39.56	22.13	13.51	5.74	3.17	1.60	1.20	1.07	1.02	1.01	1.00	1.00	1.00
	SDRL	770.60	330.92	119.40	78.32	55.62	30.30	17.80	7.40	3.71	1.23	0.53	0.28	0.15	0.07	0.02	0.00	0.00
0.50	ARL	498.68	173.06	54.91	35.54	24.45	13.81	8.52	3.48	2.13	1.27	1.07	1.01	1.00	1.00	1.00	1.00	1.00
	SDRL	753.22	252.83	79.59	51.06	35.13	18.98	11.24	4.12	2.02	0.64	0.27	0.12	0.04	0.00	0.01	0.00	0.00
0.75	ARL	498.58	75.56	18.96	11.55	7.92	4.24	2.76	1.47	1.16	1.01	1.00	1.00	1.00	1.00	1.00	1.00	1.00
	SDRL	745.77	108.82	26.30	15.37	10.52	5.23	3.02	0.99	0.47	0.11	0.02	0.00	0.00	0.00	0.00	0.00	0.00
0.95	ARL	497.31	3.47	1.24	1.08	1.03	1.00	1.00	1.00	1.00	1.00	1.00	1.00	1.00	1.00	1.00	1.00	1.00
	SDRL	740.13	4.15	0.62	0.32	0.18	0.05	0.00	0.00	0.00	0.00	0.00	0.00	0.00	0.00	0.00	0.00	0.00
		*λ*_1_ = 0.10, *λ*_2_ = 0.75, $h_{IAEWMA_{AIB}}=0.689$																
	*δ*	0.00	0.10	0.20	0.25	0.30	0.40	0.50	0.75	1.00	1.50	2.00	2.50	3.00	3.50	4.00	5.00	6.00
0.25	ARL	498.38	227.28	85.61	58.19	40.79	23.46	14.10	5.85	3.23	1.70	1.23	1.08	1.03	1.01	1.00	1.00	1.00
	SDRL	730.60	329.88	119.86	79.89	55.59	31.80	19.34	7.55	3.63	1.30	0.57	0.30	0.17	0.09	0.04	0.00	0.00
0.50	ARL	500.98	177.83	59.33	38.16	26.96	14.67	8.85	3.54	2.16	1.30	1.09	1.02	1.00	1.00	1.00	1.00	1.00
	SDRL	739.35	255.23	82.89	52.30	36.89	19.87	11.76	4.16	1.99	0.69	0.31	0.13	0.05	0.00	0.00	0.00	0.00
0.75	ARL	500.53	76.74	20.47	12.23	8.06	4.31	2.90	1.52	1.18	1.01	1.00	1.00	1.00	1.00	1.00	1.00	1.00
	SDRL	740.81	106.25	27.09	16.51	11.04	5.22	3.09	1.04	0.46	0.12	0.01	0.00	0.00	0.00	0.00	0.00	0.00
0.95	ARL	502.98	3.43	1.28	1.09	1.03	1.00	1.00	1.00	1.00	1.00	1.00	1.00	1.00	1.00	1.00	1.00	1.00
	SDRL	725.94	3.98	0.65	0.31	0.18	0.06	0.00	0.00	0.00	0.00	0.00	0.00	0.00	0.00	0.00	0.00	0.00

**Table 2:** The run-length profile of proposed IAEWMA_AIB_ control chart under various choices of *λ*_2_ when *λ*_1_ is moderate and ARL_0_ = 500.

*ρ*		*λ*_1_ = 0.20, *λ*_2_ = 0.10, $h_{IAEWMA_{AIB}}=0.55061$																
	*δ*	0.00	0.10	0.20	0.25	0.30	0.40	0.50	0.75	1.00	1.50	2.00	2.50	3.00	3.50	4.00	5.00	6.00
0.25	ARL	503.56	243.80	86.62	57.91	39.30	22.93	14.38	6.24	3.61	1.82	1.28	1.10	1.03	1.01	1.00	1.00	1.00
	SDRL	540.72	253.98	86.64	54.19	38.99	22.72	13.39	6.07	3.53	1.43	0.64	0.35	0.18	0.10	0.05	0.00	0.00
0.50	ARL	502.54	181.47	58.51	36.65	25.80	13.97	9.27	4.07	2.44	1.38	1.10	1.02	1.00	1.00	1.00	1.00	1.00
	SDRL	535.21	186.41	58.49	36.54	25.14	13.43	9.29	4.01	2.28	0.81	0.34	0.14	0.06	0.02	0.00	0.00	0.00
0.75	ARL	502.11	77.36	19.27	12.77	8.29	4.88	3.18	1.64	1.21	1.02	1.00	1.00	1.00	1.00	1.00	1.00	1.00
	SDRL	533.20	81.81	19.69	11.53	8.27	4.50	3.14	1.19	0.54	0.15	0.01	0.00	0.00	0.00	0.00	0.00	0.00
0.95	ARL	503.42	3.86	1.34	1.12	1.05	1.00	1.00	1.00	1.00	1.00	1.00	1.00	1.00	1.00	1.00	1.00	1.00
	SDRL	530.20	4.05	0.73	0.37	0.23	0.05	0.00	0.00	0.00	0.00	0.00	0.00	0.00	0.00	0.00	0.00	0.00
		*λ*_1_ = 0.20, *λ*_2_ = 0.20, $h_{IAEWMA_{AIB}}=0.5989394$																
	*δ*	0.00	0.10	0.20	0.25	0.30	0.40	0.50	0.75	1.00	1.50	2.00	2.50	3.00	3.50	4.00	5.00	6.00
0.25	ARL	503.26	250.67	94.68	63.96	46.29	25.31	16.17	7.02	3.85	1.89	1.33	1.13	1.04	1.01	1.00	1.00	1.00
	SDRL	531.50	263.69	95.64	63.47	45.27	24.69	15.94	7.07	3.02	1.46	0.69	0.38	0.21	0.11	0.06	0.00	0.00
0.50	ARL	502.63	193.35	63.19	41.68	29.58	16.28	10.01	4.46	2.50	1.42	1.14	1.03	1.01	1.00	1.00	1.00	1.00
	SDRL	529.12	196.11	63.24	40.03	28.88	16.71	9.28	4.36	2.38	0.81	0.39	0.18	0.07	0.01	0.00	0.00	0.00
0.75	ARL	501.18	87.18	21.93	14.10	9.73	5.23	3.34	1.73	1.25	1.02	1.00	1.00	1.00	1.00	1.00	1.00	1.00
	SDRL	525.04	90.30	21.37	14.07	8.92	5.06	3.23	1.25	0.58	0.15	0.04	0.00	0.00	0.00	0.00	0.00	0.00
0.95	ARL	498.20	4.20	1.40	1.15	1.05	1.01	1.00	1.00	1.00	1.00	1.00	1.00	1.00	1.00	1.00	1.00	1.00
	SDRL	527.40	4.42	0.80	0.41	0.22	0.07	0.00	0.00	0.00	0.00	0.00	0.00	0.00	0.00	0.00	0.00	0.00
		*λ*_1_ = 0.20, *λ*_2_ = 0.50, $h_{IAEWMA_{AIB}}=0.7795878$																
	*δ*	0.00	0.10	0.20	0.25	0.30	0.40	0.50	0.75	1.00	1.50	2.00	2.50	3.00	3.50	4.00	5.00	6.00
0.25	ARL	497.86	284.87	119.98	83.64	58.85	32.59	20.63	8.22	4.55	2.10	1.41	1.15	1.05	1.01	1.00	1.00	1.00
	SDRL	514.75	295.73	119.46	82.68	57.39	31.29	18.24	8.06	4.54	1.70	0.81	0.43	0.24	0.12	0.05	0.00	0.00
0.50	ARL	498.49	231.03	83.71	56.16	38.17	20.07	12.12	5.24	2.92	1.55	1.15	1.04	1.01	1.00	1.00	1.00	1.00
	SDRL	517.14	235.60	82.85	55.31	37.41	19.68	11.14	4.38	2.65	1.00	0.43	0.20	0.09	0.03	0.00	0.00	0.00
0.75	ARL	498.37	108.99	28.81	17.50	11.83	6.27	3.98	1.87	1.30	1.03	1.00	1.00	1.00	1.00	1.00	1.00	1.00
	SDRL	514.44	109.62	27.81	16.77	10.82	6.15	3.92	1.40	0.65	0.17	0.05	0.00	0.00	0.00	0.00	0.00	0.00
0.95	ARL	500.60	4.99	1.47	1.19	1.06	1.00	1.00	1.00	1.00	1.00	1.00	1.00	1.00	1.00	1.00	1.00	1.00
	SDRL	511.68	5.16	0.92	0.49	0.26	0.06	0.00	0.00	0.00	0.00	0.00	0.00	0.00	0.00	0.00	0.00	0.00
		*λ*_1_ = 0.20, *λ*_2_ = 0.75, $h_{IAEWMA_{AIB}}=0.9002$																
	*δ*	0.00	0.10	0.20	0.25	0.30	0.40	0.50	0.75	1.00	1.50	2.00	2.50	3.00	3.50	4.00	5.00	6.00
0.25	ARL	501.10	301.04	123.43	84.48	62.84	35.05	22.42	9.02	4.93	2.29	1.43	1.17	1.04	1.01	1.00	1.00	1.00
	SDRL	504.66	300.22	121.84	82.81	60.20	33.30	21.96	9.01	4.86	1.91	0.89	0.48	0.22	0.12	0.06	0.00	0.00
0.50	ARL	498.87	232.65	85.50	54.85	38.87	21.58	13.40	5.66	3.18	1.61	1.17	1.04	1.01	1.00	1.00	1.00	1.00
	SDRL	505.32	233.74	84.22	53.63	37.52	20.87	13.37	5.62	2.94	1.11	0.48	0.20	0.09	0.03	0.00	0.00	0.00
0.75	ARL	501.58	110.50	29.59	18.77	12.80	6.84	4.42	2.04	1.33	1.03	1.00	1.00	1.00	1.00	1.00	1.00	1.00
	SDRL	501.12	108.31	28.56	18.66	11.47	6.68	4.18	1.62	0.75	0.19	0.04	0.00	0.00	0.00	0.00	0.00	0.00
0.95	ARL	503.50	5.29	1.54	1.19	1.07	1.00	1.00	1.00	1.00	1.00	1.00	1.00	1.00	1.00	1.00	1.00	1.00
	SDRL	503.31	5.26	1.01	0.53	0.29	0.06	0.00	0.00	0.00	0.00	0.00	0.00	0.00	0.00	0.00	0.00	0.00

**Table 3:** The run-length profile of proposed IAEWMA_AIB_ control chart under various choices of *λ*_2_ when *λ*_1_ is large and ARL_0_ = 500.

*ρ*		*λ*_1_ = 0.50, *λ*_2_ = 0.20, $h_{IAEWMA_{AIB}}=0.7771575$																
	*δ*	0.00	0.10	0.20	0.25	0.30	0.40	0.50	0.75	1.00	1.50	2.00	2.50	3.00	3.50	4.00	5.00	6.00
0.25	ARL	502.04	293.58	124.87	86.48	61.54	34.35	21.43	8.80	4.82	2.26	1.49	1.17	1.05	1.01	1.00	1.00	1.00
	SDRL	514.23	297.67	125.24	86.26	60.50	33.52	20.46	8.04	4.63	1.84	0.94	0.51	0.24	0.12	0.06	0.00	0.00
0.50	ARL	505.48	177.83	59.33	38.16	26.96	14.67	8.85	3.54	2.16	1.30	1.09	1.02	1.00	1.00	1.00	1.00	1.00
	SDRL	511.33	181.23	59.89	37.80	26.89	14.57	8.26	3.16	1.99	0.69	0.31	0.13	0.05	0.00	0.00	0.00	0.00
0.75	ARL	504.48	113.25	28.53	18.48	12.26	6.66	4.21	2.07	1.35	1.03	1.00	1.00	1.00	1.00	1.00	1.00	1.00
	SDRL	509.01	114.26	28.20	17.62	12.08	6.55	3.88	1.59	0.76	0.19	0.03	0.00	0.00	0.00	0.00	0.00	0.00
0.95	ARL	505.32	5.29	1.55	1.21	1.06	1.00	1.00	1.00	1.00	1.00	1.00	1.00	1.00	1.00	1.00	1.00	1.00
	SDRL	510.47	4.96	1.01	0.56	0.27	0.07	0.00	0.00	0.00	0.00	0.00	0.00	0.00	0.00	0.00	0.00	0.00
		*λ*_1_ = 0.50, *λ*_2_ = 0.50, $h_{IAEWMA_{AIB}}=1.072987$																
	*δ*	0.00	0.10	0.20	0.25	0.30	0.40	0.50	0.75	1.00	1.50	2.00	2.50	3.00	3.50	4.00	5.00	6.00
0.25	ARL	500.54	318.67	147.50	101.83	74.01	43.16	27.36	12.39	6.69	2.80	1.69	1.26	1.10	1.03	1.01	1.00	1.00
	SDRL	503.61	317.10	142.67	100.91	73.34	42.67	25.93	11.32	5.86	2.36	1.14	0.58	0.32	0.19	0.10	0.02	0.00
0.50	ARL	499.76	259.65	102.03	68.91	49.47	27.77	17.53	7.48	4.05	1.84	1.28	1.07	1.02	1.00	1.00	1.00	1.00
	SDRL	500.34	258.98	101.77	67.28	47.26	25.65	16.10	6.65	3.55	1.32	0.61	0.27	0.12	0.05	0.01	0.00	0.00
0.75	ARL	500.09	129.89	36.82	24.07	16.79	9.04	5.74	2.49	1.50	1.06	1.00	1.00	1.00	1.00	1.00	1.00	1.00
	SDRL	501.30	128.00	34.96	22.38	15.04	7.93	5.04	2.01	0.90	0.25	0.05	0.01	0.00	0.00	0.00	0.00	0.00
0.95	ARL	498.45	7.22	1.80	1.31	1.12	1.01	1.00	1.00	1.00	1.00	1.00	1.00	1.00	1.00	1.00	1.00	1.00
	SDRL	500.83	6.50	1.25	0.66	0.36	0.11	0.02	0.00	0.00	0.00	0.00	0.00	0.00	0.00	0.00	0.00	0.00
		*λ*_1_ = 0.50, *λ*_2_ = 0.75, $h_{IAEWMA_{AIB}}=1.40871$																
	*δ*	0.00	0.10	0.20	0.25	0.30	0.40	0.50	0.75	1.00	1.50	2.00	2.50	3.00	3.50	4.00	5.00	6.00
0.25	ARL	503.19	398.92	236.18	175.69	133.78	78.47	48.90	18.76	9.55	3.70	2.17	1.54	1.25	1.10	1.03	1.00	1.00
	SDRL	499.54	399.97	236.13	175.59	133.48	78.45	47.78	18.18	8.57	2.77	1.33	0.77	0.49	0.31	0.18	0.04	0.00
0.50	ARL	500.36	351.12	172.43	119.41	88.95	48.87	29.94	11.28	5.57	2.36	1.54	1.20	1.06	1.01	1.00	1.00	1.00
	SDRL	500.05	352.87	171.25	118.80	86.48	47.35	28.15	10.26	4.69	1.53	0.76	0.44	0.24	0.11	0.05	0.00	0.00
0.75	ARL	503.56	218.52	68.08	42.56	28.23	13.82	8.00	3.21	1.94	1.17	1.01	1.00	1.00	1.00	1.00	1.00	1.00
	SDRL	498.86	220.40	68.06	41.33	26.68	12.65	7.15	2.32	1.08	0.40	0.11	0.03	0.00	0.00	0.00	0.00	0.00
0.95	ARL	498.80	10.62	2.27	1.63	1.29	1.05	1.00	1.00	1.00	1.00	1.00	1.00	1.00	1.00	1.00	1.00	1.00
	SDRL	499.44	9.67	1.39	0.85	0.53	0.22	0.06	0.00	0.00	0.00	0.00	0.00	0.00	0.00	0.00	0.00	0.00
		*λ*_1_ = 0.50, *λ*_2_ = 0.90, $h_{IAEWMA_{AIB}}=1.607892$																
	*δ*	0.00	0.10	0.20	0.25	0.30	0.40	0.50	0.75	1.00	1.50	2.00	2.50	3.00	3.50	4.00	5.00	6.00
0.25	ARL	497.19	369.01	212.60	158.58	120.46	75.42	48.10	20.03	10.57	4.22	2.40	1.65	1.29	1.11	1.04	1.00	1.00
	SDRL	500.04	368.17	212.36	155.69	118.58	72.72	45.50	17.72	8.87	3.05	1.50	0.88	0.57	0.33	0.19	0.04	0.00
0.50	ARL	500.64	333.70	161.27	115.66	84.40	48.18	29.71	12.25	6.39	2.72	1.68	1.23	1.07	1.02	1.00	1.00	1.00
	SDRL	496.49	333.62	157.22	115.30	81.35	45.22	26.92	10.27	4.97	1.76	0.90	0.49	0.26	0.13	0.04	0.00	0.00
0.75	ARL	498.62	203.42	67.70	42.61	29.12	15.19	9.12	3.71	2.14	1.20	1.02	1.00	1.00	1.00	1.00	1.00	1.00
	SDRL	496.60	200.57	65.02	40.45	26.28	12.99	7.55	2.62	1.32	0.45	0.14	0.02	0.00	0.00	0.00	0.00	0.00
0.95	ARL	497.67	11.39	2.57	1.76	1.35	1.06	1.00	1.00	1.00	1.00	1.00	1.00	1.00	1.00	1.00	1.00	1.00
	SDRL	498.03	9.47	1.71	1.00	0.62	0.23	0.05	0.00	0.00	0.00	0.00	0.00	0.00	0.00	0.00	0.00	0.00

## 5: Evaluation and performance comparison

This section includes comprehensive comparisons of the proposed IAEWMA_AIB_ control chart with CUSUM_AIB_ [33], EWMA_AIB_ [16], HEWMA [8], AEWMA_AIB_ [29], IACCUSUM_AIB_ [19], ACC_AIB_ and AE_AIB_ [22], MHC [34] control charts.

### 5.1: Proposed versus HEWMA control chart

**Fig 1:**
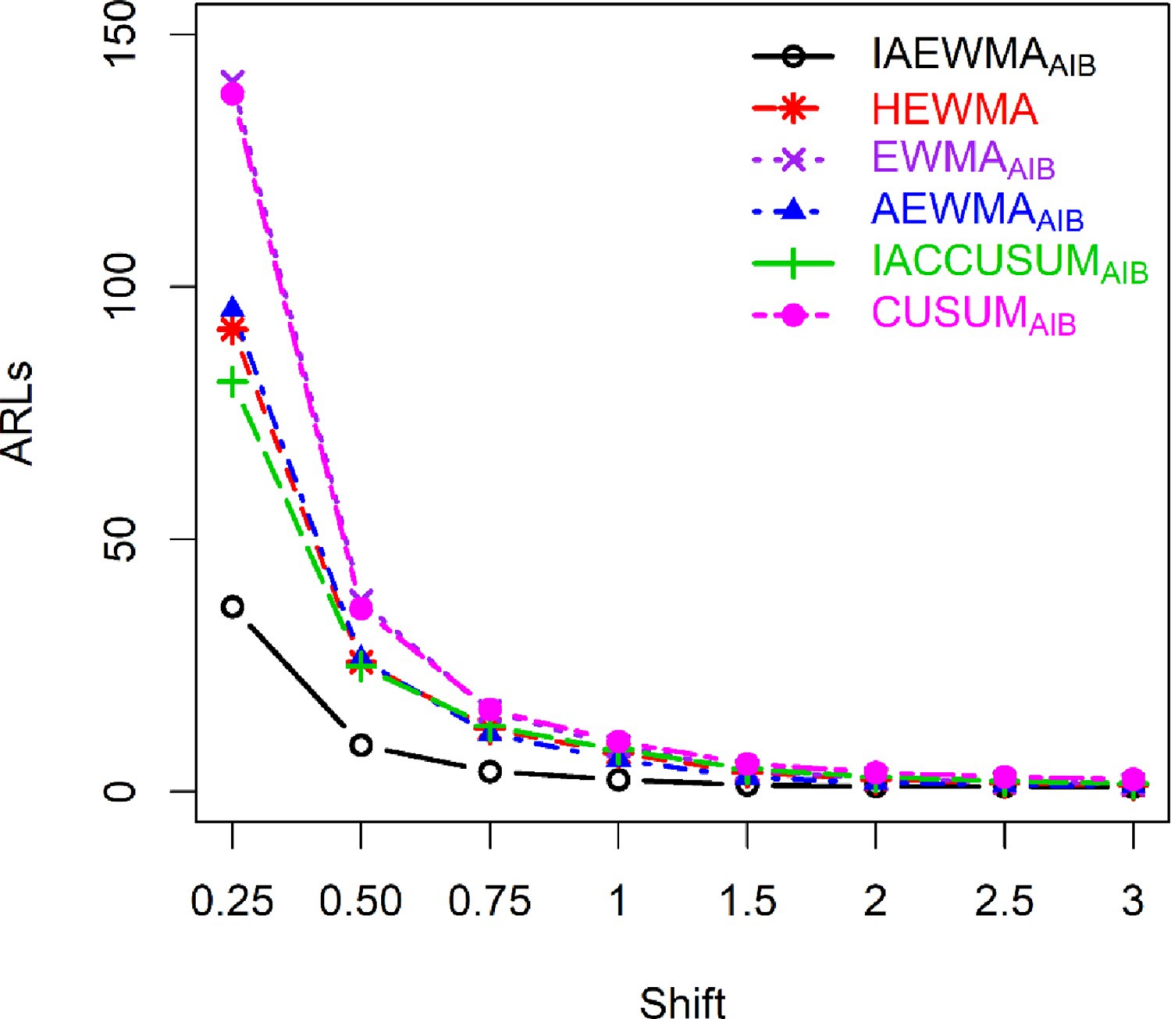
ARL profile of IAEWMA_AIB_, HEWMA, EWMA_AIB_, AEWMA_AIB_, IACCUSUM_AIB_, and CUSUM_AIB_ control charts at *ρ* = 0.25, (*λ*, *λ*_2_) = 0.20, *λ*_1_ = 0.10 when ARL_0_ = 500.

**Fig 2:**
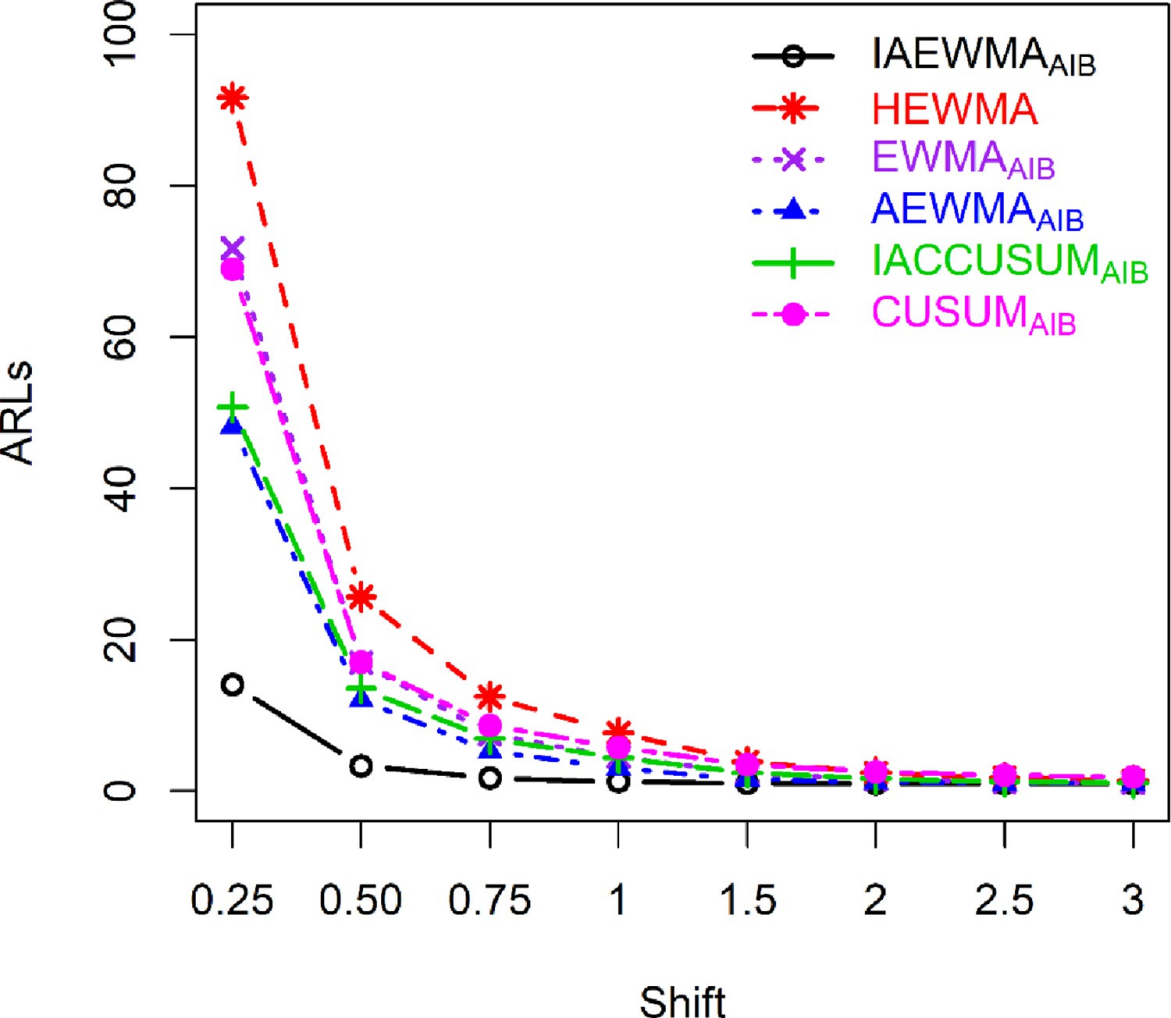
ARL profile of IAEWMA_AIB_, HEWMA, EWMA_AIB_, AEWMA_AIB_, IACCUSUM_AIB_, and CUSUM_AIB_ control charts at *ρ* = 0.75, (*λ*, *λ*_2_) = 0.20, *λ*_1_ = 0.10 when ARL_0_ = 500.

The proposed IAEWMA_AIB_ control chart provides better performance against the HEWMA control chart for different values of *ρ*. For example, at *λ*_1_ = 0.10, *λ*_2_ = 0.20, and *δ* = 0.25, 0.50, the proposed IAEWMA_AIB_ control chart (*ρ* = 0.50) has the ARL_1_ values 33.90, 7.67, while the HEWMA control chart has 91.64, 25.67 (see Tables 1 and 4). Furthermore, Figs 1 and 2 also demonstrate that the proposed IAEWMA_AIB_ control chart is superior to the HEWMA chart. In terms of overall effectiveness (see Table 5), the proposed IAEWMA_AIB_ control chart has smaller EQL, RARL, and PCI (i.e., 8.88, 1.00, 1.00) values against the HEWMA control chart EQL, RARL, and PCI (i.e., 12.24, 1.38, 1.92) values.

**Table 4:** The run length profile EWMA_AIB_, AEMWA_AIB_, CUSUM_AIB_, IACCUSUM_AIB_ and HEWMA control charts at ARL_0_ = 500.

	EWMA_AIB_			AEWMA_AIB_			CUSUM_AIB_			IACCUSUM_AIB_			HEWMA
*ρ*	0.25	0.50	0.75	0.25	0.50	0.75	0.25	0.50	0.75	0.25	0.50	0.75	
*δ*	*λ* = 0.2			*λ* = 0.2			*k* = 0.5, *h* = 5.071			*λ* = 0.2, $\delta_{min}^{+}=0.5$			*λ*_1_ = 0.1, *λ*_2_ = 0.2
0.00	502	500.34	498.13	499.56	500.24	499.96	500	500.88	499.06	500.19	500.02	501.14	501.15
0.25	140.59	117.88	71.76	95.57	78.63	48.21	138.23	114.52	68.99	81.16	71.23	50.69	91.64
0.50	37.82	29.66	16.99	26.3	21.01	12.05	36.36	28.9	17.07	24.86	20.71	13.60	25.67
0.75	15.95	12.72	7.52	11.43	9.06	5.28	16.32	13.38	8.65	12.97	10.75	6.90	12.47
1.00	9.06	7.27	4.54	6.35	5.09	3.02	9.99	8.41	5.76	8.37	6.89	4.41	7.70
1.50	4.31	3.59	2.35	2.90	2.37	1.56	5.58	4.83	3.51	4.53	3.73	2.43	3.87
2.00	2.72	2.30	1.58	1.78	1.52	1.16	3.91	3.43	2.58	2.95	2.46	1.65	2.45
2.50	1.96	1.68	1.21	1.34	1.2	1.04	3.04	2.7	2.11	2.14	1.80	1.26	1.75
3.00	1.53	1.33	1.06	1.14	1.07	1.01	2.52	2.27	1.87	1.67	1.42	1.08	1.37
3.50	1.27	1.15	1.01	1.05	1.02	1.00	2.19	2.01	1.61	1.38	1.20	1.01	1.17
4.00	1.12	1.05	1.00	1.02	1.00	1.00	1.98	1.84	1.32	1.19	1.08	1.00	1.07
5.00	1.02	1.00	1.00	1.00	1.00	1.00	1.66	1.42	1.02	1.03	1.00	1.00	1.01

**Table 5:** Overall performance measures of the existing and proposed IAEWMA_AIB_ control charts at different values of *ρ*.

Control	EQL	PCI	RARL	EQL	PCI	RARL	EQL	PCI	RARL
Chart		*ρ* = 0.25			*ρ* = 0.50			*ρ* = 0.75	
EWMA_AIB_	13.49	1.44	1.82	10.00	1.13	1.37	10.00	1.18	2.11
AEWMA_AIB_	10.89	1.16	1.35	10.15	1.14	1.44	9.18	1.08	1.61
CUSUM_AIB_	20.67	2.21	2.50	18.34	2.07	2.60	13.62	1.61	2.71
IACCUSUM_AIB_	13.72	1.47	1.77	12.09	1.36	1.82	9.96	1.17	1.96
HEWMA	12.24	1.31	1.58	12.24	1.38	1.92	12.24	1.44	3.04
IAEWMA_AIB_	9.35	1.00	1.00	8.88	1.00	1.00	8.48	1.00	1.00

### 5.2: Proposed versus EWMA_AIB_ control chart

In comparison to the EWMA_AIB_ control chart, the proposed IAEWMA_AIB_ control chart exhibits superior performance. As an illustration, at (*λ*, *λ*_1_, *λ*_2_) = 0.20, *ρ* = 0.5, and *δ* ∈ (0.25,0.5,0.75,1,1.5,2), the ARL_1_ (117.88, 29.66, 12.72, 7.27, 3.59, 2.30) values of the EWMA_AIB_ control chart are larger than the ARL_1_ (41.68, 10.01, 4.46, 2.50, 1.42, 1.14) values of the proposed IAEWMA_AIB_ control chart (see Tables 2 and 4). Likewise, Figs 1 and 2 displays the dominance of the proposed IAEWMA_AIB_ control chart over EWMA_AIB_ control chart. In addition, the EQL, RARL, and PCI values also show the dominance of the IAEWMA_AIB_ control chart against the EWMA_AIB_ control chart. For instance, at *ρ* = 0.25, the proposed IAEWMA_AIB_ control chart has EQL = 9.35, PCI = 1.00, and RARL = 1.00, while the EWMA_AIB_ control chart has EQL = 13.49, PCI = 1.44, RARL = 1.82 (see Table 5).

### 5.3: Proposed versus AEWMA_AIB_ control chart

The proposed IAEWMA_AIB_ control chart is superior to the AEWMA_AIB_ control chart. For example, if (*λ*, *λ*_1_, *λ*_2_) = 0.20, *ρ* = 0.50, and *δ* = 0.25, the ARL_1_ values of the AEWMA_AIB_ and IAEWMA_AIB_ are 78.63 and 41.68, respectively (see Tables 2 and 4 & Figs 1 and 2). Similarly, the EQL, PCI, and RARL values of the proposed IAEWMA_AIB_ control chart reveals the edge over the AEWMA_AIB_ control chart. As an illustration, at *ρ* = 0.50, the EQL, RARL, and PCI values of the AEWMA_AIB_ and IAEWMA_AIB_ control charts are presented as (10.15, 1.14, and 1.44), and (8.88, 1.00, and 1.00), respectively (see Table 5).

### 5.4: Proposed versus CUSUM_AIB_ control chart

The proposed IAEWMA_AIB_ control chart provides superior performance to the CUSUM_AIB_ control chart. For example, at *ρ* = 0.75, *δ* = 0.25,0.50,1.00, the proposed IAEWMA_AIB_ control chart (*λ*_1_ = 0.50, *λ*_2_ = 0.90) produces ARL_1_ = (42.61, 9.12, 2.14), whereas, the CUSUM_AIB_ control chart (*k* = 0.5) has ARL_1_ equal to (68.99, 17.07, 8.65) (see Tables 3 and 4). Furthermore, Figs 1 and 2 highlights the superiority of the proposed IAEWMA_AIB_ control chart over CUSUM_AIB_ chart. Similarly, at a specific range of shifts, the proposed IAEWMA_AIB_ control chart has smaller EQL, PCI, and RARL values than the CUSUM_AIB_ control chart. For example, at *ρ* = 0.75, the EQL, PCI, and RARL values are (8.48, 1.00, 1.00) and (13.62, 1.61, 2.71) for IAEWMA_AIB_ and CUSUM_AIB_ control charts, respectively (see Table 5).

### 5.5: Proposed versus IACCUSUM_AIB_ control chart

In comparison with IACCUSUM_AIB_, the proposed IAEWMA_AIB_ control chart detects an earlier shift. In more detail, if *δ* = 0.25, (*λ*, *λ*_1_, *λ*_2_) = 0.20, $\delta_{min}^{+}=0.5$, and *ρ* = 0.25, 0.50, 0.75, the ARL_1_ values of IACCUSUM_AIB_ and IAEWMA_AIB_ control chart are (81.16, 71.23, 50.69) and (63.96, 41.68, 14.10), respectively (see Tables 2 and 4). Figs 1 and 2 also demonstrate the edge of the proposed IAEWMA_AIB_ control chart against the IACCUSUM_AIB_ control chart. Similarly, at *ρ* = 0.75, the IACCUSUM_AIB_ control chart EQL, PCI, and RARL (i.e., 9.96, 1.17, and 1.96) values are larger than the EQL, PCI, and RARL (8.48, 1.00, and 1.00) values of the proposed IAEWMA_AIB_ control charts. (see Table 5).

### 5.6: Proposed versus ACC_AIB_ and AE_AIB_ control charts

The proposed IAEWMA_AIB_ control chart is compared with the ACC_AIB_ and AE_AIB_ control charts. The IAEWMA_AIB_ control chart is superior as compared to the ACC_AIB_ and AE_AIB_ control charts in terms of early shift detection. In more detail, if *δ* = 0.25, (*λ*, *λ*_1_, *λ*_2_) = 0.20, $\delta_{min}^{+}=0.5$, and *ρ* = 0.50, the ARL_1_ values of ACC_AIB_, AE_AIB_ and IAEWMA_AIB_ control chart are 80.77,93.72, and 41.68, respectively (see Tables 2 and 6). Similarly, the other entries in Tables 2 and 6 can be compared.

**Table 6:** The run length profile ACC_AIB_, AE_AIB_, and MHC control charts at ARL_0_ = 500.

	ACC_AIB_ (*δ*_*min*_ = 0.50)									
*δ*	0	0.25	0.5	0.75	1	1.5	2	3	4	5
*λ* = 0.20, *ρ* = 0.25	499.52	80.77	24.88	13.02	8.32	4.53	2.96	1.67	1.19	1.03
*λ* = 0.20, *ρ* = 0.50	499.54	71.15	20.82	10.75	6.88	3.73	2.45	1.43	1.08	1.00
*λ* = 0.20, *ρ* = 0.75	499.93	50.73	13.62	6.92	4.41	2.44	1.65	1.08	1.00	1.00
	AE_AIB_									
*δ*	0	0.25	0.5	0.75	1	1.5	2	3	4	5
*λ* = 0.20, *ρ* = 0.25	499.72	93.72	26.05	11.40	6.36	2.90	1.78	1.13	1.01	1.00
*λ* = 0.20, *ρ* = 0.50	500.40	76.23	20.77	9.10	5.14	2.41	1.51	1.05	1.00	1.00
*λ* = 0.20, *ρ* = 0.75	499.93	44.65	14.11	6.22	3.56	1.82	1.26	1.01	1.00	1.00
	MHC									
*δ*	0	0.1	0.15	0.2	0.25	0.5	0.75	1	1.5	2
*λ* = 0.20	501.00	62.00	41.00	32.00	25.00	13.00	9.00	7.00	5.00	4.00
*λ* = 0.50	501.00	222.00	128.00	79.00	52.00	16.00	9.00	6.00	4.00	3.00

### 5.7: Proposed versus MHC control chart

The MHC control chart is an improved control chart used for enhanced process monitoring. On comparing our proposed IAEWMA_AIB_ control chart with MHC chart, it is clear that the proposed IAEWMA_AIB_ control chart is superior to the MHC control chart for higher correlation coefficient values. For example, if *δ* = 0.10, 0.20, (*λ*, *λ*_1_, *λ*_2_) = 0.10, the ARL_1_ values of the MHC control chart are 62 and 32, whereas the ARL_1_ values of IAEWMA_AIB_ control chart (*ρ* = 0.95) are 3.21 and 1.22, respectively (see Tables 1 and 6). So, the proposed IAEWMA_AIB_ control chart is preferred for higher *ρ*, else the MHC control chart.

### 5.8: Main findings of the study

**Fig 3:**
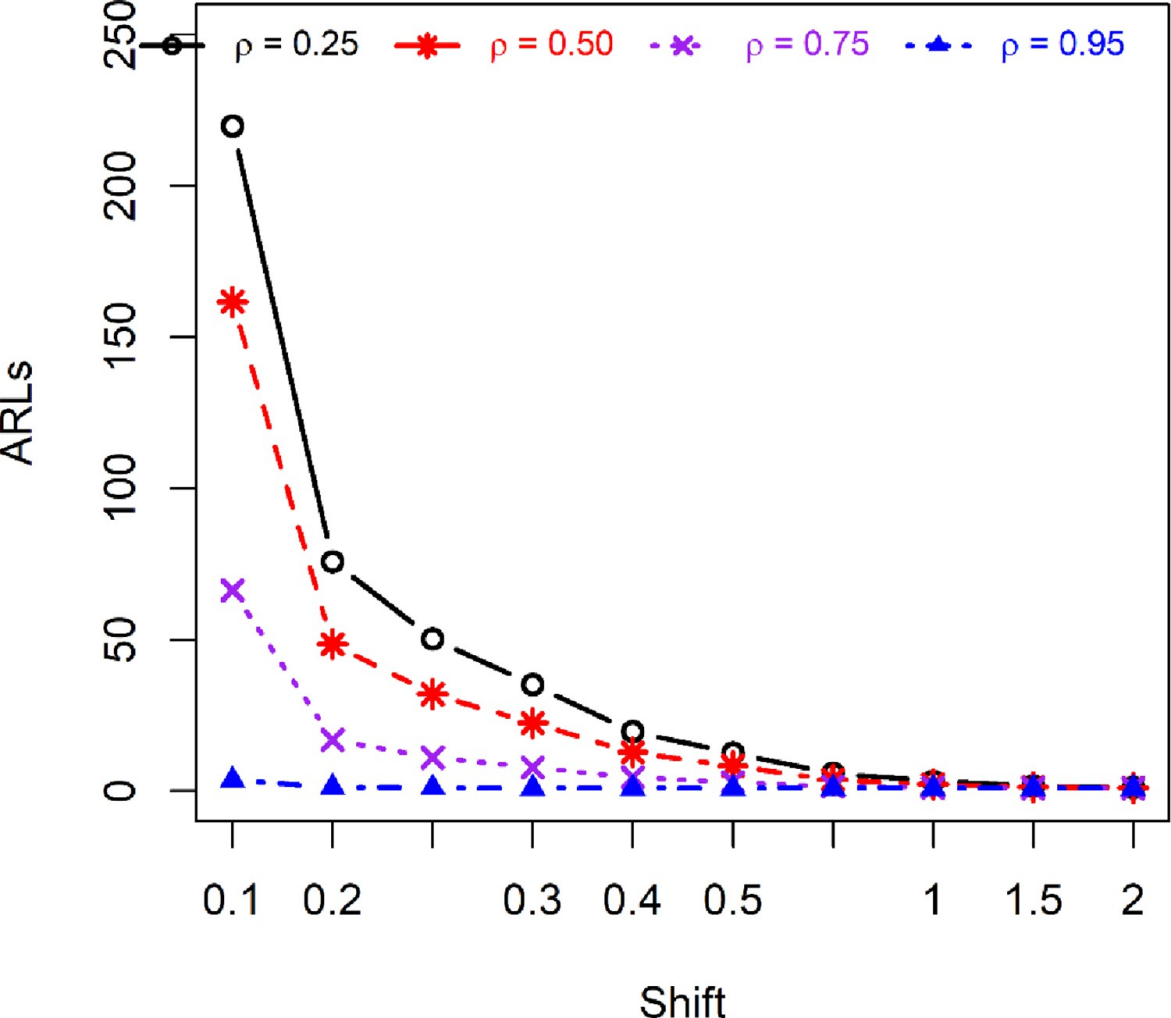
ARL profile of proposed IAEWMA_AIB_ control chart under different *ρ* for (*λ*_1_, *λ*_2_) = 0.10 at ARL_0_ = 500.

The following are the key findings of the proposed IAEWMA_AIB_ control chart:

- The use of HEWMA statistic with an adaptive scheme certainly boosts the detection ability of the proposed IAEWMA_AIB_ control chart.
- The auxiliary information improves the performance of the proposed IAEWMA_AIB_ control chart (see. Tables 1–3 and Fig 3)
- The ARL_1_ values of the proposed IAEWMA_AIB_ control chart are smaller than the competing control charts (i.e. CUSUM_AIB_, HEWMA, EWMA_AIB_, AEWMA_AIB_, and IACCUSUM_AIB_) (see Tables 1–3 versus Table 4).
- The overall performance evaluation measures show the dominance of the IAEWMA_AIB_ control chart against other control charts (see Subsections 5.1–5.5).
- The proposed IAEWMA_AIB_ control chart provides the best performance for larger values of *ρ* (see Fig 3).
- The ARL_1_ performance of the proposed IAEWMA_AIB_ control chart is increased for smaller *λ*_1_ and *λ*_2_ (see Tables 1–3).
- In short, the proposed IAEWMA_AIB_ control chart is superior for larger *ρ* and smaller *λ*_1_ and *λ*_2_ values.

## 6: Real-life applications

This section provides two real-life applications of the proposed IAEWMA_AIB_ control chart. More details are provided in the following subsections.

### 6.1: Application 1

**Fig 4:**
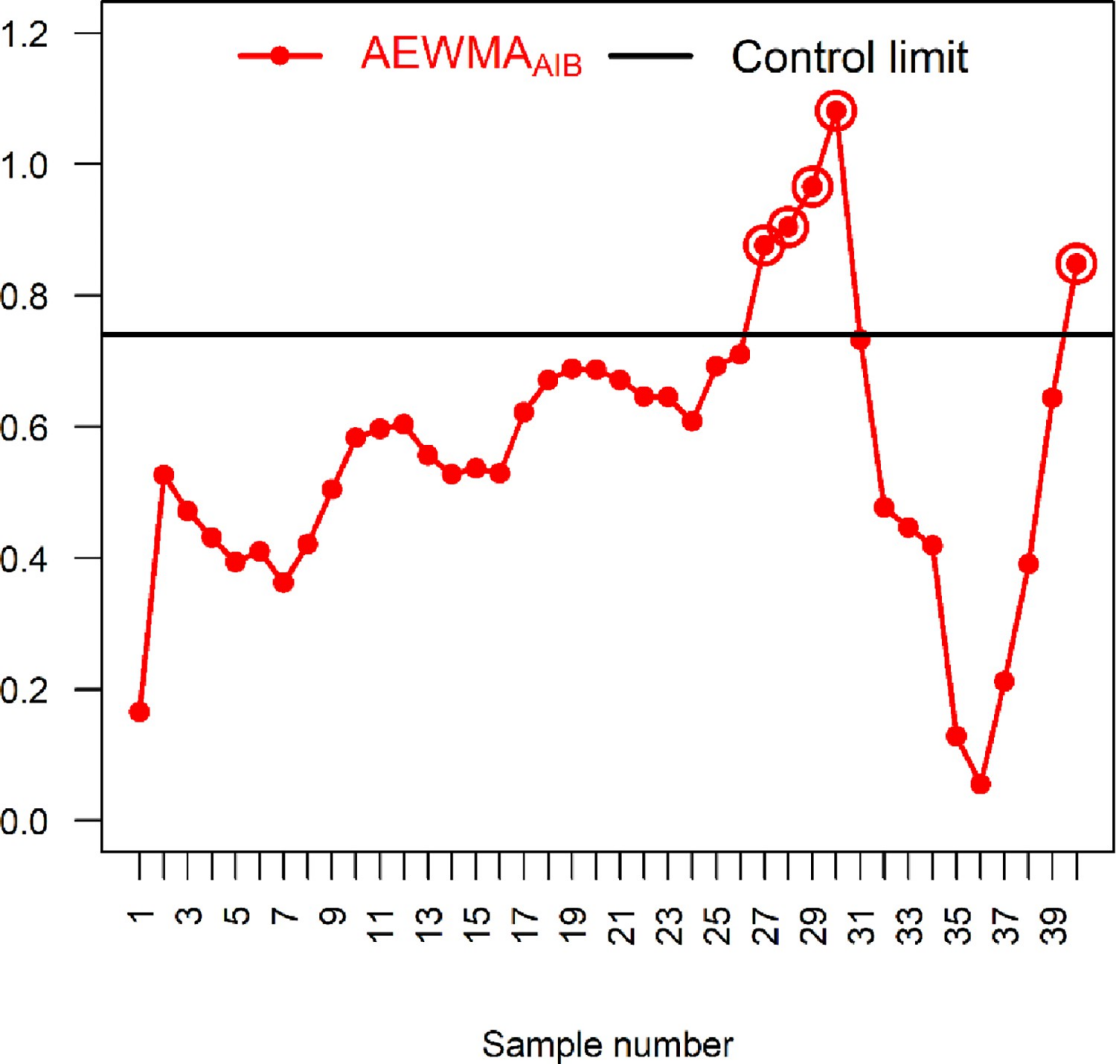
AEWMA_AIB_ chart with real-life data of glass manufacturing industry using *ρ* = 0.905, *λ* = 0.1, $h_{AEWMA_{AIB}}=0.7402$ and ARL_0_ = 500.

**Fig 5:**
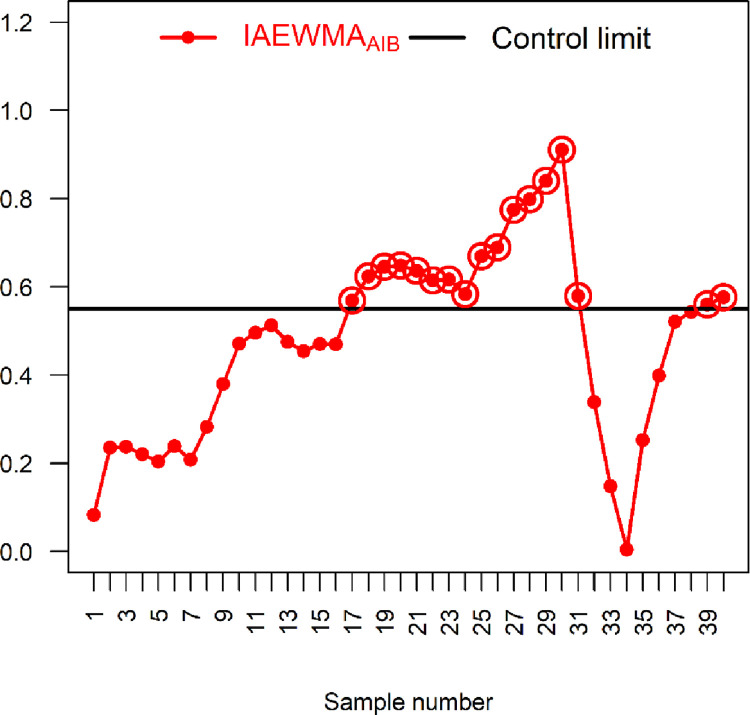
Proposed IAEWMA_AIB_ control chart with real-life data of glass manufacturing industry using *ρ* = 0.905, *λ*_1_ = 0.2, *λ*_2_ = 0.1, $h_{IAEWMA_{AIB}}=0.5506$ and ARL_0_ = 500.

Here, a real-life data set from the glass industry is considered to illustrate how the proposed IAEWMA_AIB_ and existing AEWMA_AIB_ control charts are practically implemented. Data from Asadzadeh and Kiadaliry [35] are considered here for the glass thickness (*X*) and its impact on stress strength (*Y*) of glass bottles. Anwar *et al*. [27] used this data set for the simultaneous monitoring of process parameters. This data set contains 40 samples, each of size 5, of stress strength (*kg*/*cm*^2^) and thickness (*cm*) (see Table 7). The *X* and *Y* follow BND with ${\hat{\mu }}_{Y}=6.36$, ${\hat{\mu }}_{X}=1.38$, ${\hat{\sigma }}_{Y}=8.92$, ${\hat{\sigma }}_{X}=0.62$ and $\hat{\rho }=0.905$. For the comparison, the proposed IAEWMA_AIB_ control chart is considered along with the existing AEWMA_AIB_ control chart. The parameters of the proposed IAEWMA_AIB_ control chart are taken as *λ*_1_ = 0.2, *λ*_2_ = 0.1, and *ρ* = 0.905 with $h_{IAEWMA_{AIB}}=0.55061$ and ARL_0_ = 500. Similarly, the parameters of AEWMA_AIB_ control chart are taken as *λ* = 0 and *ρ* = 0.905 with $h_{AEWMA_{AIB}}=0.7402$ and ARL_0_ = 500. The existing AEWMA_AIB_ control chart displays the first OOC signal at the 27^th^ sample, whereas the proposed IAEWMA_AIB_ control chart shows at 17^th^ sample (see Table 7 and Figs 4 and 5). Overall, the existing AEWMA_AIB_ control chart detects a total of 5 OOC signals, while the proposed control chart detects 17 OOC signals. The comparison indicates that the proposed IAEWMA_AIB_ control chart is superior to the existing AEWMA_AIB_ control chart in terms of process location monitoring.

**Table 7:** Application of the proposed IAEWMA_AIB_ versus AEWMA_AIB_ control charts for glass manufacturing data.

	1	2	3	4	5	6	7	8	9	10	11	12	13	14	15	16	17	18	19	20
*y*_*i*1_	5.69	29.09	10.3	5.83	8.31	23.36	3.43	19.67	13.62	24.52	25.62	19.14	4.86	17.05	19.5	18.37	23.77	9.41	38.23	5.85
*y*_*i*2_	17.97	22.65	20.13	18.17	18.63	11.68	12.45	20.4	11.9	27.4	16.05	19.85	5.17	24.27	16.15	20.9	29.65	6.41	24.38	31.38
*y*_*i*3_	25.41	18.02	24.88	19.69	8.19	21.8	15.26	13.78	21.82	12.74	22.53	19.48	19.44	9.45	25.18	16.43	16.46	36.95	17.04	18.34
*y*_*i*4_	23.08	22.76	4.47	13.4	24.23	14.89	18.64	28.32	30.31	31.33	27.31	11.65	15.31	9.12	26.8	17.51	26.54	2.66	24.64	10.06
*y*_*i*5_	18.13	24.29	15.56	9.54	15.04	7.16	14.16	26.15	23.87	17.29	7.23	23.12	17.16	18.61	7.98	18.78	24.78	24.03	10.14	20.37
*x*_*i*1_	0.27	2.22	0.75	0.28	0.61	1.74	0.12	1.54	0.98	1.91	2.06	1.50	0.20	1.31	1.54	1.46	1.77	0.67	3.26	0.37
*x*_*i*2_	1.38	1.69	1.57	1.43	1.49	0.85	0.91	1.61	0.86	2.15	1.24	1.55	0.20	1.86	1.24	1.61	2.31	0.43	1.89	2.42
*x*_*i*3_	2.01	1.40	1.97	1.55	0.61	1.64	1.19	1.02	1.65	0.94	1.69	1.54	1.52	0.68	1.98	1.25	1.26	2.57	1.31	1.45
*x*_*i*4_	1.71	1.70	0.19	0.97	1.86	1.12	1.50	2.19	2.33	2.39	2.14	0.84	1.20	0.66	2.12	1.35	2.12	0.04	1.92	0.74
*x*_*i*5_	1.41	1.87	1.20	0.72	1.14	0.49	1.05	2.1	1.79	1.34	0.54	1.74	1.33	1.47	0.55	1.50	1.94	1.84	0.74	1.60
AEWMA_AIB_	0.166	0.526	0.472	0.431	0.394	0.410	0.362	0.421	0.504	0.584	0.597	0.604	0.557	0.528	0.536	0.529	0.622	0.672	0.689	0.687
IAEWMA_AIB_	0.083	0.235	0.237	0.220	0.204	0.239	0.208	0.282	0.380	0.471	0.496	0.513	0.475	0.454	0.470	0.470	0.569	0.623	0.645	0.648
	21	22	23	24	25	26	27	28	29	30	31	32	33	34	35	36	37	38	39	40
*y*_*i*1_	11.23	6.01	21.06	18.84	27.09	14.87	34.2	22.41	11.25	23.88	19.77	11.2	26.68	10.81	21.91	13.53	8.058	11.31	10.41	18.97
*y*_*i*2_	15.18	13.3	17.12	8.52	14.27	16.5	14.6	12.79	21.88	29.27	9.13	10.51	14.27	14.79	19.12	8.507	5.943	3.932	16.74	8.048
*y*_*i*3_	18.17	31.69	14.48	9.72	28.68	21.66	28.73	26.39	16.99	26.94	22.28	10.67	31.1	4.303	16.37	17.12	23.62	12.43	23.53	23.23
*y*_*i*4_	17.85	7.2	20.03	17.15	13.62	13.87	13.96	22.14	29.21	25.74	26	11.62	6.022	21.78	12.35	16.23	6.201	10.69	15.83	12.27
*y*_*i*5_	19.16	16.62	11.72	25.27	23.49	24.73	14.9	5.38	19.22	11.47	12.77	11.7	9.986	21.45	11.88	3.364	5.147	6.87	19.23	9.489
*x*_*i*1_	0.76	0.41	1.64	1.5	2.14	1.12	2.57	1.68	0.77	1.81	1.77	1.09	2.66	1.07	2.19	1.48	0.68	1.11	0.84	1.65
*x*_*i*2_	1.15	0.96	1.32	0.64	1.07	1.28	1.09	0.94	1.67	2.29	0.75	0.85	1.48	1.49	1.67	0.70	0.46	0.16	1.56	0.61
*x*_*i*3_	1.45	2.49	1.07	0.73	2.2	1.64	2.2	2.12	1.29	2.12	2.27	0.91	2.78	0.18	1.54	1.58	2.44	1.47	2.40	2.33
*x*_*i*4_	1.37	0.50	1.56	1.33	0.99	1.03	1.04	1.67	2.23	2.07	2.65	1.15	0.48	2.02	1.45	1.54	0.50	1.05	1.50	1.33
*x*_*i*5_	1.51	1.29	0.86	1.99	1.76	1.94	1.13	0.26	1.51	0.80	1.47	1.22	0.81	1.88	1.30	0.15	0.36	0.52	1.74	0.79
AEWMA_AIB_	0.672	0.646	0.645	0.609	0.693	0.710	0.876	0.905	0.966	1.082	0.733	0.477	0.447	0.419	0.129	0.056	0.212	0.391	0.644	0.849
IAEWMA_AIB_	0.636	0.615	0.617	0.583	0.670	0.689	0.774	0.799	0.840	0.910	0.579	0.339	0.148	0.004	0.252	0.399	0.521	0.543	0.560	0.576

### 6.2: Application 2

This subsection illustrates how the proposed control charts can be used in practice to monitor the stability of groundwater physicochemical parameters. The stability of the soil water parameters is always desirable to industrial processes, crop yields and drinking water, all of which ultimately impact industrial production, crop production and human health. In particular, cultivation yield is influenced by certain factors, such as colour, acidity, hardness, pH, sulphite and temperature. We consider two physio-chemical groundwater parameters, including total dissolved solids and the total water hardness, to show the applicability of the proposed control chart. More precisely, total dissolved solids is a study variable *Y* (measured in terms of electric conductivity (EC)). In contrast, total hardness of water is an auxiliary variable *X* (measured in terms of calcium magnesium carbonates). We consider groundwater (used for crop irrigation) in District Rahim Yar Khan, Pakistan, to demonstrate the significance of the proposed location control charts. The data is taken from [36], and it is based on 30 different locations from each location, a sample of size five is collected. The *X* and *Y* follow BND with *μ*_*Y*_ = 836.06, *μ*_*X*_ = 4.93, $\sigma_{Y}^{2}=1000$, $\sigma_{X}^{2}=1.53$ and *ρ* = 0.50.

**Fig 6:**
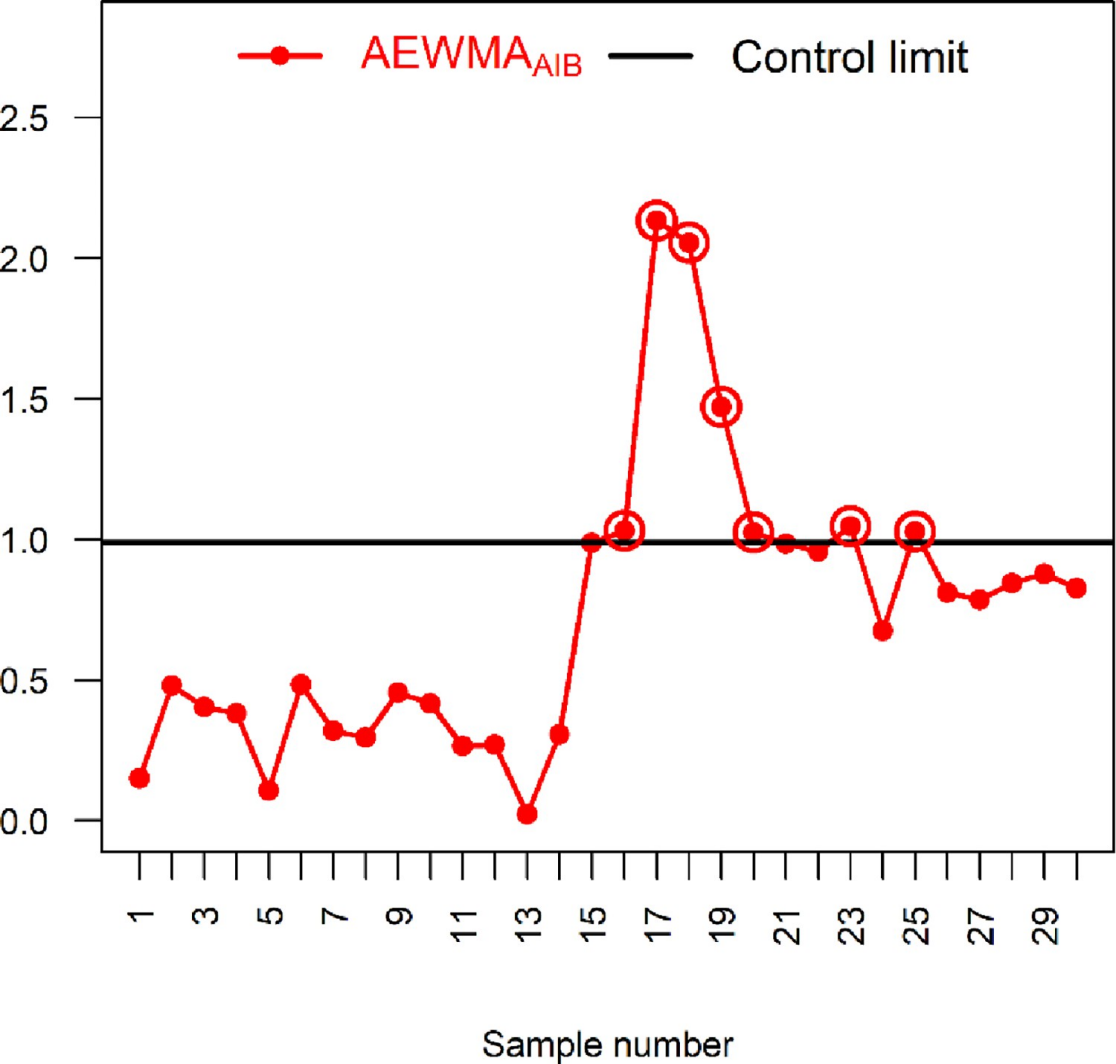
AEWMA_AIB_ chart with real-life data of ground water using *ρ* = 0.50, *λ* = 0.2, $h_{AEWMA_{AIB}}=0.9879$ and ARL_0_ = 500.

**Fig 7:**
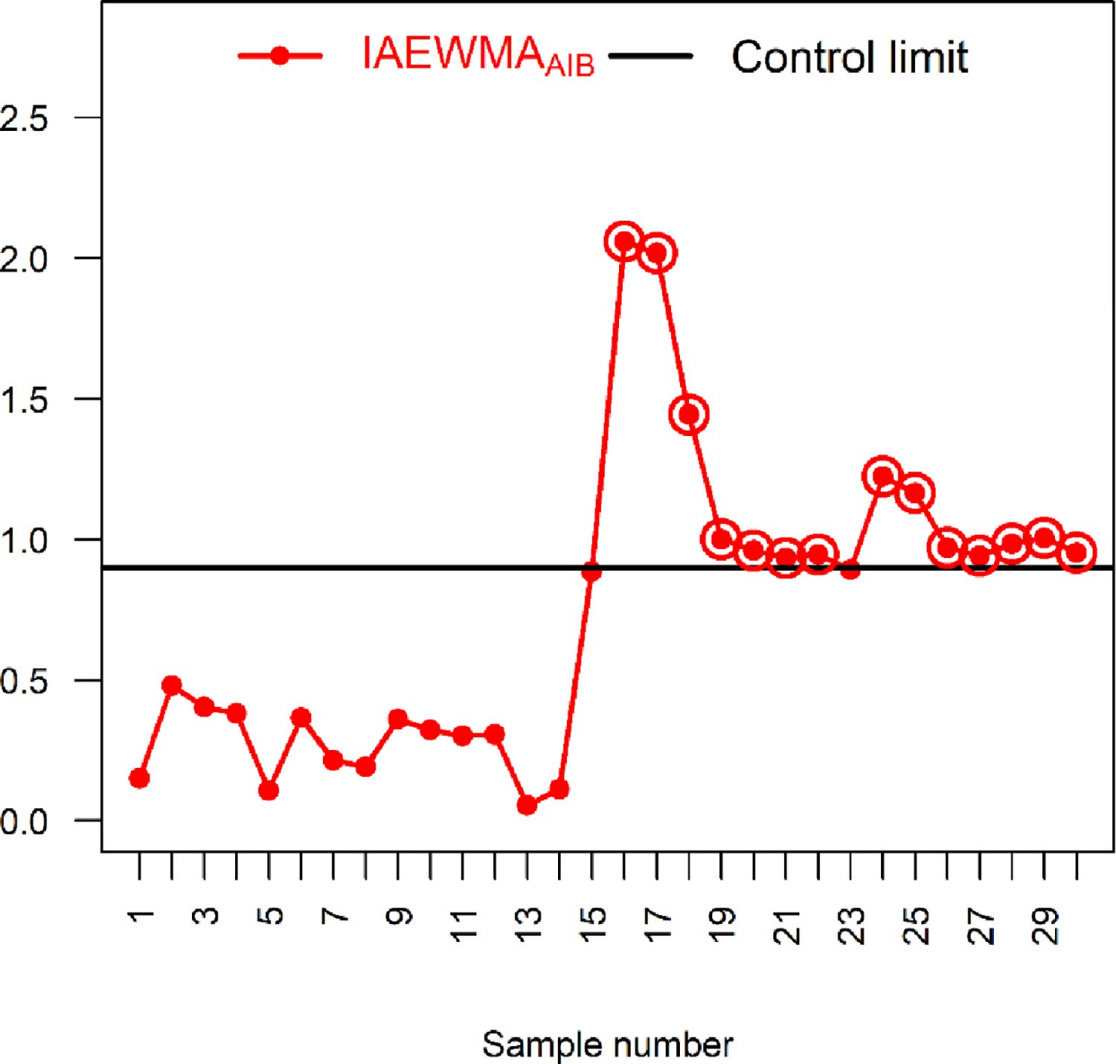
Proposed IAEWMA_AIB_ control chart with real-life data of ground water using *ρ* = 0.50, *λ*_1_ = 0.2, *λ*_2_ = 0.75, $h_{IAEWMA_{AIB}}=0.9002$ and ARL_0_ = 500.

We consider the proposed IAEWMA_AIB_ control chart along with the existing AEWMA_AIB_ control chart for the practical implementation. The parameters of the proposed IAEWMA_AIB_ control chart are taken as *λ*_1_ = 0.2, *λ*_2_ = 0.75, *ρ* = 0.50 with $h_{IAEWMA_{AIB}}=0.777$ and ARL_0_ = 500. Similarly, the parameters of AEWMA_AIB_ control chart are taken as *λ* = 0.2 and *ρ* = 0.50 with $h_{AEWMA_{AIB}}=0.9879$ and ARL_0_ = 500. From Table 8 and Figs 6 and 7, it can be seen that the existing AEWMA_AIB_ control chart detects seven OOC signals whereas the proposed IAEWMA_AIB_ control chart displays 16 OOC signals. Hence, the proposed IAEWMA_AIB_ control chart is more efficient for the monitoring the stability of groundwater physicochemical parameters.

**Table 8:** Application of the proposed IAEWMA_AIB_ versus AEWMA_AIB_ control charts for ground water data.

	1	2	3	4	5	6	7	8	9	10	11	12	13	14	15
*y*_*i*1_	860	846	850	806	750	744	870	888	775	868	825	792	933	910	950
*y*_*i*2_	817	830	828	835	792	720	810	825	792	885	810	811	870	909	960
*y*_*i*3_	880	845	879	780	760	790	840	860	750	885	825	816	909	888	927
*y*_*i*4_	912	890	803	790	720	775	815	880	812	900	850	845	933	933	990
*y*_*i*5_	856	897	887	757	791	782	820	895	742	860	830	870	925	860	890
*x*_*i*1_	6.4	3.8	7.5	4.7	4.2	3.2	1.9	4.5	5.0	3.5	2.5	5.8	6.0	5.3	7.0
*x*_*i*2_	5.8	3.5	6.3	2.7	5.8	3.6	1.8	5.1	4.0	3.8	1.8	5.9	6.5	7.2	6.3
*x*_*i*3_	4.4	3.6	6.0	3.0	4.6	3.5	1.7	4.8	4.9	4.0	1.7	4.7	6.0	7.3	6.6
*x*_*i*4_	6.5	3.8	6.2	3.7	3.7	4.9	2.0	4.7	3.0	4.5	1.8	5.3	6.3	6.3	7.3
*x*_*i*5_	4.8	4.2	6.9	5.1	3.0	4.7	3.0	5.0	3.7	3.2	2.0	6.0	6.6	6.2	5.1
AEWMA_AIB_	0.151	0.480	0.404	0.382	0.107	0.483	0.320	0.296	0.455	0.416	0.265	0.271	0.022	0.307	0.988
IAEWMA_AIB_	0.151	0.480	0.404	0.382	0.107	0.365	0.214	0.192	0.361	0.323	0.302	0.307	0.055	0.113	0.886
	16	17	18	19	20	21	22	23	24	25	26	27	28	29	30
*y*_*i*1_	967	920	850	760	781	870	858	745	990	872	830	750	880	880	747
*y*_*i*2_	960	909	828	725	740	845	873	773	933	907	780	790	840	918	730
*y*_*i*3_	914	867	879	790	798	835	880	730	940	914	867	860	867	915	730
*y*_*i*4_	895	890	803	795	803	828	900	780	980	830	820	860	909	890	720
*y*_*i*5_	935	945	887	750	812	773	820	732	933	856	825	810	867	840	790
*x*_*i*1_	5.0	6.4	7.5	5.7	4.0	6.8	3.1	3.8	5.25	7.0	5.7	5.8	5.0	6.3	2.9
*x*_*i*2_	6.0	6.2	6.3	5.5	3.6	6.3	3.3	4.8	6.0	6.7	5.5	4.8	4.3	6.8	3.6
*x*_*i*3_	4.5	5.2	6.0	5.9	5.0	6.4	3.0	3.6	4.7	7.3	5.9	5.5	4.9	6.6	2.2
*x*_*i*4_	6.5	5.9	6.2	5.8	5.0	6.2	3.5	3.9	4.0	6.4	5.7	5.3	5.2	6.8	3.6
*x*_*i*5_	6.4	7.2	6.9	5.6	5.2	6.0	3.7	4.3	6.3	6.0	5.6	5.0	4.8	6.0	3.2
AEWMA_AIB_	1.031	2.132	2.055	1.471	1.024	0.985	0.957	1.046	0.674	1.027	0.811	0.785	0.844	0.878	0.827
IAEWMA_AIB_	2.060	2.019	1.443	1.000	0.960	0.933	0.947	0.893	1.223	1.164	0.970	0.941	0.985	1.005	0.952

## 7: Summary, conclusions, and recommendations

The Adaptive exponentially weighted moving average (AEWMA) control chart is an advanced form of the classical EWMA control chart to track small to large shifts in the process. The objective of this study is to improve the detection ability of the existing auxiliary information-based (AIB) AEWMA (AEWMA_AIB_) control chart and propose an enhanced AIB AEWMA, symbolized as IAEWMA_AIB_ control chart for efficient monitoring of process location shift. The proposed IAEWMA_AIB_ control chart is designed by estimating unknown process location shift with hybrid EWMA statistic, and then smoothing constant is adaptively updated. To evaluate the performance of the proposed IAEWMA_AIB_ control chart against other control charts, Monte Carlo simulations technique is used for numerical results. Performance comparison tools like average run length, extra quadratic loss, performance comparison index, and relative average run length are used. The analysis based on performance evaluation measures and visual presentation reveals the proposed IAEWMA_AIB_ control chart outperforms against AIB CUSUM, AIB EWMA, AEWMA_AIB_, HEWMA, and AIB improved adaptive Crosier CUSUM. Furthermore, it is vital to mention, that the proposed IAEWMA_AIB_ control chart converges to AEWMA and AEWMA_AIB_ control charts at the specific values of parameters. Finally, two real-life applications for users and practitioners are also provided to show the proposed study from a practical perspective. This study can be extended for non-normal distribution and the multivariate case.

### Appendix

Let $\left\{{\hat{\delta }}_{t}^{*}\right\}$ is a sequence based on {*T*_*t*_} for IC process, then the shift estimator using EWMA statistic is

$${\hat{\delta }}_{t}^{*}=\lambda_{1}T_{t}+(1-\lambda_{1}){\hat{\delta }}_{t-1}^{*}$$ (A-1)

The ${\hat{\delta }}_{t}^{*}$ can be written as

$${\hat{\delta }}_{t}^{*}=\lambda_{1}\sum_{j=0}^{t-1}{\left(1-\lambda_{1}\right)}^{j}T_{t-j}+{\left(1-\lambda_{1}\right)}^{t}{\hat{\delta }}_{0}^{*}$$

Let $\left\{{\hat{\delta }}_{t}^{**}\right\}$ is another sequance defined on $\left\{{\hat{\delta }}_{t}^{*}\right\}$, then the estimator of ${\hat{\delta }}_{t}^{**}$ using HEWMA statistic is

$${\hat{\delta }}_{t}^{**}=\lambda_{2}{\hat{\delta }}_{t}^{*}+(1-\lambda_{2}){\hat{\delta }}_{t-1}^{**}$$ (A-2)

Similarly, the ${\hat{\delta }}_{t}^{**}$ can be written as

$${\hat{\delta }}_{t}^{**}=\lambda_{2}\sum_{i=0}^{t-1}{\left(1-\lambda_{2}\right)}^{i}{\hat{\delta }}_{t-i}^{*}+{\left(1-\lambda_{2}\right)}^{t}{\hat{\delta }}_{0}^{**}.$$

Where (*λ*_2_, *λ*_2_)∈(0,1] are the smoothing constants. The initial values of ${\hat{\delta }}_{t}^{*}$ and ${\hat{\delta }}_{t}^{**}$ are zero, i.e., ${\hat{\delta }}_{0}^{*}={\hat{\delta }}_{0}^{**}=0$.


$${\hat{\delta }}_{t}^{**}=\lambda_{1}\lambda_{2}\sum_{i=0}^{t-1}{\left(1-\lambda_{2}\right)}^{i}\sum_{j=0}^{t-i-1}{\left(1-\lambda_{1}\right)}^{j}T_{t-i-j}$$ (A-3)

Using Mathematica, we can get the following

$${\hat{\delta }}_{t}^{**}=\lambda_{1}\lambda_{2}\sum_{i=1}^{t}\left[{\left(1-\lambda_{1}\right)}^{t-i}\sum_{j=0}^{t-j}{\left(\frac{1-\lambda_{2}}{1-\lambda_{1}}\right)}^{j}T_{i}\right]$$ (A-4)

Taking expectation on the both side of (A-4),

$${E(\hat{\delta }}_{t}^{**})=\lambda_{1}\lambda_{2}\sum_{i=1}^{t}\left[{\left(1-\lambda_{1}\right)}^{t-i}\sum_{j=0}^{t-j}{\left(\frac{1-\lambda_{2}}{1-\lambda_{1}}\right)}^{j}{E(T}_{i})\right]$$

$${E(\hat{\delta }}_{t}^{**})=\lambda_{1}\lambda_{2}\sum_{i=1}^{t}[{\left(1-\lambda_{1}\right)}^{t-i}\sum_{j=0}^{t-j}{\left(\frac{1-\lambda_{2}}{1-\lambda_{1}}\right)}^{j}]\delta^{*}$$ (A-5)

Now, using “FullSimplify” command in Mathematica, we can get the following

$$E({\hat{\delta }}_{t}^{**})=\left[\frac{\lambda_{1}-\lambda_{2}+\lambda_{2}{\left(1-\lambda_{1}\right)}^{t}-\lambda_{1}{\left(1-\lambda_{2}\right)}^{t+1}-\lambda_{1}{\lambda_{2}(1-\lambda_{1})}^{t}}{\lambda_{1}-\lambda_{2}}\right]\delta^{*}$$ (A-6)

$$E({\hat{\delta }}_{t}^{**})=c\delta^{*}$$ (A-7)

$$Where\;c=\left[\frac{\lambda_{1}-\lambda_{2}+\lambda_{2}{\left(1-\lambda_{1}\right)}^{t}-\lambda_{1}{\left(1-\lambda_{2}\right)}^{t+1}-\lambda_{1}{\lambda_{2}(1-\lambda_{1})}^{t}}{\lambda_{1}-\lambda_{2}}\right]$$

$$\frac{E({\hat{\delta }}_{t}^{**})}{c}=\delta^{*}$$ (A-8)

Which is the unbiased estimator.

## Supporting information

S1 Data (PDF)

S2 Data (PDF)
