# Supplementary material for: Improved adaptive EWMA control chart for process location with applications in groundwater physicochemical parameters and glass manufacturing industry
Source: PLoS One. 2022 Aug 22;17(8):e0272584. doi: 10.1371/journal.pone.0272584 (PMC9394848; doi:10.1371/journal.pone.0272584)
Supplement: S1 Data — (PDF) [file pone.0272584.s001.pdf]

Computers & Industrial Engineering 156 (2021) 107284

Contents lists available at ScienceDirect

Computers & Industrial Engineering

journal homepage: www.elsevier.com/locate/caie

Mixed memory control chart based on auxiliary information for simultaneously monitoring of process parameters: An application in glass field

Syed Masroor Anwar [a,b,*], Muhammad Aslam [a], Babar Zaman [c,d], Muhammad Riaz [e]

[a] Department of Mathematics and Statistics, Riphah International University, Pakistan
[b] Department of Statistics, University of Azad Jammu and Kashmir, Pakistan
[c] Department of Mathematical Sciences, Universiti Teknologi Malaysia, Skudai, Malaysia
[d] Department of Mathematics, College of Science, University of Hafr Al Batin, Hafr Al Batin, Saudi Arabia
[e] Department of Mathematics and Statistics, King Fahd University of Petroleum and Minerals, Dahran, Saudi Arabia

ARTICLE INFO

Keywords:
Average run length
Auxiliary information
Memory control charts
Monte Carlo simulations
Simultaneously

ABSTRACT

Cumulative sum (CUSUM) and exponentially weighted moving (EWMA) control charts, known as memory control charts, are famous to monitor a small-to-moderate shift in the process parameters (location and/or dispersion). The EWMA control chart based on auxiliary information denoted as $\text{EWMA}_{\text{AIB}}^{(1)}$ and $\text{EWMA}_{\text{AIB}}^{(2)}$ are advanced forms of the classical EWMA control chart to monitor the process location and dispersion, respectively. Likewise, the combined mixed EWMA-CUSUM (CMEC) control chart is used to simultaneously monitor the process parameters. This study presents the CMEC control chart based on auxiliary information, symbolized as CMEC_{AIB} control chart for simultaneous monitoring of the process parameters. The proposed CMEC_{AIB} control chart used $\text{EWMA}_{\text{AIB}}^{(1)}$ and $\text{EWMA}_{\text{AIB}}^{(2)}$ control charts plotting statistics as inputs in the classical CUSUM control charts. The Monte Carlo simulation is used as a computational technique for numerical results. The proposed CMEC_{AIB} control chart based on average run length performance measure is evaluated against other control charts such as CMEC, combined double mixed EWMA-CUSUM, combined CUSUM, maximum double EWMA, maximum EWMA based on auxiliary information ($\text{MaxEWMA}_{\text{AIB}}$) control and maximum Hybrid EWMA based on auxiliary information ($\text{MaxHEWMA}_{\text{AIB}}$) control charts. The comparison revealed the superiority of the proposed CMEC_{AIB} control chart. Besides, the CMEC_{AIB} control chart performs better as the correlation coefficient increases. Likewise, the proposed CMEC_{AIB} control chart has a better diagnostic ability for possible directions of changes against other control charts. It is vital to mention that some existing control charts such as classical CUSUM, CMEC, and maximum CUSUM are special cases of proposed CMEC_{AIB} control chart with the specific parameter's values. Finally, to demonstrate the vitality of the proposed study from a practical point of view, a real-life application in the glass bottle manufacturing industry is also provided for users and practitioners.

1. Introduction

Variations are inevitable for all types of manufacturing and non-manufacturing process parameters (location and/or dispersion). Variations can be categorized as a special cause and a random cause of variations. The random cause of variations are harmless and integral part of every stable process that cannot be eliminated entirely. On the contrary, the special cause of variations trigger an inconsistency in the process's performance and undermines the validity of the process. Therefore, it is necessary to take corrective action(s) timely to handle the special cause of variations to ensure the process stability. The magnitude of the special cause of variations that occurs in the process parameters is termed as a shift. Control charts are the famous tool of statistical process control (SPC) toolkit to identify a shift timely. Shewhart control charts proposed by Shewhart (1931) are famous for detecting a large shift in the process parameters effectively, and it is also known as memory-less control charts. On the other hand, memory control charts such as cumulative sum (CUSUM) and exponentially weighted moving average (EWMA) control charts recommended by Page (1954) and Roberts (1959), respectively, are famous for handling small-to-moderate shift.

Modifications and enhancements to the basic structures of the

* Corresponding author at: Department of Statistics, University of Azad Jammu and Kashmir, Pakistan.
E-mail addresses: masroorstatistics@gmail.com (S.M. Anwar), m.aslam@riphah.edu.pk (M. Aslam), riazm@kfupm.edu.sa (M. Riaz).

https://doi.org/10.1016/j.cie.2021.107284
Received 19 June 2020; Received in revised form 15 February 2021; Accepted 26 March 2021
Available online 6 April 2021
0360-8352/© 2021 Elsevier Ltd. All rights reserved.

Nomenclature

Acronyms

ARL	Average run length
ARL_0	In-control average run length
ARL_1	Out-of-control average run length
CUSUM	Cumulative sum
CMEC	Combined mixed EWMA-CUSUM
CDMEC	Combined double mixed EWMA-CUSUM
CC	Combined CUSUM
$CMEC_{AIB}$	Auxiliary-information-based CMEC
CDF	Cumulative distribution function
EWMA	Exponentially weighted moving
$EWMA^{(1)}_{AIB}$	Auxiliary-information-based EWMA for process location
$EWMA^{(2)}_{AIB}$	Auxiliary-information-based EWMA for process dispersion
IID	Independent identically distributed
$LCL_{EWMA^{(1)}_{AIB}}$	Lower control limit of $EWMA^{(1)}_{AIB}$
$LCL_{EWMA^{(2)}_{AIB}}$	Lower control limit of $EWMA^{(2)}_{AIB}$
MaxEWMA	Maximum EWMA for joint monitoring
MaxCUSUM	Maximum CUSUM for joint monitoring
MEC	Mixed EWMA-CUSUM
MCE	Mixed CUSUM-EWMA
MxMCE	Auxiliary-information-based MCE
MxMEC	Auxiliary-information-based MEC
$MaxEWMA_{AIB}$	Auxiliary-information-based Maximum EWMA chart
MaxDEWMA	Maximum double EWMA
$MaxHEWMA_{AIB}$	Auxiliary-information-based Max hybrid EWMA chart
TMC	Thickness measuring system for container glass
SPC	Statistical process control
$UCL_{EWMA^{(1)}_{AIB}}$	Upper control limit of $EWMA^{(1)}_{AIB}$
$UCL_{EWMA^{(2)}_{AIB}}$	Upper control limit of $EWMA^{(2)}_{AIB}$

Symbols

Y	Variable of interest
X	Auxiliary variable
δ_μ	Amount of shift in process location
δ_γ	Amount of shift in process dispersion
$U_{Y(t)}$	Transformed variable for process location
$V_{Y(t)}$	Transformed variable for process location
$G(\cdot)$	Cumulative Distribution Function
$\Phi^{-1}(\cdot)$	Inverse CDF of the standard normal distribution
$\overline{Y}$	Sample mean
S^2_Y	Sample variance
μ_Y	Mean of variable Y
μ_X	Mean of variable X
σ_Y	Standard deviation of Y
σ_X	Standard deviation of X
ρ	Correlation coefficient of variables Y and X
$D^{(1)}_{Y(t)}$	Auxiliary information-based difference estimator for process location
n	Number sample
$D^{(2)}_{Y(t)}$	Auxiliary information-based difference estimator for the process dispersion
ρ^*	Correlation coefficient between $V_{Y(t)}$ and $V_{X(t)}$
$A_{Y(t)}$	Transformed variable based on $D^{(1)}_{Y(t)}$
$B_{Y(t)}$	Transformed variable based on $D^{(2)}_{Y(t)}$
$A^{(1)}_{Y(t)}$	$EWMA^{(1)}_{AIB}$ statistic for location
$B^{(2)}_{Y(t)}$	$EWMA^{(2)}_{AIB}$ statistic for dispersion
λ_1	Smoothing constant of $EWMA^{(1)}_{AIB}$ and $EWMA^{(2)}_{AIB}$
$L_{EWMA^{(1)}_{AIB}}$	Control limit coefficient of $EWMA^{(1)}_{AIB}$
$L_{EWMA^{(2)}_{AIB}}$	Control limit coefficient of $EWMA^{(2)}_{AIB}$
$U^*_{(t)}$	Classical EWMA statistic for location
$V^*_{(t)}$	Classical EWMA statistic for dispersion
λ	Smoothing constant of EWMA for location and EWMA for dispersion
k	Constant of reference value in CMEC
h	Constant of decision interval in CMEC
$CMECC^{\pm}_{(t)}$	Location Plotting statistics of CMEC
$CMECD^{\pm}_{(t)}$	Dispersion Plotting statistics of CMEC
k_1	Constant of reference value in $CMEC_{AIB}$
h_1	Constant of decision interval in $CMEC_{AIB}$
$P^{\pm}_t$	Location Plotting statistics of $CMEC_{AIB}$
$Q^{\pm}_t$	Dispersion Plotting statistics of $CMEC_{AIB}$
$H_{1(t)}$	Decision interval of $CMEC_{AIB}$
$K_{1(t)}$	Reference values of $CMEC_{AIB}$

classical memory control charts through different methods and techniques are continuously practiced. One such method is a mixed technique that is used to enhance the performance of the classical memory control charts. For example, Abbas, Riaz, and Does (2012a, 2012b) presented the concept of mixed EWMA-CUSUM (MEC) control charts for monitoring the process parameters. Similarly, Zaman, Riaz, Abbas, and Does (2015) and Zaman, Abbas, Riaz, and Lee (2016) proposed the mixed CUSUM-EWMA (MCE) control charts to diagnose the shifts in the process parameters. Besides, Anwar, Aslam, Riaz, and Zaman (2020) extended the MCE and MEC control charts based on auxiliary information, named as MxMCE and MxMEC control charts, respectively, to monitor the process location. More insights into this direction can be seen in the studies of Ajadi and Riaz (2017), Osei-Aning, Abbasi, and Riaz (2017), Adeoti and Malela-Majika (2019), and references therein.

Despite the mixed technique, the use of auxiliary information with the process variables to monitor the process parameters has attracted considerable attention in the last few years. For example, in a wire production process for studying the tensile strength of the wire, the outside diameter of the wire can be referred to as an auxiliary variable. Likewise, the half-life of fissile material can be treated as an auxiliary variable in the process monitoring of nuclear power generation. Also, in a chemical reaction, monitoring the activation energy of molecules can be served as the auxiliary variable (Hussain, Song, Ahmad, & Riaz, 2019). In this aspect, Abbas, Riaz, and Does (2014) presented an EWMA control chart based on auxiliary information for the process location, denoted as $EWMA^{(1)}_{AIB}$ control chart. Similarly, Haq (2017a) and Haq (2017b) introduced the auxiliary information based EWMA, represented as $EWMA^{(2)}_{AIB}$ control chart for the process dispersion, and the maximum EWMA based on auxiliary information ($MaxEWMA_{AIB}$) control chart for the simultaneous monitoring of the process parameters, respectively. Sanusi, Abbas, and Riaz (2017) proposed a CUSUM control chart for the efficient monitoring of a process location in the presence of auxiliary information. Likewise, Anwar, Aslam, Ahmad, and Riaz (2020) have also used auxiliary information for efficient monitoring of the process location using a modified-EWMA control chart. Recently, Javaid, Noor-ul-Amin, and Hanif (2020) suggested auxiliary information based maximum hybrid EWMA ($MaxHEWMA_{AIB}$) control chart for the

simultaneous monitoring of the process parameters. For more detail see Riaz (2008), Sodipo (2010), Singh and Solanki (2013), Riaz, Mehmood, Ahmad, and Abbasi (2013), Grover and Kaur (2014), Ahmad, Abbasi, Riaz, and Abbas (2014), Adegoke, Riaz, Sanusi, Smith, and Pawley (2017), Abbasi and Haq (2019), and Aslam and Anwar (2020) references therein.

The simultaneous monitoring of the process parameters is fairly commonplace, as it is not known priory that the shift either occurs in the process location, dispersion, or in both. For example, in the surface mount assembly process, a typical situation may arise where the process parameters diverge from the target values because solder paste height may be printed unevenly across the board as a result, which the squeegee may not be appropriately leveled, or a stencil is not properly fixed, or a support block for a printed circuit board is not appropriately aligned (Gan, Ting, & Chang, 2004). From this perspective, Chen and Cheng (1998) proposed the Max control chart for simultaneous monitoring of the process parameters. Chen, Cheng, and Xie (2016) designed the maximum EWMA (MaxEWMA) control chart under the normal and inverse normal transformations for monitoring process parameters. Zaman, Riaz, and Lee (2016) extended the studies of Abbas et al. (2012a, 2012b) for simultaneous monitoring of the process parameters, named as a combined MEC (CMEC) control chart. Noor-ul-Amin, Tariq, and Hanif (2019) presented the MaxEWMA control chart using auxiliary information for monitoring process location and coefficient of variation jointly. Numerous authors such as Gan (1995), Sheu, Huang, and Hsu (2012), McCracken and Chakraborti (2013), Mukherjee, McCracken, and Chakraborti (2015), Chen, Cheng, and Xie (2018), and Chao-Wen and Reynolds (2018) have proposed control charts for efficient monitoring of process parameters.

As mentioned before, the CMEC control chart has been suggested by Zaman, Riaz, et al. (2016) for simultaneous monitoring of the process parameters when the variable of interest is univariate and free from any other source of information. Therefore, the CMEC control chart may not provide proper detection of shifts if the variable of interest also carrying auxiliary information. So, to address this point, this study is designed the CMEC control chart based on auxiliary information, symbolized as a $\mathrm{CMEC_{AIB}}$ control chart for the simultaneous monitoring of the process parameters. The main objective of this study is to propose a $\mathrm{CMEC_{AIB}}$ control chart that is used for the simultaneous monitoring of process location and dispersion when the variable of interest also carrying auxiliary information. In more details, the $\mathrm{CMEC_{AIB}}$ control chart is based on $\mathrm{EWMA}^{(1)}_{\mathrm{AIB}}$ and $\mathrm{EWMA}^{(2)}_{\mathrm{AIB}}$ control charts. The plotting statistics of the $\mathrm{EWMA}^{(1)}_{\mathrm{AIB}}$ and $\mathrm{EWMA}^{(2)}_{\mathrm{AIB}}$ control charts are used as input for the classical CUSUM control charts to design $\mathrm{CMEC_{AIB}}$ control chart. The Monte Carlo simulation method is used as a computational technique for numerical results. The performance of the $\mathrm{CMEC_{AIB}}$ control chart is evaluated based on average run length (ARL) measure against other control charts such as CMEC, combined double MEC (CDMEC), combined CUSUM (CC), maximum double EWMA (MaxDEWMA),$\mathrm{MaxEWMA_{AIB}}$, and $\mathrm{MaxHEWMA_{AIB}}$ control charts. Besides, the proposed $\mathrm{CMEC_{AIB}}$ control chart is also implemented with real-life data to show the significance for practical importance.

The rest of the study is organized as follows: variable of interest, transformations, auxiliary information, transformations based on auxiliary information, $\mathrm{EWMA}^{(1)}_{\mathrm{AIB}}$ control chart for process location, $\mathrm{EWMA}^{(2)}_{\mathrm{AIB}}$ control chat for process dispersion, and CMEC control chart for simultaneous monitoring of the process parameters are outlined in Section 2. Moreover, Section 3 presents the design structure of the proposed $\mathrm{CMEC_{AIB}}$ control chart, the construction procedure of the proposed $\mathrm{CMEC_{AIB}}$ control chart, identification of directions by diagnostic abilities, and special cases of the proposed $\mathrm{CMEC_{AIB}}$ control chart. Furthermore, Section 4 contains a brief discussion about the evaluation and the performance comparison of the proposed $\mathrm{CMEC_{AIB}}$ control chart against CMEC, CDMEC, CC, MaxDEWMA,$\mathrm{MaxEWMA_{AIB}}$, and $\mathrm{MaxHEWMA_{AIB}}$ control charts. Additionally, Section 5 describes the implementation of the proposed $\mathrm{CMEC_{AIB}}$ control chart with real-life data. Finally, a summary, conclusions, and recommendations are given in Section 6.

2. Existing methods

This section explains the background of the variable of interest in Section 2.1. Section 2.2 introduces transformations. Likewise, Section 2.3 is about auxiliary information, and transformations based on auxiliary information are given in Section 2.4. The methodologies of the $\mathrm{EWMA}^{(1)}_{\mathrm{AIB}}$ and $\mathrm{EWMA}^{(2)}_{\mathrm{AIB}}$ control charts to monitor the shifts in the process parameters are presented in Section 2.5. Additionally, the CMEC control chart for simultaneous monitoring of the process parameters is designed in Section 2.6.

2.1. Variable of interest

Suppose $Y(Y\ N(\mu_Y+\delta_\mu\sigma_Y,\delta_\gamma\sigma_Y))$ be the process variable follows a normal distribution with mean $\mu_Y+\delta_\mu\sigma_Y$ and standard deviation $\delta_\gamma\sigma_Y$. If $\delta_\mu=0$ and $\delta_\gamma=1$, the process is in-control; otherwise, it is out-of-control. Let $\overline{Y}_{(t)}=\sum_{i=1}^{n}Y_{(it)}/n$ and $S^2_{Y(t)}=\sum_{i=1}^{n}\left(Y_{(it)}-\overline{Y}_{(t)}\right)^2/(n-1)$ be the sample mean and variance of$Y$, respectively of ith observation of tth sample of size n. So, for the in-control situation, the $\overline{Y}_{(t)}$ and $S^2_{Y(t)}$ are mutually independent identically distributed (IID), that is $\overline{Y}_{(t)}\ N(\mu_Y,\sigma^2_Y/n)$ and$S^2_{Y(t)}\ (\sigma^2_Y/n-1)(\chi^2_{n-1})$.

2.2. Transformations

Classical memory (CUSUM and EWMA) control charts are generally designed to handle the process location shift. The use of $S^2_{Y(t)}$ statistic as input for the memory control charts to monitor the process dispersion may lead to inefficient results. Therefore, to solve this issue, Quesenberry (1995) suggested the following transformations.

$$U_{Y(t)}=\frac{\overline{Y}_{(t)}-\mu_Y}{\sigma_Y/\sqrt{n}} \tag{1}$$

$$V_{Y(t)}=\Phi^{-1}\left(G\left(\frac{(n-1)S^2_{Y(t)}}{\sigma^2_Y};n-1\right)\right) \tag{2}$$

where $G(\cdot;n-1)$ is the cumulative distribution function (CDF) of the chi-squared distribution with $n-1$ degrees of freedom, and $\Phi^{-1}(\cdot)$ is the inverse CDF of the standard normal distribution. As for the in-control situation, the $\overline{Y}_t$ and $S^2_{Y(t)}$ statistic are IID, therefore the $U_{Y(t)}$ and $V_{Y(t)}$ statistics are also IID and have standard normal distributions (i.e., $U_{Y(t)}\ N(0,1)$ and $V_{Y(t)}\ N(0,1)$).

2.3. Auxiliary information

A variable known to all population units (other than the study variable) may improve the sampling plan and improve the interest parameter's precision called the auxiliary variable. The use of auxiliary information for precise estimation of population parameters is famous in survey sampling and estimation. A control chart's performance can be improved with efficient auxiliary information-based process parameters estimator(s) (Abbasi & Haq, 2019; Ahmad et al., 2014; Haq, 2018). In the process monitoring, the auxiliary variables can be categorized into two types. *(a)*; the process where one or more auxiliary variables are correlated with the study variable and the process shifts in location or dispersion parameter occurs only in the study variable and does not affect the auxiliary variable. *(b)*; the other situation in the process monitoring where the shifts occur in the study variable may affect the auxiliary variables. Anwar, Aslam, Riaz, et al. (2020) provided

various examples of both types of auxiliary variables. The current study deals with the first situation where any shift in the study variable does not affect the auxiliary variable(s) following (Abbasi & Haq, 2019; Ahmad et al., 2014; Anwar, Aslam, Ahmad, et al., 2020; Anwar, Aslam, Riaz, et al., 2020).

Let suppose X is an auxiliary information variable of Y variable. The X and Y follow a bivariate normal distribution that is $(Y,X)\ N(\mu_Y,\mu_X,\sigma_Y,\sigma_X,\rho)$, where μ_X and σ_X are the mean and standard deviation of X, respectively, and ρ is the correlation coefficient between Y and X variables. Since the process parameters $(\mu_Y,\mu_X,\sigma_Y,\sigma_X,\rho)$ are assumed to be known; it is phase-II process monitoring (Haq, 2018). Let $(Y_{(it)},X_{(it)})$, $i=1,2,\ldots n$ be a random sample of size n at time t, for $t\geq 1$. Haq and Khoo (2016) suggested the difference estimator based on auxiliary information to monitor the process location, and it is given as follows:

$$D^{(1)}_{Y(t)}=\overline{Y}+\beta\left(\mu_X-\overline{X}\right) \tag{3}$$

where $\beta=\rho_{YX}(\sigma_Y/\sigma_X)$. The mean and variance of the $D^{(1)}_{Y(t)}$ are defined as $E(D^{(1)}_{Y(t)})=\mu_Y$ and $Var(D^{(1)}_{Y(t)})=\sigma^2_Y(1-\rho^2)/n$, respectively.

2.4. Transformation based on auxiliary information

Haq (2017b) recommended $V_{Y(t)}$ and $V_{X(t)}$ $(V_{X(t)}=\Phi^{-1}\left[G\left((n-1)S^2_{X(t)}/\sigma^2_X\right)\right]\ N(0,1))$ statistics to develop difference estimator to monitoring the change in the process dispersion (σ^2_Y), and it is defined as follows:

$$D^{(2)}_{Y(t)}=V_{Y(t)}-\rho^{*}V_{X(t)} \tag{4}$$

where $\rho^{*}$is the correlation coefficient between $V_{Y(t)}$ and $V_{X(t)}$ variables. The mean and variance of the $D^{(2)}_{Y(t)}$ are $E(D^{(2)}_{Y(t)})=0$ and $Var(D^{(2)}_{Y(t)})=1-\rho^{*2}$, respectively. The variable of interest to monitor the process location using $D^{(1)}_{Y(t)}$ statistic is defined as follows:

$$A_{Y(t)}=\frac{D^{(1)}_{Y(t)}-\mu_Y}{\sqrt{\frac{\sigma^2_Y(1-\rho^2)}{n}}} \tag{5}$$

where $A_{Y(t)}\ N(0,1)$. Similarly, the variable of interest for the process dispersion using $D^{(2)}_{Y(t)}$ statistic is defined as:

$$B_{Y(t)}=\frac{D^{(2)}_{Y(t)}-0}{\sqrt{1-\rho^{*2}}} \tag{6}$$

where $B_{Y(t)}\ N(0,1)$.

2.5. EWMA$^{(1)}_{AIB}$2.5 and EWMA$^{(2)}_{AIB}$ control charts

The EWMA$^{(1)}_{\text{AIB}}$ control chart is proposed by Abbas et al. (2014) based on $A_{Y(t)}$ statistic to monitor a process location. The EWMA$^{(1)}_{\text{AIB}}$ control chart can be constructed as follows:

$$A^{(1)}_{Y(t)}=\lambda_1 A_{Y(t)}+(1-\lambda_1)A^{(1)}_{Y(t-1)} \tag{7}$$

where $A^{(1)}_{Y(0)}=0$ is the initial value and $\lambda_1\in[0,1]$ is a smoothing parameter of the EWMA$^{(1)}_{\text{AIB}}$ control chart. The time-varying lower control limit (LCL) and upper control limit (ULC) of the EWMA$^{(1)}_{\text{AIB}}$ control chart are presented as follows:

$$\left.\begin{aligned} LCL_{\text{EWMA}^{(1)}_{\text{AIB}}(t)}&=-L_{\text{EWMA}^{(1)}_{\text{AIB}}}\sqrt{\frac{\lambda_1}{2-\lambda_1}\left(1-(1-\lambda_1)^{2t}\right)}\\ UCL_{\text{EWMA}^{(1)}_{\text{AIB}}(t)}&=L_{\text{EWMA}^{(1)}_{\text{AIB}}}\sqrt{\frac{\lambda_1}{2-\lambda_1}\left(1-(1-\lambda_1)^{2t}\right)}\end{aligned}\right\} \tag{8}$$

respectively. The $L_{\text{EWMA}^{(1)}_{\text{AIB}}}$ is a control limit coefficient that depends on λ_1 (smoothing parameter) and pre-defined false alarm rate. If the $A^{(1)}_{Y(t)}>UCL_{\text{EWMA}^{(1)}_{\text{AIB}}(t)}$ or $A^{(1)}_{Y(t)}<LCL_{\text{EWMA}^{(1)}_{\text{AIB}}(t)}$, the process is out-of-control; otherwise, in-control. Similarly, the EWMA$^{(2)}_{\text{AIB}}$ control chart is based on auxiliary information to monitor the process dispersion that has been designed by Haq (2017a). The EWMA$^{(2)}_{\text{AIB}}$ control chart uses the $B_{Y(t)}$ statistic as input to monitor the changes in a process dispersion. The plotting statistic is defined as follows:

$$B^{(2)}_{Y(t)}=\lambda_1 B_{Y(t)}+(1-\lambda_1)B^{(2)}_{Y(t-1)} \tag{9}$$

where $B^{(2)}_{Y(0)}=0$ is the initial value and $\lambda_1\in[0,1]$ is a smoothing parameter of the EWMA$^{(2)}_{\text{AIB}}$ control chart. The time-varying LCL and UCL of the EWMA$^{(2)}_{\text{AIB}}$ control chart can be offered as given below:

$$\left.\begin{aligned} LCL_{\text{EWMA}^{(2)}_{\text{AIB}}(t)}&=-L_{EWMA^{(2)}_{AIB}}\sqrt{\frac{\lambda_1}{2-\lambda_1}\left(1-(1-\lambda_1)^{2t}\right)}\\ UCL_{\text{EWMA}^{(2)}_{\text{AIB}}(t)}&=L_{EWMA^{(2)}_{AIB}}\sqrt{\frac{\lambda_1}{2-\lambda_1}\left(1-(1-\lambda_1)^{2t}\right)}\end{aligned}\right\} \tag{10}$$

where $L_{EWMA^{(2)}_{AIB}}$ is the control limit coefficient depends on λ_1 smoothing parameter and pre false alarm rate. If $B^{(2)}_{Y,t}>UCL_{\text{EWMA}^{(2)}_{\text{AIB}}(t)}$or $B^{(2)}_{Y,t}<LCL_{\text{EWMA}^{(2)}_{\text{AIB}}(t)}$, the process is out-of-control; otherwise, in-control.

2.6. CMEC control chart for simultaneous monitoring of process parameters

Zaman, Riaz, et al. (2016) proposed the CMEC control chart for the simultaneous monitoring of the process parameters. The CMEC control chart can be presented as follows:

$$\left.\begin{aligned} CMECC^{+}_{(t)}&=max\left[0,U^{*}_{(t)}-K_{(t)}+CMECC^{+}_{(t-1)}\right]\\ CMECC^{-}_{(t)}&=min\left[0,U^{*}_{(t)}+K_{(t)}+CMECC^{-}_{(t-1)}\right]\end{aligned}\right\} \tag{11}$$

$$\left.\begin{aligned} CMECD^{+}_{(t)}&=max\left[0,V^{*}_{(t)}-K_{(t)}+CMECD^{+}_{(t-1)}\right]\\ CMECD^{-}_{(t)}&=min\left[0,V^{*}_{(t)}+K_{(t)}+CMECD^{-}_{(t-1)}\right]\end{aligned}\right\} \tag{12}$$

where $U^{*}_{(t)}=\lambda U_{Y(t)}+(1-\lambda)U^{*}_{(t-1)},\lambda\in[0,1],V^{*}_{(t)}=\lambda V_{Y(t)}+(1-\lambda)V^{*}_{(t-1)}$, $\lambda\in[0,1]$. The $U^{*}_{(t)}$and $V^{*}_{(t)}$ are the plotting statistics of the EWMA control chart to monitor the process location and process dispersion, respectively. The $U^{*}_{(0)}=0$ and $V^{*}_{(0)}=0$ are initial values. Also, $K_{(t)}=k^{*}\sqrt{Var\left(V^{*}_{(t)}\right)}=k^{*}\sqrt{Var(U^{*}_{(t)})}=k^{*}\sqrt{\frac{\lambda}{2-\lambda}(1-(1-\lambda)^{2(t)})}$, and k is a constant coefficient. The $CMECC^{\pm}_{(t)}$ and $CMECD^{\pm}_{(t)}$ are the plotting statistics of the process location and dispersion, respectively. Plot $CMECC^{\pm}_{(t)}$ and $CMECD^{\pm}_{(t)}$ statistics against$\pm H_{(t)}$, if $(CMECC^{+}_{(t)}$ or $CMECD^{+}_{(t)})>H_{(t)}$ or $(CMECC^{-}_{(t)}$ or $CMECD^{-}_{(t)})<-H_{(t)}$, the process is out-of-control; otherwise, in-control. The $\pm H_{(t)}$ are the control limits of the CMEC control chart, and $H_{(t)}$ is defined as $H_{(t)}=h^{*}\sqrt{Var(U^{*}_{(t)})}$, where h is chosen to adjust the pre-specified in-control ARL.

Table 1
Presentation of symbols and directions of an out-of-control signal for diagnostic ability.

			$Q^+_{(t)} > H_{1(t)}$ or $Q^-_{(t)} < -H_{1(t)}$ $B^{(1)}_{Y(t)} > 0$	$Q^+_{(t)} < H_{1(t)}$ or $Q^-_{(t)} > -H_{1(t)}$ $B^{(1)}_{Y(t)} < 0$
			$V+$	$V-$
$P^+_{(t)} > H_{1(t)}$ or $P^-_{(t)} < -H_{1(t)}$	$A^{(1)}_{Y(t)} > 0$	$m+$	$++$	$+-$
$P^+_{(t)} < H_{1(t)}$ or $P^-_{(t)} > -H_{1(t)}$	$A^{(1)}_{Y(t)} < 0$	$m-$	$-+$	$--$

3. Proposed methods

This section provides the methodology of the proposed CMEC$_{AIB}$ control chart to monitor the process parameters simultaneously. Section 3.1 contains the design structure of the proposed CMEC$_{AIB}$ control chart. Likewise, Section 3.2 introduces the construction procedure of the CMEC$_{AIB}$ control chart. Finally, the mathematical proves that some existing control charts such as the classical CUSUM, CMEC, and Max-CUSUM are special cases of the proposed CMEC$_{AIB}$ control chart is given in Section 3.3.

3.1. Proposed CMEC$_{AIB}$ control chart

The EWMA$^{(1)}_{AIB}$ and EWMA$^{(2)}_{AIB}$ control charts plotting $A^{(1)}_{Y(t)}$ and $B^{(2)}_{Y(t)}$ statistics, respectively, are used as input for the classical CUSUM control charts to design the proposed CMEC$_{AIB}$control chart. The plotting statistics and control limits of the proposed CMEC$_{AIB}$ control chart are defined as follows:

$$\left.\begin{aligned} P^+_{(t)} &= max\left(0, A^{(1)}_{Y(t)} - K_{1(t)} + P^+_{(t-1)}\right) \\ P^-_{(t)} &= min\left(0, A^{(1)}_{Y(t)} + K_{1(t)} + P^-_{(t-1)}\right) \end{aligned}\right\} \quad (13)$$

$$\left.\begin{aligned} Q^+_{(t)} &= max\left(0, B^{(2)}_{Y(t)} - K_{1(t)} + Q^+_{(t-1)}\right) \\ Q^-_{(t)} &= min\left(0, B^{(2)}_{Y(t)} + K_{1(t)} + Q^-_{(t-1)}\right) \end{aligned}\right\} \quad (14)$$

where $P^{\pm}_{(0)} = Q^{\pm}_{(0)} = 0$. As $A^{(1)}_{Y(t)}$ and $B^{(2)}_{Y(t)}$ are the EWMA$^{(1)}_{AIB}$ and EWMA$^{(2)}_{AIB}$ statistics based on estimators $A_{Y(t)}$ $N(0,1)$ and $B_{Y(t)}$ $N(0,1)$, respectively. So, $K_{1(t)} = k_1 * \sqrt{var(A^{(1)}_{Y(t)})}$ or $K_{1(t)} = k_1 * \sqrt{var(B^{(2)}_{Y(t)})}$. Hence, $K_{1(t)} = k_1 * \sqrt{\frac{\lambda_1}{2-\lambda_1}\left\{1-(1-\lambda_1)^{2(t)}\right\}}$, where, k_1 is a CMEC$_{AIB}$control chart coefficient. The $P^{\pm}_{(t)}$, and $Q^{\pm}_{(t)}$ are plotting statistics of the proposed CMEC$_{AIB}$ control chart for process location and dispersion, respectively. If $(P^+_{(t)}$ or $Q^+_{(t)}) > H_{1(t)}$ or $(P^-_{(t)}$ or $Q^-_{(t)}) < -H_{1(t)}$, the process is OC. Here $\pm H_{1(t)} = \pm h_1 * \sqrt{\frac{\lambda_1}{2-\lambda_1}\left\{1-(1-\lambda_1)^{2(t)}\right\}}$ are control limits and h_1 is known as the control limit coefficient.

3.2. Step-by-step computational algorithm of proposed CMEC$_{AIB}$ control chart

The purpose of this subsection is to provide a step-by-step understanding for users and practitioners how to implement the proposed control chart using monte Carlo simulation for in-control and shifted processes. In more general, the algorithm provide the detail about the following;

(1) To choose the suitable value of h_1.So that the desired in-control ARL$_0$ is obtained (see steps (i)-(x)).
(2) To obtain pre-specified in-control ARL$_0$ if h_1 is given in advance (see steps (ii)-(x)).
(3) To obtain out-of-control ARL$_1$, if h_1 and ARL$_0$ are given in advance using steps (ii)-(x) and (xi).

Generally, these steps can further catgorized as following.
(a). Computation of purposed CMEC$_{AIB}$ statistic

(i) Generate random observations from $(Y_{(it)}, X_{(it)})$ $N(\mu_Y, \mu_X, \sigma_Y, \sigma_X, \rho)$ $(t = 1, 2, 3, \cdots)$, however, when parameters are unknown, then sample estimates are used for the generation of the random sample (Chen & Cheng, 1998; Lee, Aslam, Shakeel, Lee, & Jun, 2015; Mughal, Azam, & Aslam, 2018).
(ii) Calculate the $A_{Y(t)}$ and $B_{Y(t)}$ statistics from Eqs. (5) and (6), respectively, using required estimators.
(iii) Calculate the $A^{(1)}_{Y(t)}$ and $B^{(2)}_{Y(t)}$ statistics of the EWMA$^{(1)}_{AIB}$ and EWMA$^{(2)}_{AIB}$ control charts from Eqs. (7) and (9) at fix value of λ_1, respectively.
(iv) Use the $A^{(1)}_{Y(t)}$ and $B^{(2)}_{Y(t)}$ statistics as inputs in Eqs. (13) and (14) to obtain $P^{\pm}_{(t)}$and $Q^{\pm}_{(t)}$ statistics, respectively.

(b). Setting up control limits

(v) Choose h_1 along with other desired parameters $(\lambda_1, k_1, \rho_{YX})$ for desired ARL$_0$.
(vi) Compute $H_{1(t)}$ based on h_1 and λ_1.
(vii) Plot $P^{\pm}_{(t)}$and $Q^{\pm}_{(t)}$ statistics simultaneously against the $H_{1(t)}$ control limit.
(viii) If $(P^+_{(t)}$ or $Q^+_{(t)}) > H_{1(t)}$ or $(P^-_t$ or $Q^-_t) < -H_{1(t)}$, record sequence order, which is called run length (RL).
(ix) Repeat from steps (i)-(viii) 10^5 times and record RLs.
(x) Calculate the average of 10^5 RL, which is ARL$_0$.

If it is desired pre-specified in-control ARL; otherwise, adjust the h_1 accordingly and repeat steps from (i)-(ix) until will not get desired ARL$_0$.
(c). Evaluating the Out-of-control ARL
For shifted process, introduce shifts in the process mean and process standard deviation following (Mughal et al., 2018).

(xi) For out-of-control ARL values, considered$(Y_{(it)}, X_{(it)})$ $N(\mu_{Y+\delta_\mu\sigma_Y}, \mu_X, \delta_\gamma\sigma_Y, \sigma_X, \rho)$ and repeat steps (ii)-(x) similarly as described above.

3.3. Symbols representing source and direction of out-of-control signals

In SPC, the simultaneous monitoring of process parameters required the source and direction of out-of-control points. The monitoring of the control chart in this way is known as diagnostic ability. The source may be location or dispersion, while the direction may be decreasing or increasing. Table 1 provides the symbols related to the source and direction of an out-of-control signal. If $P^+_{(t)} > H_{1(t)}$ or $P^-_{(t)} < -H_{1(t)}$ with condition $A^{(1)}_{Y(t)} > 0$ then plot $''m+''$ versus t for an upward shift in the process location, and then plot $''m-''$ versus t when $A^{(1)}_{Y(t)} < 0$ for the downward shift in the process location. If $Q^+_{(t)} > H_{1(t)}$ or $Q^-_{(t)} < -H_{1(t)}$ and $B^{(2)}_{Y(t)} > 0$ then plot $''V+''$ versus t for an upward shift in process dispersion, and plot $''V-''$ versus t when $B^{(2)}_{Y(t)} < 0$ for a downward shift

in process dispersion. If $P^{+}_{(t)} > H_{1(t)}$ or $P^{-}_{t} < -H_{1(t)}$ when $A^{(1)}_{Y(t)} > 0$ and $Q^{+}_{(t)} > H_{1(t)}$ or $Q^{-}_{(t)} < -H_{1(t)}$ when $B^{(2)}_{Y(t)} > 0$ then plot "++" versus t for an upward shift in the process location and process dispersion. If $P^{+}_{(t)} > H_{1(t)}$ or $P^{-}_{(t)} < -H_{1(t)}$ where $A^{(1)}_{Y(t)} > 0$ and $Q^{+}_{(t)} < H_{1(t)}$ or $Q^{-}_{(t)} > -H_{1(t)}$ where $B^{(2)}_{Y(t)} < 0$ then plot "+−" versus t for an upward shift in the process location and a downward shift in process dispersion. If $P^{+}_{(t)} < H_{1(t)}$ or $P^{-}_{(t)} > -H_{1(t)}$ for $A^{(1)}_{Y(t)} < 0$ and$Q^{+}_{(t)} > H_{1(t)}$ or $Q^{-}_{(t)} < -H_{1(t)}$ for $B^{(2)}_{Y(t)} > 0$ then plot "−+" versus t for the downward shift in the process location and an upward shift in process dispersion. If $P^{+}_{(t)} < H_{1(t)}$ or $P^{-}_{(t)} > -H_{1(t)}$ when $A^{(1)}_{Y(t)} < 0$ and $Q^{+}_{(t)} < H_{1(t)}$ or $Q^{-}_{(t)} > -H_{1(t)}$ when $B^{(2)}_{Y(t)} < 0$ then plot "−−" versus t for the downward shift in the process location and a downward shift in the process dispersion.

3.4. Special cases of proposed CMEC$_{AIB}$ control chart

Some existing control charts such as the classical CUSUM, CMEC, and MaxCUSUM are special cases of the proposed CMEC$_{AIB}$ control chart. More details are given in the consequent subsections.

3.4.1. CUSUM is a special case of CMEC$_{AIB}$ control chart

The CUSUM control charts based on auxiliary information to monitor the process location and dispersion are the special cases of the proposed CMEC$_{AIB}$ control chart. Let assumed$\lambda_1 = 1$, and put it in Eqs. (7) and (9) to obtain the following statistics:

$$\left.\begin{array}{l} A^{(1)}_{Y(t)} = A_{Y(t)} \\ B^{(2)}_{Y(t)} = B_{Y(t)} \end{array}\right\} \tag{15}$$

So, based on Eq. (15), the CMEC$_{AIB}$ control chart coverages to the CUSUM control charts based on auxiliary information to monitor the process location and dispersion with the following statistics

$$\left.\begin{array}{l} P^{+}_{(t)} = max\left(0, A_{Y(t)} - K + P^{+}_{(t-1)}\right) \\ P^{-}_{(t)} = min\left(0, A_{Y(t)} + K + P^{-}_{(t-1)}\right) \end{array}\right\} \tag{16}$$

$$\left.\begin{array}{l} Q^{+}_{(t)} = max\left(0, B_{Y(t)} - k + Q^{+}_{(t-1)}\right) \\ Q^{-}_{(t)} = min\left(0, B_{Y(t)} + K + Q^{-}_{(t-1)}\right) \end{array}\right\} \tag{17}$$

Hence the proposed CMEC$_{AIB}$ control chart followed by $\lambda_1 = 1$ became the CUSUM control chart based on auxiliary information to monitor the process location and dispersion simultaneously.

3.4.2. CMEC is a special case of CMEC$_{AIB}$ control chart

The CMEC control chart is the special of the proposed CMEC$_{AIB}$ control chart at $\rho = \rho^{*} = 0$. So, the Eqs. (3) and (4) will be reduced in the below expressions:

$$D^{*(1)}_{Y(t)} = \overline{Y} \tag{18}$$

$$D^{*(2)}_{Y(t)} = V_{Y(t)} \tag{19}$$

respectively. The Eqs. (5) and (6) are based on Eqs. (3) and (4), respectively. Consequences, the new forms of Eqs. (5) and (6) based on Eqs. (19) and (20), respectively along $\rho = \rho^{*} = 0$ can be defined as follows:

$$A^{*}_{Y(t)} = \frac{D^{*(1)}_{Y(t)} - \mu_Y}{\sqrt{\frac{\sigma^2_Y}{n}}} \tag{20}$$

$$B^{*}_{Y(t)} = D^{*(2)}_{Y(t)} \tag{21}$$

Similarly, the Eqs. (8) and (9) based on Eqs. (21) and (22) will be changed as follows:

$$A^{*(1)}_{Y(t)} = \lambda_1 A^{*}_{Y(t)} + (1-\lambda_1) A^{*(1)}_{Y(t-1)} \tag{22}$$

$$B^{*(2)}_{Y(t)} = \lambda_1 B^{*}_{Y(t)} + (1-\lambda_1) B^{*(2)}_{Y(t-1)} \tag{23}$$

respectively. Consequently, the Eqs. (11) and (12) based on Eqs. (23) and (24) can be redefined as follows:

$$\left.\begin{array}{l} P^{+}_{*(t)} = max\left(0, A^{*(1)}_{Y(t)} - K_{1(t)} + P^{+}_{*(t-1)}\right) \\ P^{-}_{*(t)} = min\left(0, A^{*(1)}_{Y(t)} + K_{1(t)} + P^{-}_{*(t-1)}\right) \end{array}\right\} \tag{24}$$

$$\left.\begin{array}{l} Q^{+}_{*(t)} = max\left(0, B^{*(2)}_{Y(t)} - K_{1(t)} + Q^{+}_{*(t-1)}\right) \\ Q^{-}_{*(t)} = min\left(0, B^{*(2)}_{Y(t)} + K_{1(t)} + Q^{-}_{*(t-1)}\right) \end{array}\right\} \tag{25}$$

respectively. The Eqs. (25) and (26) have the same function as Eqs. (11) and (12), respectively, except their notations. Therefore, it can be concluded, the proposed CMEC$_{AIB}$ control chart at $\rho = \rho^{*} = 0$ coverages to the CMEC control chart, which is designed to monitor the process location and dispersion jointly.

3.4.3. MaxCUSUM is a special case of CMEC$_{AIB}$ control chart

The proposed CMEC$_{AIB}$ control chart converges to the MaxCUSUM control chart to monitor process location and dispersion simultaneously if $\lambda_1 = 1$ and $\rho = \rho^{*} = 0$. Let $\lambda_1 = 1$ for Eqs. (23) and (24). So, based on this relation, the Eqs. (24) and (25) will have the following forms

$$\left.\begin{array}{l} P^{+}_{*(t)} = max\left(0, A^{*}_{Y(t)} - K_{1(t)} + P^{+}_{*(t-1)}\right) \\ P^{-}_{*(t)} = min\left(0, A^{*}_{Y(t)} + K_{1(t)} + P^{-}_{*(t-1)}\right) \end{array}\right\} \tag{26}$$

$$\left.\begin{array}{l} Q^{+}_{*(t)} = max\left(0, B^{*}_{Y(t)} - K_{1(t)} + Q^{+}_{*(t-1)}\right) \\ Q^{-}_{*(t)} = min\left(0, B^{*}_{Y(t)} + K_{1(t)} + Q^{-}_{*(t-1)}\right) \end{array}\right\} \tag{27}$$

respectively. The Eqs. (26) and (27) are the plotting statistics of the MaxCUSUM control chart designed by Thaga (2004). Hence the proposed CMEC$_{AIB}$ control chart at $\rho = \rho^{*} = 0$ and $\lambda_1 = 1$ is the MaxCUSUM control chart.

4. Performance measure

This section introduces the performance measure for comparison purposes. Section 4.1 contains the detail of the Monte Carlo simulation, and the definition of the ARL is provided in Section 4.2. The role of parameters choices on the performance of the CMEC$_{AIB}$ control chart is given in Section 4.3.

4.1. Simulation of proposed CMEC$_{AIB}$ control chart

The values of $Y_{(it)}$and$X_{(it)}$ for $i = 1, 2, ..., n$ $(t > 1)$ are generated from a bivariate normal distribution at the various choices of parameters. The two-sided shift is considered. The shift is reflected in the process location as: μ_Y to $\mu_Y + \delta_\mu \sigma_Y$, where $\delta_\mu = 0.00, 0.25, 0.50, 1.00, 2.00$, while the shift in the process dispersion as: σ_Y to $\delta_\gamma \sigma_Y$, where $\delta_\gamma = 0.25, 0.5, 0.75, 1.00, 1.25, 1.50, 2.00$. The Monte Carlo simulation method is used as a computational technique for the numerical results. Monte Carlo simulations with 10^5 iterations are carried out for each displacement of δ_μ and δ_γ. An algorithm is designed in R software, and detail of this algorithm is provided in Section 3.2.

4.2. Average run length measure

The ARL measure is the most famous and commonly used performance measure to judge a control chart performance at a shift. The ARL is categorized as in-control ARL, denotedas ARL$_0$and out-of-control ARL represented as ARL$_1$. If a process is functioning in an in-control state, the

Table 2
ARLs properties of proposedCMEC$_{AIB}$ control chart when ARL$_0$ = 250 and λ_1 = 0.10.

δ_μ	0.00	0.25	0.50	1.0	2.00	0.00	0.25	0.50	1.0	2.00	0.00	0.25	0.50	1.0	2.00
						$k_1 = 0.5$									
δ_γ	$\rho = 0, h_1 = 35.98$					$\rho = 0.25, h_1 = 35.99$					$\rho = 0.50, h_1 = 35.98$				
0.25	9.08	9.09	9.09	9.09	6.01	9.07	9.08	9.08	9.05	6.00	8.92	8.92	8.92	8.77	5.83
0.50	13.29	13.31	13.26	10.07	6.14	13.28	13.28	13.24	9.85	6.04	13.04	13.04	12.89	9.11	5.80
0.75	24.61	22.99	16.55	10.06	6.17	24.59	22.88	16.16	9.84	6.06	23.91	21.84	14.92	9.11	5.68
1.00	253.56	31.06	16.94	10.14	6.26	252.61	30.21	16.54	9.88	6.13	251.05	27.13	15.17	9.15	5.70
1.25	26.26	23.42	16.39	10.12	6.22	26.51	23.21	16.20	9.88	6.10	25.91	22.15	15.04	9.14	5.66
1.50	15.72	15.38	14.13	10.15	6.29	15.70	15.34	14.03	9.93	6.16	15.40	15.02	13.44	9.21	5.70
2.00	9.78	9.73	9.64	8.91	6.31	9.78	9.72	9.62	8.82	6.19	8.92	8.92	8.92	8.77	5.83
	$\rho = 0.75, h_1 = 35.95$					$\rho = 0.90, h_1 = 35.95$					$\rho = 0.95, h_1 = 35.90$				
0.25	8.06	8.02	8.06	7.43	4.82	6.49	6.44	6.46	5.65	3.53	5.28	5.27	5.22	4.50	2.95
0.50	11.69	11.74	11.30	7.53	4.84	9.40	9.36	8.72	5.68	3.61	7.66	7.64	6.99	4.50	2.99
0.75	21.20	18.84	12.29	7.53	4.85	16.49	14.24	9.11	5.72	3.61	13.22	11.39	7.24	4.50	3.00
1.00	253.50	21.29	12.34	7.57	4.80	251.38	15.15	9.16	5.69	3.60	253.01	11.77	7.29	4.55	3.00
1.25	23.11	18.77	12.37	7.55	4.73	17.72	14.32	9.20	5.66	3.56	14.06	11.48	7.30	4.52	2.99
1.50	13.75	13.40	11.50	7.68	4.71	10.84	10.63	8.93	5.66	3.54	8.79	8.62	7.19	4.53	2.96
2.00	8.62	8.59	8.43	7.24	4.72	6.91	6.89	6.77	5.67	3.53	5.66	5.62	5.49	4.49	2.89
						$k_1 = 1.5$									
	$\rho = 0.0, h_1 = 6.87$					$\rho = 0.25, h_1 = 6.865$					$\rho = 0.50, h_1 = 6.88$				
0.25	4.00	3.95	3.97	3.94	2.36	3.99	3.94	3.95	3.90	2.18	3.90	3.88	3.88	3.76	2.02
0.50	6.69	6.67	6.61	4.51	2.39	6.68	6.60	6.56	4.37	2.29	6.54	6.44	6.29	3.98	2.03
0.75	16.97	14.62	8.98	4.63	2.46	16.86	14.34	8.60	4.48	2.38	16.44	13.49	7.71	4.06	2.09
1.00	253.90	23.62	9.71	4.64	2.50	252.99	23.26	9.25	4.40	2.39	251.66	19.46	8.19	4.08	2.17
1.25	17.93	14.04	8.92	4.71	2.49	18.19	13.86	8.68	4.50	2.43	17.57	13.02	7.79	4.05	2.19
1.50	8.42	7.77	6.94	4.51	2.50	8.38	7.70	6.87	4.43	2.44	8.14	7.53	6.39	4.11	2.21
2.00	4.39	4.43	4.22	3.68	2.52	4.39	4.39	4.17	3.63	2.48	4.30	4.28	4.07	3.48	2.29
	$\rho = 0.75, h_1 = 6.866$					$\rho = 0.90, h_1 = 6.88$					$\rho = 0.95, h_1 = 6.92$				
0.25	3.41	3.43	3.39	3.00	1.96	2.63	2.64	2.56	2.14	1.11	2.07	2.06	2.03	1.71	1.01
0.50	5.67	5.58	5.20	3.16	1.99	4.25	4.16	3.69	2.17	1.06	3.27	3.19	2.80	1.78	1.00
0.75	13.52	10.55	6.00	3.15	1.98	9.14	7.13	4.05	2.14	1.02	6.76	5.33	3.03	1.88	1.00
1.00	252.41	13.43	6.11	3.18	1.94	253.13	8.27	4.08	2.16	1.03	253.50	5.83	3.04	1.92	1.00
1.25	14.61	10.33	6.11	3.23	1.91	10.14	7.16	4.07	2.23	1.08	7.28	5.27	3.09	1.81	1.00
1.50	6.98	6.46	5.30	3.24	1.88	5.09	4.74	3.71	2.29	1.15	3.88	3.63	2.87	1.77	1.00
2.00	3.75	3.73	3.51	2.87	1.86	2.83	2.81	2.62	2.11	1.25	2.22	2.19	2.06	1.61	1.05
						$k_1 = 2.0$									
	$\rho = 0.0, h_1 = 2.265$					$\rho = 0.25, h_1 = 2.27$					$\rho = 0.50, h_1 = 2.31$				
0.25	2.61	2.61	2.58	2.58	1.21	2.61	2.60	2.58	2.56	1.13	2.53	2.55	2.55	2.32	1.04
0.50	5.07	5.01	4.97	3.05	1.32	5.07	4.99	4.88	2.97	1.24	5.00	4.92	4.58	2.62	1.07
0.75	16.42	13.51	7.13	3.16	1.40	16.29	13.23	6.94	3.05	1.34	15.66	12.10	6.06	2.65	1.13
1.00	252.99	23.33	7.94	3.22	1.45	251.66	22.13	7.71	3.10	1.38	249.05	18.52	6.47	2.76	1.20
1.25	16.22	11.85	6.99	3.17	1.45	15.97	11.76	6.76	3.08	1.39	15.76	10.87	6.08	2.80	1.26
1.50	6.41	5.96	4.81	3.06	1.47	6.41	5.95	4.74	2.97	1.43	6.27	5.70	4.50	2.71	1.29
2.00	2.92	2.83	2.72	2.24	1.44	2.90	2.81	2.70	2.19	1.41	2.79	2.75	2.53	2.12	1.32
	$\rho = 0.75, h_1 = 2.35$					$\rho = 0.90, h_1 = 2.3195$					$\rho = 0.95, h_1 = 2.3195$				
0.25	2.20	2.20	2.18	1.90	1.00	1.55	1.53	1.46	1.16	1.00	1.10	1.10	1.07	1.01	1.00
0.50	4.20	4.10	3.64	1.98	1.00	2.86	2.77	2.29	1.24	1.00	2.06	1.97	1.63	1.04	1.00
0.75	12.52	8.92	4.45	2.04	1.00	7.63	5.49	2.67	1.17	1.00	5.12	3.69	1.90	1.00	1.00
1.00	249.47	12.16	4.56	2.01	1.01	252.88	6.63	2.72	1.23	1.00	251.81	4.24	1.93	1.00	1.00
1.25	12.92	8.52	4.41	2.05	1.04	8.15	5.26	2.75	1.27	1.00	5.62	3.60	1.91	1.03	1.00
1.50	5.15	4.76	3.57	2.03	1.07	3.46	3.17	2.32	1.34	1.00	2.43	2.24	1.71	1.08	1.00
2.00	2.40	2.31	2.16	1.70	1.13	1.64	1.61	1.56	1.25	1.01	1.26	1.27	1.20	1.07	1.00
						$k_1 = 2.5$									
	$\rho = 0.0, h_1 = 0.408$					$\rho = 0.25, h_1 = 0.44$					$\rho = 0.50, h_1 = 0.44$				
0.25	1.82	1.83	1.83	1.77	1.00	1.83	1.83	1.86	1.75	1.00	1.77	1.77	1.76	1.55	1.00
0.50	4.21	4.22	3.99	2.16	1.00	4.30	4.28	3.95	2.13	1.00	4.06	4.10	3.66	1.79	1.00
0.75	16.54	13.49	6.25	2.23	1.02	16.80	13.24	6.05	2.17	1.01	15.61	11.80	5.22	1.86	1.00
1.00	250.83	21.96	6.69	2.32	1.07	249.18	21.89	6.46	2.21	1.04	251.59	17.76	5.51	1.92	1.01
1.25	13.93	10.01	5.68	2.29	1.11	14.21	9.99	5.65	2.27	1.09	13.61	9.25	4.66	1.94	1.03
1.50	4.74	4.43	3.65	2.07	1.17	4.79	4.61	3.66	2.04	1.13	4.58	4.27	3.42	1.84	1.07
2.00	2.01	1.92	1.87	1.55	1.14	2.01	1.93	1.88	1.58	1.13	1.92	1.90	1.77	1.49	1.09
	$\rho = 0.75, h_1 = 0.44$					$\rho = 0.90, h_1 = 0.45$					$\rho = 0.95, h_1 = 0.435$				
0.25	1.42	1.44	1.39	1.14	1.00	1.05	1.05	1.03	1.00	1.00	1.01	1.01	1.00	1.00	1.00
0.50	3.22	3.25	2.61	1.28	1.00	1.97	1.89	1.52	1.03	1.00	1.31	1.25	1.10	1.00	1.00
0.75	12.08	8.23	3.40	1.31	1.00	6.81	4.48	1.90	1.01	1.00	4.02	2.63	1.30	1.00	1.00
1.00	250.66	10.95	3.59	1.39	1.00	251.59	5.40	1.94	1.02	1.00	250.66	3.22	1.25	1.00	1.00
1.25	11.17	6.81	3.38	1.43	1.00	6.82	4.04	1.96	1.06	1.00	4.32	2.50	1.33	1.00	1.00
1.50	3.86	3.33	2.49	1.46	1.01	2.49	2.15	1.60	1.09	1.00	1.67	1.53	1.24	1.01	1.00
2.00	1.61	1.61	1.49	1.26	1.02	1.25	1.24	1.16	1.07	1.00	1.09	1.08	1.05	1.02	1.00

Table 3
ARLs properties of proposed $CMEC_{AIB}$ control chart when $ARL_0 = 250$ and.$\lambda_1 = 0.20$

δ_μ	0.00	0.25	0.50	1.0	2.00	0.00	0.25	0.50	1.0	2.00	0.00	0.25	0.50	1.0	2.00
						$k_1 = 0.5$									
δ_γ	$\rho = 0, h_1 = 23.09$					$\rho = 0.25, h_1 = 23.09$					$\rho = 0.50, h_1 = 23.10$				
0.25	6.74	6.72	6.73	6.73	4.83	6.74	6.73	6.73	6.71	4.46	6.63	6.58	6.60	6.42	4.02
0.50	10.19	10.19	10.13	7.56	4.77	10.01	10.07	10.00	7.35	4.45	9.83	9.84	9.69	6.81	4.04
0.75	21.04	19.49	13.20	7.67	4.78	20.94	19.16	12.92	7.51	4.65	19.93	17.59	11.61	6.77	4.09
1.00	249.04	27.10	13.29	7.64	4.63	249.33	22.88	11.80	6.78	4.16	249.33	22.88	11.80	6.78	4.16
1.25	22.67	19.41	13.21	7.76	4.69	22.92	19.17	12.92	7.52	4.60	21.87	17.67	11.55	6.84	4.17
1.50	12.04	11.82	10.65	7.54	4.64	12.00	11.65	10.56	7.41	4.54	11.83	11.45	10.08	6.81	4.17
2.00	7.29	7.19	7.16	6.53	4.65	7.27	7.14	7.11	6.43	4.54	7.11	7.04	6.95	6.18	4.23
	$\rho = 0.75, h_1 = 23.0$					$\rho = 0.90, h_1 = 22.98$					$\rho = 0.95, h_1 = 23.0$				
0.25	5.99	5.95	5.96	5.49	3.40	4.83	4.84	4.80	4.16	2.72	3.98	3.98	3.95	3.32	2.03
0.50	8.82	8.79	8.44	5.54	3.43	7.00	6.95	6.47	4.16	2.78	5.69	5.62	5.14	3.30	2.00
0.75	17.31	14.75	9.25	5.63	3.39	12.82	10.83	6.82	4.10	2.83	10.03	8.54	5.36	3.22	2.00
1.00	251.55	17.18	9.40	5.63	3.40	253.78	11.75	6.79	4.15	2.80	253.13	9.02	5.34	3.18	2.00
1.25	18.94	14.86	9.34	5.58	3.46	13.95	10.88	6.89	4.21	2.74	10.82	8.50	5.40	3.29	2.00
1.50	10.49	10.16	8.65	5.61	3.45	8.16	7.88	6.62	4.26	2.67	6.53	6.33	5.29	3.33	2.02
2.00	6.41	6.42	6.23	5.36	3.48	5.11	5.09	4.89	4.12	2.63	4.17	4.17	3.97	3.30	2.10
						$k_1 = 1.5$									
	$\rho = 0.0, h_1 = 4.98$					$\rho = 0.25, h_1 = 4.99$					$\rho = 0.50, h_1 = 4.99$				
0.25	3.27	3.31	3.29	3.26	2.00	3.26	3.29	3.27	3.20	2.00	3.22	3.21	3.21	3.04	2.00
0.50	5.79	5.67	5.68	3.78	2.00	5.75	5.68	5.57	3.67	2.00	5.61	5.53	5.32	3.31	1.99
0.75	18.11	14.88	7.99	3.89	2.05	17.89	14.56	7.67	3.78	2.02	17.20	13.26	6.67	3.39	1.96
1.00	250.11	26.05	8.78	3.90	2.06	249.05	20.39	7.30	3.41	1.91	249.05	20.39	7.30	3.41	1.91
1.25	17.43	13.07	7.77	3.92	2.10	17.48	13.05	7.56	3.76	2.04	16.92	12.04	6.90	3.41	1.87
1.50	7.39	6.73	5.84	3.81	2.10	7.39	6.73	5.76	3.71	2.09	7.08	6.50	5.39	3.40	1.88
2.00	3.72	3.62	3.50	2.97	2.09	3.71	3.62	3.47	2.96	2.03	3.60	3.52	3.35	2.81	1.88
	$\rho = 0.75, h_1 = 5.0$					$\rho = 0.90, h_1 = 5.02$					$\rho = 0.95, h_1 = 5.02$				
0.25	2.90	2.89	2.87	2.48	1.39	2.14	2.15	2.08	1.83	1.01	1.81	1.77	1.73	1.30	1.00
0.50	4.84	4.83	4.33	2.64	1.38	3.53	3.49	3.06	1.90	1.00	2.72	2.61	2.24	1.35	1.00
0.75	13.36	9.62	5.19	2.61	1.34	8.30	6.18	3.38	1.95	1.00	5.79	4.43	2.54	1.26	1.00
1.00	248.63	13.02	5.23	2.61	1.39	248.06	7.31	3.44	1.92	1.00	249.28	4.95	2.52	1.27	1.00
1.25	13.91	9.32	5.12	2.68	1.42	9.03	6.08	3.44	1.90	1.01	6.34	4.39	2.55	1.35	1.00
1.50	6.04	5.49	4.33	2.69	1.43	4.29	3.97	3.11	1.85	1.02	3.20	3.00	2.33	1.38	1.00
2.00	3.14	3.06	2.88	2.37	1.46	2.33	2.28	2.17	1.69	1.09	1.79	1.75	1.66	1.35	1.02
						$k_1 = 2$									
	$\rho = 0.0, h_1 = 1.985$					$\rho = 0.25, h_1 = 2.01$					$\rho = 0.50, h_1 = 2.03$				
0.25	2.42	2.46	2.44	2.41	1.03	2.42	2.46	2.44	2.40	1.03	2.38	2.37	2.37	2.16	1.01
0.50	4.91	4.82	4.72	2.92	1.17	4.91	4.82	4.68	2.81	1.11	4.76	4.71	4.37	2.44	1.02
0.75	20.20	16.08	7.52	3.02	1.26	20.24	15.64	7.19	2.93	1.21	19.39	13.67	5.98	2.54	1.09
1.00	251.13	28.73	7.97	2.97	1.34	249.33	21.11	6.54	2.55	1.12	249.33	21.11	6.54	2.55	1.12
1.25	17.21	12.46	6.72	2.99	1.35	16.87	12.23	6.77	2.84	1.33	16.50	11.12	5.89	2.66	1.17
1.50	6.18	5.63	4.56	2.86	1.40	6.18	5.62	4.54	2.81	1.35	6.04	5.28	4.20	2.48	1.23
2.00	2.67	2.67	2.50	2.10	1.35	2.67	2.60	2.50	2.09	1.33	2.57	2.54	2.36	1.92	1.27
	$\rho = 0.75, h_1 = 2.03$					$\rho = 0.90, h_1 = 2.03$					$\rho = 0.95, h_1 = 2.04$				
0.25	2.06	2.10	2.04	1.75	1.00	1.41	1.38	1.33	1.10	1.00	1.06	1.06	1.05	1.00	1.00
0.50	4.00	3.89	3.40	1.85	1.00	2.68	2.57	2.10	1.17	1.00	1.91	1.83	1.50	1.03	1.00
0.75	14.23	9.32	4.25	1.86	1.00	7.80	5.29	2.51	1.11	1.00	4.89	3.48	1.78	1.00	1.00
1.00	252.41	13.21	4.32	1.88	1.01	253.42	6.53	2.58	1.15	1.00	251.55	4.10	1.75	1.00	1.00
1.25	13.18	8.33	4.17	1.91	1.02	8.14	5.03	2.60	1.24	1.00	5.43	3.30	1.78	1.02	1.00
1.50	4.95	4.45	3.42	1.87	1.05	3.27	2.93	2.17	1.30	1.00	2.29	2.05	1.63	1.06	1.00
2.00	2.20	2.12	2.04	1.58	1.09	1.54	1.53	1.46	1.20	1.01	1.20	1.22	1.16	1.06	1.00
						$k_1 = 2.5$									
	$\rho = 0.0, h_1 = 0.57$					$\rho = 0.25, h_1 = 0.59$					$\rho = 0.50, h_1 = 0.59$				
0.25	1.94	1.96	1.96	1.88	1.00	1.97	1.99	1.96	1.86	1.00	1.88	1.91	1.87	1.68	1.00
0.50	4.69	4.65	4.34	2.29	1.00	4.70	4.66	4.36	2.24	1.00	4.47	4.42	3.98	1.98	1.00
0.75	22.79	18.30	7.48	2.46	1.04	22.77	17.87	7.24	2.33	1.02	21.37	15.24	6.03	2.02	1.01
1.00	249.00	28.59	7.64	2.48	1.08	251.59	28.04	7.45	2.43	1.06	248.08	22.74	6.01	2.01	1.02
1.25	15.94	11.16	6.13	2.54	1.11	16.20	11.36	5.94	2.45	1.11	15.70	10.09	5.13	2.06	1.04
1.50	5.05	4.68	3.91	2.23	1.16	5.12	4.82	3.86	2.23	1.15	4.95	4.59	3.60	1.93	1.08
2.00	2.12	2.01	1.98	1.63	1.18	2.11	2.02	1.96	1.64	1.16	2.04	1.96	1.88	1.55	1.10
	$\rho = 0.75, h_1 = 0.58$					$\rho = 0.90, h_1 = 0.605$					$\rho = 0.95, h_1 = 0.61$				
0.25	1.52	1.55	1.49	1.20	1.00	1.07	1.07	1.05	1.00	1.00	1.01	1.01	1.00	1.00	1.00
0.50	3.46	3.45	2.80	1.35	1.00	2.13	2.03	1.66	1.04	1.00	1.41	1.32	1.15	1.00	1.00
0.75	15.27	9.62	3.73	1.34	1.00	7.82	4.88	2.02	1.02	1.00	4.49	2.92	1.36	1.00	1.00
1.00	250.62	13.04	3.81	1.42	1.00	249.50	6.08	2.07	1.03	1.00	249.50	3.51	1.32	1.00	1.00
1.25	12.41	7.64	3.48	1.44	1.00	7.60	4.36	2.06	1.06	1.00	4.68	2.69	1.39	1.00	1.00
1.50	4.07	3.54	2.66	1.51	1.01	2.61	2.27	1.72	1.11	1.00	1.78	1.61	1.27	1.02	1.00
2.00	1.68	1.69	1.54	1.31	1.03	1.27	1.27	1.20	1.08	1.00	1.10	1.09	1.06	1.02	1.00

Table 4
ARLs properties of proposed $CMEC_{AIB}$ control chart when $ARL_0 = 250$ and.$\lambda_1 = 0.50$

δ_μ	0.00	0.25	0.50	1.0	2.00	0.00	0.25	0.50	1.0	2.00	0.00	0.25	0.50	1.0	2.00
						$k_1 = 0.5$									
δ_γ	$\rho = 0, h_1 = 11.099$					$\rho = 0.25, h_1 = 11.01$					$\rho = 0.50, h_1 = 11.0$				
0.25	4.29	4.29	4.29	4.27	3.00	4.28	4.28	4.29	4.23	3.00	4.20	4.20	4.19	4.05	2.73
0.50	7.03	7.03	6.93	4.87	2.99	7.01	7.03	6.88	4.74	2.98	6.84	6.84	6.61	4.32	2.70
0.75	18.26	15.79	9.64	4.94	2.96	18.09	15.40	9.18	4.80	2.91	17.56	14.44	8.16	4.37	2.66
1.00	251.53	25.06	9.96	4.96	2.93	250.67	23.88	9.67	4.83	2.87	250.26	20.40	8.46	4.39	2.63
1.25	18.79	14.73	9.32	5.02	2.95	18.73	14.74	8.98	4.82	2.85	18.25	13.73	8.25	4.38	2.59
1.50	8.79	8.43	7.30	4.92	2.91	8.77	8.39	7.19	4.80	2.84	8.49	8.07	6.81	4.43	2.61
2.00	4.78	4.75	4.63	4.08	2.89	4.77	4.73	4.60	4.07	2.82	4.65	4.60	4.47	3.85	2.61
	$\rho = 0.75, h_1 = 11.099$					$\rho = 0.90, h_1 = 11.099$					$\rho = 0.95, h_1 = 11.099$				
0.25	3.83	3.84	3.84	3.40	2.02	3.03	3.03	3.01	2.58	1.80	2.36	2.35	2.32	2.02	1.12
0.50	5.98	5.99	5.56	3.50	2.01	4.53	4.52	4.08	2.63	1.88	3.58	3.55	3.16	2.06	1.05
0.75	14.47	11.26	6.27	3.49	2.01	9.67	7.60	4.40	2.67	1.93	7.05	5.68	3.34	2.01	1.01
1.00	252.72	14.20	6.43	3.53	2.03	249.98	8.57	4.43	2.64	1.91	250.90	6.06	3.37	2.01	1.00
1.25	15.24	11.07	6.48	3.63	2.07	10.62	7.56	4.49	2.64	1.82	7.72	5.65	3.44	2.04	1.02
1.50	7.41	6.94	5.66	3.58	2.11	5.50	5.14	4.17	2.64	1.78	4.25	4.00	3.26	2.11	1.10
2.00	4.13	4.10	3.93	3.30	2.19	3.23	3.18	3.02	2.51	1.68	2.58	2.54	2.43	2.02	1.23
						$k_1 = 1.5$									
	$\rho = 0.0, h_1 = 2.747$					$\rho = 0.25, h_1 = 2.747$					$\rho = 0.50, h_1 = 2.747$				
0.25	2.41	2.42	2.42	2.38	1.18	2.41	2.41	2.41	2.33	1.12	2.34	2.35	2.33	2.13	1.03
0.50	5.06	5.03	4.86	2.84	1.32	5.03	5.03	4.80	2.74	1.23	4.82	4.84	4.41	2.43	1.06
0.75	35.34	27.90	8.65	2.95	1.36	34.37	26.37	8.14	2.82	1.32	31.61	22.62	6.46	2.48	1.11
1.00	250.34	40.59	8.89	2.99	1.41	249.33	38.33	8.38	2.89	1.35	250.47	29.90	6.85	2.53	1.19
1.25	19.04	13.96	6.94	3.00	1.44	19.03	13.76	6.84	2.84	1.39	18.39	12.12	6.02	2.52	1.22
1.50	6.28	5.77	4.62	2.81	1.46	6.28	5.70	4.55	2.72	1.41	6.03	5.35	4.20	2.46	1.28
2.00	2.68	2.66	2.52	2.14	1.44	2.67	2.65	2.49	2.11	1.41	2.59	2.55	2.40	1.97	1.30
	$\rho = 0.75, h_1 = 2.795$					$\rho = 0.90, h_1 = 2.84$					$\rho = 0.95, h_1 = 2.84$				
0.25	2.08	2.08	2.07	1.83	1.00	1.56	1.54	1.48	1.16	1.00	1.11	1.10	1.07	1.01	1.00
0.50	3.93	3.91	3.38	1.93	1.00	2.65	2.60	2.22	1.25	1.00	2.00	1.93	1.62	1.04	1.00
0.75	20.94	12.34	4.19	1.94	1.00	8.93	5.57	2.50	1.23	1.00	5.04	3.49	1.86	1.00	1.00
1.00	250.07	16.60	4.41	1.94	1.01	249.84	6.95	2.58	1.21	1.00	250.28	4.09	1.87	1.00	1.00
1.25	15.01	8.72	4.25	1.95	1.03	8.89	5.12	2.66	1.26	1.00	5.47	3.34	1.86	1.03	1.00
1.50	4.98	4.36	3.32	1.92	1.06	3.30	2.92	2.25	1.34	1.00	2.36	2.13	1.67	1.08	1.00
2.00	2.23	2.18	2.04	1.65	1.11	1.65	1.58	1.49	1.24	1.01	1.28	1.26	1.20	1.08	1.00
						$k_1 = 2$									
	$\rho = 0.0, h_1 = 1.37$					$\rho = 0.25, h_1 = 1.39$					$\rho = 0.50, h_1 = 1.39$				
0.25	2.15	2.17	2.12	2.07	1.00	2.14	2.16	2.11	2.03	1.00	2.06	2.11	2.07	1.89	1.00
0.50	5.55	5.59	5.38	2.58	1.01	5.55	5.46	5.26	2.50	1.00	5.36	5.19	4.71	2.16	1.00
0.75	54.40	42.51	11.66	2.67	1.08	52.35	42.80	10.24	2.55	1.05	48.84	35.26	7.95	2.21	1.01
1.00	251.66	53.14	10.64	2.67	1.14	250.23	49.89	10.07	2.60	1.11	249.68	37.83	7.50	2.28	1.04
1.25	20.27	14.19	6.95	2.66	1.20	20.27	14.19	6.95	2.66	1.20	20.11	12.56	5.86	2.16	1.08
1.50	5.92	5.28	4.21	2.43	1.23	5.92	5.19	4.19	2.39	1.21	5.67	4.90	3.83	2.08	1.12
2.00	2.28	2.24	2.09	1.77	1.23	2.28	2.23	2.08	1.77	1.23	2.18	2.10	2.00	1.65	1.15
	$\rho = 0.75, h_1 = 1.41$					$\rho = 0.90, h_1 = 1.46$					$\rho = 0.95, h_1 = 1.49$				
0.25	1.78	1.75	1.71	1.37	1.00	1.18	1.16	1.12	1.03	1.00	1.02	1.03	1.02	1.00	1.00
0.50	4.07	3.87	3.15	1.54	1.00	2.43	2.29	1.82	1.06	1.00	1.62	1.52	1.26	1.01	1.00
0.75	30.48	17.81	4.17	1.56	1.00	11.30	5.97	2.28	1.03	1.00	5.51	3.28	1.50	1.00	1.00
1.00	251.13	19.74	4.40	1.59	1.00	251.07	8.02	2.29	1.06	1.00	249.68	4.02	1.51	1.00	1.00
1.25	16.35	8.88	3.97	1.61	1.00	9.44	5.02	2.30	1.09	1.00	5.48	3.02	1.57	1.01	1.00
1.50	4.56	3.91	2.89	1.60	1.01	2.82	2.54	1.92	1.16	1.00	1.97	1.80	1.39	1.03	1.00
2.00	1.85	1.82	1.73	1.40	1.04	1.35	1.35	1.28	1.13	1.00	1.12	1.13	1.09	1.03	1.00
						$k_1 = 2.5$									
	$\rho = 0.0, h_1 = 0.60$					$\rho = 0.25, h_1 = 0.63$					$\rho = 0.50, h_1 = 0.63$				
0.25	2.05	2.06	2.06	1.98	1.00	2.06	2.07	2.10	1.93	1.00	1.99	1.99	2.01	1.74	1.00
0.50	7.31	7.03	6.40	2.58	1.00	7.51	7.10	6.51	2.45	1.00	6.84	6.43	5.68	2.02	1.00
0.75	71.31	60.66	15.49	2.61	1.04	70.82	62.71	14.46	2.51	1.02	66.72	50.45	10.25	2.10	1.00
1.00	250.83	58.59	12.31	2.67	1.09	251.59	58.15	12.02	2.56	1.07	251.59	45.92	8.40	2.22	1.02
1.25	19.89	14.49	7.16	2.56	1.13	21.04	14.91	6.75	2.45	1.13	20.66	13.20	5.97	2.09	1.06
1.50	5.69	5.05	4.06	2.29	1.18	5.81	5.26	4.10	2.26	1.16	5.58	4.91	3.72	2.06	1.09
2.00	2.11	1.99	2.00	1.65	1.18	2.11	2.03	2.00	1.66	1.16	2.05	1.95	1.88	1.57	1.10
	$\rho = 0.75, h_1 = 0.645$					$\rho = 0.90, h_1 = 0.688$					$\rho = 0.95, h_1 = 0.72$				
0.25	1.59	1.59	1.56	1.23	1.00	1.09	1.09	1.08	1.00	1.00	1.01	1.01	1.00	1.00	1.00
0.50	4.44	4.35	3.39	1.37	1.00	2.32	2.22	1.73	1.04	1.00	1.48	1.38	1.18	1.00	1.00
0.75	41.03	24.30	4.69	1.40	1.00	15.08	7.00	2.15	1.02	1.00	6.31	3.31	1.39	1.00	1.00
1.00	248.57	24.86	4.60	1.45	1.00	251.66	9.02	2.22	1.04	1.00	250.76	4.22	1.38	1.00	1.00
1.25	17.58	9.67	3.94	1.55	1.00	10.25	5.12	2.16	1.07	1.00	5.76	2.96	1.48	1.00	1.00
1.50	4.40	3.77	2.80	1.54	1.01	2.71	2.36	1.80	1.13	1.00	1.86	1.69	1.30	1.02	1.00
2.00	1.72	1.72	1.55	1.32	1.03	1.29	1.29	1.21	1.09	1.00	1.11	1.10	1.07	1.02	1.00

Table 5
ARLs properties of proposed$CMEC_{AIB}$ control chart when $ARL_0 = 250$ and $\lambda_1 = 0.80$

δ_μ	0.00	0.25	0.50	1.0	2.00	0.00	0.25	0.50	1.0	2.00	0.00	0.25	0.50	1.0	2.00
						$k_1 = 0.5$									
δ_γ	$\rho = 0, h_1 = 6.765$					$\rho = 0.25, h_1 = 6.75$					$\rho = 0.50, h_1 = 6.79$				
0.25	3.30	3.33	3.31	3.27	2.00	3.30	3.29	3.29	3.23	2.00	3.25	3.23	3.25	3.07	2.00
0.50	5.97	5.89	5.89	3.82	2.01	5.95	5.86	5.78	3.68	2.00	5.81	5.72	5.56	3.35	2.00
0.75	19.14	16.14	8.68	3.95	2.05	19.08	15.57	8.36	3.84	2.02	18.28	14.44	7.17	3.42	2.00
1.00	249.70	26.78	9.23	3.94	2.09	250.03	25.83	8.70	3.80	2.07	249.05	21.15	7.67	3.47	2.01
1.25	17.85	13.69	8.12	4.00	2.16	17.86	13.39	7.97	3.82	2.09	17.41	12.54	7.16	3.43	1.99
1.50	7.53	6.90	6.05	3.90	2.20	7.52	6.91	5.97	3.82	2.17	7.26	6.67	5.63	3.45	2.01
2.00	3.75	3.69	3.55	3.09	2.15	3.72	3.67	3.52	3.02	2.12	3.65	3.59	3.43	2.88	1.99
	$\rho = 0.75, h_1 = 6.805$					$\rho = 0.90, h_1 = 6.805$					$\rho = 0.95, h_1 = 6.805$				
0.25	2.94	2.93	2.94	2.57	1.73	2.16	2.17	2.12	1.92	1.03	1.95	1.95	1.90	1.51	1.00
0.50	5.01	4.90	4.45	2.71	1.77	3.58	3.51	3.13	2.00	1.00	2.75	2.66	2.32	1.54	1.00
0.75	14.45	10.61	5.28	2.66	1.76	8.79	6.58	3.43	2.00	1.00	6.03	4.56	2.58	1.56	1.00
1.00	250.43	13.80	5.36	2.69	1.69	248.83	7.65	3.50	2.00	1.00	250.60	5.06	2.56	1.56	1.00
1.25	14.29	9.64	5.36	2.73	1.65	9.38	6.33	3.48	2.01	1.02	6.49	4.48	2.62	1.54	1.00
1.50	6.16	5.63	4.47	2.78	1.64	4.36	4.04	3.19	1.99	1.05	3.27	3.04	2.41	1.53	1.00
2.00	3.18	3.14	2.96	2.47	1.61	2.43	2.36	2.25	1.84	1.16	1.90	1.90	1.80	1.43	1.03
						$k_1 = 1.5$									
	$\rho = 0.0, h_1 = 1.98$					$\rho = 0.25, h_1 = 1.96$					$\rho = 0.50, h_1 = 1.96$				
0.25	2.16	2.17	2.16	2.10	1.00	2.15	2.17	2.12	2.01	1.00	2.08	2.12	2.06	1.91	1.00
0.50	6.86	6.80	6.66	2.60	1.02	6.72	6.70	6.43	2.47	1.01	6.30	6.13	5.51	2.14	1.00
0.75	90.67	80.14	16.91	2.77	1.09	85.09	75.38	15.49	2.62	1.07	78.27	60.63	10.07	2.19	1.01
1.00	250.32	64.33	12.42	2.77	1.15	251.64	64.65	11.69	2.62	1.14	250.35	51.67	9.29	2.24	1.05
1.25	21.90	16.33	7.85	2.78	1.22	21.77	15.85	7.18	2.67	1.17	21.14	13.79	6.12	2.27	1.09
1.50	6.13	5.54	4.35	2.54	1.27	6.11	5.38	4.26	2.46	1.22	5.88	5.03	3.87	2.12	1.12
2.00	2.31	2.29	2.12	1.81	1.26	2.30	2.25	2.09	1.78	1.25	2.22	2.15	2.01	1.67	1.16
	$\rho = 0.75, h_1 = 2.01$					$\rho = 0.90, h_1 = 2.06$					$\rho = 0.95, h_1 = 2.10$				
0.25	1.85	1.80	1.78	1.42	1.00	1.22	1.19	1.14	1.04	1.00	1.02	1.04	1.02	1.00	1.00
0.50	4.47	4.34	3.38	1.59	1.00	2.51	2.35	1.86	1.09	1.00	1.68	1.57	1.30	1.01	1.00
0.75	48.23	28.23	4.67	1.57	1.00	15.89	7.29	2.34	1.03	1.00	6.38	3.49	1.58	1.00	1.00
1.00	253.43	27.16	4.83	1.61	1.00	251.07	9.55	2.37	1.07	1.00	250.96	4.43	1.55	1.00	1.00
1.25	19.01	10.28	4.30	1.70	1.01	10.87	5.49	2.31	1.13	1.00	5.93	3.20	1.61	1.01	1.00
1.50	4.77	4.13	2.98	1.61	1.02	2.89	2.61	1.94	1.17	1.00	2.01	1.83	1.40	1.03	1.00
2.00	1.86	1.86	1.77	1.40	1.05	1.37	1.37	1.29	1.13	1.01	1.13	1.14	1.11	1.04	1.00
						$k_1 = 2.0$									
	$\rho = 0.0, h_1 = 1.155$					$\rho = 0.25, h_1 = 1.175$					$\rho = 0.50, h_1 = 1.17$				
0.25	2.16	2.19	2.16	2.06	1.00	2.18	2.21	2.18	2.01	1.00	2.06	2.09	2.05	1.80	1.00
0.50	12.51	12.63	12.63	2.90	1.00	12.51	12.79	12.48	2.72	1.00	11.05	10.83	10.07	2.20	1.00
0.75	151.48	133.82	40.78	2.98	1.05	151.07	133.47	35.17	2.84	1.03	139.08	118.01	20.64	2.27	1.01
1.00	250.12	80.40	16.93	3.05	1.10	251.13	80.22	15.71	2.90	1.09	249.47	69.98	11.84	2.33	1.02
1.25	23.39	17.74	9.05	2.80	1.15	23.79	17.47	8.44	2.68	1.13	24.85	16.52	7.07	2.36	1.07
1.50	6.25	5.73	4.36	2.44	1.19	6.24	5.64	4.39	2.41	1.17	6.08	5.24	4.02	2.10	1.09
2.00	2.20	2.20	2.00	1.72	1.19	2.20	2.19	2.00	1.68	1.17	2.09	2.04	1.96	1.57	1.12
	$\rho = 0.75, h_1 = 1.215$					$\rho = 0.90, h_1 = 1.28$					$\rho = 0.95, h_1 = 1.32$				
0.25	1.68	1.66	1.62	1.23	1.00	1.13	1.12	1.08	1.02	1.00	1.01	1.02	1.01	1.00	1.00
0.50	6.34	6.14	4.21	1.45	1.00	2.60	2.38	1.78	1.05	1.00	1.54	1.47	1.22	1.00	1.00
0.75	48.23	28.23	4.67	1.57	1.00	29.07	11.52	2.40	1.02	1.00	9.40	4.15	1.42	1.00	1.00
1.00	251.13	40.35	6.33	1.53	1.00	252.87	13.46	2.35	1.03	1.00	249.33	5.39	1.48	1.00	1.00
1.25	19.01	10.28	4.30	1.70	1.01	13.35	6.02	2.35	1.08	1.00	7.12	3.38	1.56	1.00	1.00
1.50	5.05	4.16	3.00	1.51	1.01	2.96	2.62	1.85	1.13	1.00	1.94	1.77	1.35	1.03	1.00
2.00	1.77	1.75	1.64	1.35	1.04	1.29	1.31	1.24	1.10	1.00	1.10	1.10	1.08	1.02	1.00
						$k_1 = 2.5$									
	$\rho = 0.0, h_1 = 0.586$					$\rho = 0.25, h_1 = 0.608$					$\rho = 0.50, h_1 = 0.612$				
0.25	2.44	2.51	2.49	2.34	1.00	2.44	2.54	2.49	2.28	1.00	2.31	2.36	2.36	1.85	1.00
0.50	22.73	23.04	20.26	3.90	1.00	22.73	22.77	21.19	3.51	1.00	19.31	18.73	17.79	2.40	1.00
0.75	191.02	171.25	56.62	3.69	1.02	196.82	172.55	51.15	3.40	1.02	161.06	136.39	33.43	2.47	1.00
1.00	250.93	96.74	21.55	3.33	1.08	248.71	98.60	20.79	3.11	1.08	249.58	82.97	15.40	2.40	1.02
1.25	24.78	18.87	9.91	2.99	1.13	25.08	18.60	9.67	2.92	1.12	25.23	17.27	8.04	2.35	1.05
1.50	6.81	6.00	4.52	2.50	1.18	7.03	6.13	4.62	2.43	1.15	6.52	5.84	4.28	2.08	1.08
2.00	2.18	2.06	2.06	1.69	1.19	2.18	2.14	2.05	1.68	1.18	2.11	2.02	1.95	1.57	1.11
	$\rho = 0.75, h_1 = 0.64$					$\rho = 0.90, h_1 = 0.70$					$\rho = 0.95, h_1 = 0.736$				
0.25	1.67	1.65	1.62	1.25	1.00	1.09	1.10	1.08	1.00	1.00	1.01	1.01	1.00	1.00	1.00
0.50	9.68	9.92	6.00	1.40	1.00	2.95	2.70	1.84	1.04	1.00	1.55	1.43	1.19	1.00	1.00
0.75	98.30	64.11	9.95	1.46	1.00	41.07	15.98	2.54	1.01	1.00	14.03	4.90	1.44	1.00	1.00
1.00	250.35	48.83	7.70	1.52	1.00	249.21	17.31	2.65	1.02	1.00	249.88	6.74	1.42	1.00	1.00
1.25	23.35	13.84	4.79	1.56	1.00	15.23	6.77	2.41	1.09	1.00	8.39	3.73	1.50	1.00	1.00
1.50	5.31	4.50	3.17	1.56	1.01	3.10	2.62	1.87	1.12	1.00	1.97	1.76	1.31	1.02	1.00
2.00	1.76	1.78	1.56	1.32	1.03	1.30	1.30	1.22	1.08	1.00	1.11	1.10	1.07	1.02	1.00

Table 6
ARLs properties of some existing control charts when.$ARL_0 = 250$

	CMEC $\lambda = 0.80, k = 1.5$ δ_μ						CMDEC $\lambda = 0.80, k = 0.50, h = 8.590$ δ_μ				
δ_γ	0	0.25	0.5	1	2	δ_γ	0	0.25	0.5	1	2
0.25	2.16	2.17	2.16	2.10	1.00	0.25	2.83	2.82	2.82	2.78	1.16
0.50	6.86	6.80	6.66	2.60	1.02	0.50	5.50	5.51	5.41	3.24	1.31
1.00	250.32	64.33	12.42	2.77	1.15	1.00	250.00	24.45	8.33	3.35	1.41
1.50	6.13	5.54	4.35	2.54	1.27	1.50	6.66	6.30	5.31	3.25	1.45
2.00	2.31	2.29	2.12	1.81	1.26	2.00	2.94	2.91	2.79	2.38	1.45
	CC $k = 1.50, h = 1.715$ δ_μ						MaxDEWMA $\lambda = 0.8, k_1 = 3.251, k_2 = 3.246$ δ_μ				
δ_γ	0	0.25	0.5	1	2	δ_γ	0	0.25	0.5	1	2
0.25	1.40	1.40	1.40	1.27	1.00	0.25	2.20	2.20	2.20	2.10	1.00
0.50	12.19	12.07	11.94	1.95	1.00	0.50	13.90	13.90	13.60	3.10	1.00
1.00	250.00	94.31	18.41	2.25	1.00	1.00	250.00	81.20	17.40	3.00	1.10
1.50	5.54	5.04	3.78	1.82	1.01	1.50	6.60	5.90	4.50	2.40	1.20
2.00	1.63	1.59	1.50	1.26	1.03	2.00	2.20	2.20	2.10	1.70	1.20
	$MaxEWMA_{AIB}$ $\lambda = 0.80, L = 3.25, \rho = 0.95$ δ_μ						$MaxHEWMA_{AIB}$ $\lambda_1, \lambda_2 = 0.80, L = 3.425, \rho = 0.95$ δ_μ				
δ_γ	0	0.25	0.5	1	2	δ_γ	0	0.25	0.5	1	2
0.50	21.36	20.92	18.95	2.52	1.00	0.50	1.50	1.39	1.17	1.00	1.00
0.75	153.50	126.23	33.86	2.69	1.00	0.75	10.13	4.26	1.38	1.00	1.00
1.00	251.09	77.66	16.30	2.57	1.02	1.00	251.66	5.54	1.39	1.00	1.00
1.25	25.97	16.58	8.24	2.39	1.06	1.25	7.18	3.36	1.50	1.00	1.00
1.50	6.66	5.54	4.23	2.06	1.08	1.50	1.90	1.71	1.30	1.02	1.00

ARL_0 needed to be large enough to avoid frequent false alarms. However, the ARL_1 should be small enough; it quickly detects the shift. It is necessary for better performance of a control chart; it should have a smaller ARL_1 as compared to other control charts at a fixed value of ARL_0.

4.3. Choices of parameters in the simulation study

The design parameters (k_1, h_1, and λ_1) of the proposed $CMEC_{AIB}$ control chart has their effect on the detection ability. The sensitivity of shifts guides us in practice to choose these design parameters like k_1 and h_1. According to Gan (1991), perform a sensitivity analysis by comparing the ARL_1 for the (k_1,h_1) combination, to other choices of (k_1,h_1) at the same pre-specified ARL_0. The CUSUM control chart is selected which offers the most desirable performance in terms of ARL_1. In this regard, different authors considered various values of k_1 with h_1 to get the desired ARL_0. For example, Sanusi et al. (2017) used $k_1 = 0.25, 0.50, 0.75, 1.00$, whereas, Zaman, Riaz, et al. (2016) used $k_1 = 0.1, 0.5, 1.0, 1.5, 2.0$. At the other hand, λ_1 is parameter of $EWMA^{(1)}_{AIB}$ and $EWMA^{(2)}_{AIB}$ control charts that are used as input statistics in CUSUM control chart to formulate the proposed control chart. Generally, the EWMA control chart detects out-of-control signal for smaller shifts more quickly under smaller value of λ_1. Following Zaman, Riaz, et al. (2016), the parameter k_1 is taken as 0.5, 1.5, 2.0, 2.5 and λ_1 is set as 0.10, 0.20, 0.50, 0.80 with different values of h_1, to obtain $ARL_0 = 250$. Similarly, Garcia and Cebrian (1996) suggested the population correlation ρmay be pre-defined in many real-world situations. So, the correlation coefficient ρ is assumed as: 0.00, 0.25, 0.50, 0.75, 0.90 and 0.95. However, the values for ρ^* for this study are taken from (Haq, 2017b). He suggested different values for ρ that are 0.25, 0.50, 0.75, 0.90, and 0.95, and based on these values computed the values of ρ^* via simulation study of 10 million replicates with $n = 5$. The results for the pair (ρ, ρ^*) are given as $(\rho, \rho^*) = (0.25, 0.0563898), (0.50, 0.2293317), (0.75, 0.5313626), (0.90, 0.7870992), (0.95, 0.8880799)$. Numerical results of the proposed $CMEC_{AIB}$ chart are presented in Tables 2–5.

5. Evaluation and performance comparison

This section explains the comparative analysis of the proposed $CMEC_{AIB}$ control chart against CMEC, CMDEC, CC, MaxDEWMA, $MaxEWMA_{AIB}$, and $MaxHEWMA_{AIB}$ control charts. More details are given in consequent subsections.

5.1. Proposed $CMEC_{AIB}$ versus CMEC control chart

Zaman, Riaz, et al. (2016) suggested the CMEC control chart for the simultaneous monitoring of the process parameters. The proposed $CMEC_{AIB}$ control chart is reduced to CMEC control chart for $\rho = 0$ (see Section 3.4.2). So, Tables 2–5 also provide numerical results of CMEC when $\rho = 0$. The results show that for all choices of λ_1, k_1, and h_1, the $CMEC_{AIB}$ control chart provides better performance as compared to the CMEC control chart. For example, at$\lambda_1 = 0.80, k_1 = 1.5$, $\delta_\mu = 0.25$, and $\delta_\gamma = 0.50$, the proposed $CMEC_{AIB}$ control chart ($\rho = 0.95$) detects an out-of-control signal after one sample on average, whereas the CMEC control chart ($\rho = 0$) captures an out-of-control signal after six samples on average (see Tables 5 versus 6). Likewise, the superiority of the $CMEC_{AIB}$ control chart also can be observed with other choices of λ_1, k_1, and h_1 (see Tables 2–5).

5.2. Proposed $CMEC_{AIB}$ versus CMDEC control chart

Zaman, Riaz, et al. (2016) also proposed the CMDEC control chart for simultaneous monitoring of the process parameters. It is to be noted, the proposed $CMEC_{AIB}$ control chart also performs better as compared to the CMDEC control chart. For instance, at $(\lambda, \lambda_1) = 0.80$, $\delta_\mu = 0.25$, and $\delta_\gamma = 0.50$, the proposed $CMEC_{AIB}$ control chart ($k_1 = 0.5, \rho = 0.95$) detects an out-of-control signal after 2 samples, while the CMDEC control

Table 7
Diagnostic abilities of proposed $CMEC_{AIB}$ control chart.

		$\rho = 0.95$														
		$k_1 = 0.50$					$k_1 = 1.50$					$k_1 = 2.00$				
δ_γ	δ_μ	0.00	0.25	0.50	1.00	2.00	0.00	0.25	0.50	1.00	2.00	0.00	0.25	0.50	1.00	2.00
0.25	$m+$	0	0	0	0	80	4	49	207	759	1000	47	206	475	932	1000
	$m-$	508	228	60	1	0	508	228	60	1	0	508	228	60	1	0
	$v+$	0	0	0	0	0	0	0	0	0	0	0	0	0	0	0
	$v-$	492	772	940	999	920	488	723	733	240	0	445	566	465	67	0
	$++$	0	0	0	0	0	0	0	0	0	0	0	0	0	0	0
	$+-$	0	0	0	0	0	0	0	0	0	0	0	0	0	0	0
	$-+$	0	0	0	0	0	0	0	0	0	0	0	0	0	0	0
	$--$	0	0	0	0	0	0	0	0	0	0	0	0	0	0	0
0.50	$m+$	0	0	0	1	15	0	10	122	846	1000	9	119	463	986	1000
	$m-$	504	143	14	0	0	504	143	14	0	0	504	143	14	0	0
	$v+$	0	0	0	0	0	0	0	0	0	0	0	0	0	0	0
	$v-$	496	857	986	999	985	496	847	864	154	0	487	738	523	14	0
	$++$	0	0	0	0	0	0	0	0	0	0	0	0	0	0	0
	$+-$	0	0	0	0	0	0	0	0	0	0	0	0	0	0	0
	$-+$	0	0	0	0	0	0	0	0	0	0	0	0	0	0	0
	$--$	0	0	0	0	0	0	0	0	0	0	0	0	0	0	0
1.00	$m+$	0	17	124	307	553	0	148	502	975	1000	1	238	746	999	1000
	$m-$	690	71	0	0	0	690	68	0	0	0	690	66	0	0	0
	$v+$	0	0	0	0	0	0	0	0	0	0	0	1	0	0	0
	$v-$	310	912	876	693	447	310	784	498	25	0	309	695	254	1	0
	$++$	0	0	0	0	0	0	0	0	0	0	0	0	0	0	0
	$+-$	0	0	0	0	0	0	0	0	0	0	0	0	0	0	0
	$-+$	0	0	0	0	0	0	0	0	0	0	0	0	0	0	0
	$--$	0	0	0	0	0	0	0	0	0	0	0	0	0	0	0
1.50	$m+$	5	140	656	941	965	37	316	747	986	1000	82	360	741	986	1000
	$m-$	940	611	162	2	0	789	390	84	2	0	678	283	56	2	0
	$v+$	22	168	86	0	0	146	250	137	9	0	219	325	181	12	0
	$v-$	33	81	96	57	35	28	44	32	3	0	21	32	22	0	0
	$++$	0	0	0	0	0	0	0	0	0	0	0	0	0	0	0
	$+-$	0	0	0	0	0	0	0	0	0	0	0	0	0	0	0
	$-+$	0	0	0	0	0	0	0	0	0	0	0	0	0	0	0
	$--$	0	0	0	0	0	0	0	0	0	0	0	0	0	0	0
2.00	$m+$	26	97	286	843	997	110	251	471	831	997	167	340	531	865	998
	$m-$	886	701	445	56	0	621	397	205	20	0	540	329	168	17	0
	$v+$	79	193	258	87	0	264	348	321	146	3	289	329	300	115	2
	$v-$	9	9	11	14	3	5	4	3	3	0	4	2	1	3	0
	$++$	0	0	0	0	0	0	0	0	0	0	0	0	0	0	0
	$+-$	0	0	0	0	0	0	0	0	0	0	0	0	0	0	0
	$-+$	0	0	0	0	0	0	0	0	0	0	0	0	0	0	0
	$--$	0	0	0	0	0	0	0	0	0	0	0	0	0	0	0

chart($k = 0.5$) detects after five samples on average (see Table 5 versus Table 6).

5.3. Proposed $CMEC_{AIB}$ versus CC control chart

The CC control chart to monitor the process parameters jointly also introduced by Zaman, Riaz, et al. (2016). The $CMEC_{AIB}$ control chart performs superior against the CC control chart, too. For instance, at $k_1 = 1.5$, $\lambda_1 = 0.8$, $\rho = 0.95$, $\delta_\mu = 0.25$, and $\delta_\gamma = 1$, the $CMEC_{AIB}$ control chart detects out-of-control signals after four samples, whereas the CC control chart detects out-of-control signals after 94 samples on average (see Table 5 versus Table 6).

5.4. Proposed $CMEC_{AIB}$ versus MaxDEWMA control chart

Khoo, Teh, and Wu (2010) generalized the idea of Chen, Cheng, and Xie (2001) and designed the MaxDEWMA control chart for simultaneous monitoring. The proposed $CMEC_{AIB}$ control chart also illustrates outstanding performance against the MaxDEWMA control chart. For example, at $(\lambda, \lambda_1) = 0.8$, $\rho = 0.95$, $k_1 = 1.5$, $\delta_\mu = 0.25$, and $\delta_\gamma = 0.75$, the $CMEC_{AIB}$ control chart detects out-of-control signals after three samples on average, whereas the MaxDEWMA control chart detects out-of-control signals after 13 samples on average (see Table 5 versus Table 6).

5.5. Proposed $CMEC_{AIB}$ versus $MaxEWMA_{AIB}$ control chart

The $MaxEWMA_{AIB}$ control chart presented by Haq (2017b) for the simultaneous monitoring of the process parameters. The proposed $CMEC_{AIB}$ control chart performs better than the $MaxEWMA_{AIB}$ control chart. More specifically, at $(\lambda, \lambda_1) = 0.80$, $\rho = 0.95$, $k_1 = 1.5$, $\delta_\mu = 0.25$, and $\delta_\gamma = 1.25$, the proposed $CMEC_{AIB}$ control chart detects an out-of-control signal after 3 samples on average, although the $MaxEWMA_{AIB}$ control chart caught after 16 samples on average (see Table 5 versus Table 6).

Table 8
Diagnostic abilities of the CMEC control chart.

							$\rho = 0.00$									
		$k = 0.5$					$k = 1.5$					$k = 2$				
δ_γ	δ_μ	0.00	0.25	0.50	1.0	2.00	0.00	0.25	0.50	1.0	2.00	0.00	0.25	0.50	1.0	2.00
0.25	$m+$	0	0	0	0	0	0	0	0	0	0	0	0	0	0	999
	$m-$	506	19	0	0	0	506	19	0	0	0	506	19	0	0	0
	$v+$	0	0	0	0	0	0	0	0	0	0	0	0	0	0	0
	$v-$	494	981	1000	1000	1000	494	981	1000	1000	1000	494	981	1000	1000	1
	$++$	0	0	0	0	0	0	0	0	0	0	0	0	0	0	0
	$+-$	0	0	0	0	0	0	0	0	0	0	0	0	0	0	0
	$-+$	0	0	0	0	0	0	0	0	0	0	0	0	0	0	0
	$--$	0	0	0	0	0	0	0	0	0	0	0	0	0	0	0
0.50	$m+$	0	0	0	0	0	0	0	0	0	38	0	0	0	3	935
	$m-$	510	161	19	0	0	510	161	19	0	0	510	161	19	0	0
	$v+$	0	0	0	0	0	0	0	0	0	0	0	0	0	0	0
	$v-$	490	839	981	1000	1000	490	839	981	1000	962	490	839	981	997	65
	$++$	0	0	0	0	0	0	0	0	0	0	0	0	0	0	0
	$+-$	0	0	0	0	0	0	0	0	0	0	0	0	0	0	0
	$-+$	0	0	0	0	0	0	0	0	0	0	0	0	0	0	0
	$--$	0	0	0	0	0	0	0	0	0	0	0	0	0	0	0
1.00	$m+$	0	0	0	31	152	0	1	34	228	599	1	12	58	361	882
	$m-$	679	475	256	29	0	679	475	253	28	0	679	473	251	28	0
	$v+$	0	0	0	0	0	0	0	0	0	0	0	0	0	0	0
	$v-$	321	525	744	940	848	321	524	713	744	401	320	515	691	611	118
	$++$	0	0	0	0	0	0	0	0	0	0	0	0	0	0	0
	$+-$	0	0	0	0	0	0	0	0	0	0	0	0	0	0	0
	$-+$	0	0	0	0	0	0	0	0	0	0	0	0	0	0	0
	$--$	0	0	0	0	0	0	0	0	0	0	0	0	0	0	0
1.50	$m+$	0	6	31	257	591	13	44	150	485	865	25	92	207	563	919
	$m-$	858	775	625	247	4	822	701	511	161	3	780	646	464	133	2
	$v+$	6	9	19	20	0	37	69	95	71	4	71	100	121	93	14
	$v-$	136	210	325	476	405	128	186	244	283	128	124	162	208	211	65
	$++$	0	0	0	0	0	0	0	0	0	0	0	0	0	0	0
	$+-$	0	0	0	0	0	0	0	0	0	0	0	0	0	0	0
	$-+$	0	0	0	0	0	0	0	0	0	0	0	0	0	0	0
	$--$	0	0	0	0	0	0	0	0	0	0	0	0	0	0	0
2.00	$m+$	0	12	43	238	773	33	81	169	410	859	64	124	224	480	871
	$m-$	931	871	760	452	39	796	694	550	268	17	702	591	448	203	12
	$v+$	16	38	77	138	22	125	167	204	233	72	195	235	267	259	85
	$v-$	53	79	120	172	166	46	58	77	89	52	39	50	61	58	32
	$++$	0	0	0	0	0	0	0	0	0	0	0	0	0	0	0
	$+-$	0	0	0	0	0	0	0	0	0	0	0	0	0	0	0
	$-+$	0	0	0	0	0	0	0	0	0	0	0	0	0	0	0
	$--$	0	0	0	0	0	0	0	0	0	0	0	0	0	0	0

5.6. Proposed $CMEC_{AIB}$ versus $MaxHEWMA_{AIB}$ control chart

Javaid et al. (2020) suggested the $MaxHEWMA_{AIB}$ control chart for the simultaneous monitoring of the process parameters. The results show that for some choices of λ_1, the $CMEC_{AIB}$ control chart provides better performance as compared to the $MaxHEWMA_{AIB}$ control chart. For example, at $\rho = 0.95, k_1 = 1.5,$ for $\delta_\mu = 0.25$ and $\delta_\gamma = 0.75$, the proposed $CMEC_{AIB}$ control chart ($\lambda_1 = 0.80$) detects an out-of-control signal after 3 samples on average, whereas the $MaxHEWMA_{AIB}$ control chart ($\lambda_1 = \lambda_2 = 0.80$) captures an out-of-control signal after four samples on average (see Table 5 versus Table 6).

5.7. Diagnosing ability

Simultaneously monitoring of the process parameters required the source and the direction of an out-of-control point. The source may be a location or dispersion parameter, and the direction may be upward or downward. Therefore, to understand the performance of the proposed $CMEC_{AIB}$ control chart, it is necessary to explore the diagnostic abilities of the $CMEC_{AIB}$ control chart. Following the idea of (Chen & Cheng, 1998), at $\lambda_1 = 0.3, \rho = 0, 0.95,$ and $n = 5$, the 10^3 simulations for out-of-control signals are performed, and the number of out-of-control signals caused by the proposed $CMEC_{AIB}$ control chart, are counted. The results of the diagnostic abilities for the proposed $CMEC_{AIB}$ and other control charts are provided in Tables 7–9. It is noted that for the in-control process, the control charts give the signal for an upward or downward at the same rate. For the out-of-control situation, the proposed $CMEC_{AIB}$ control chart quickly detects the out-of-control signals for the different possible directions ($m+, m-, v+, v-$) of change .For example, at $k/k_1 = 1.5, \delta_\mu = 0.5,$ and $\delta_\gamma = 0.25$, the out-of-control signals in different possible directions are (207, 60, 0, 733) for $CMEC_{AIB}$ control chart, and (0,0,0,1000) for the CMEC control chart. It shows that the $CMEC_{AIB}$ has the edge over the CMEC control chart (see Table 7 vs. Table 8). Similarly, the $CMEC_{AIB}$ control chart performs better against the MaxEWMA and MaxDEWMA control charts as well (see Table 7 vs. Table 9). It is interesting to note that no out-of-control signals correspond to "++,"

Table 9
Diagnostic abilities of the some existing control chart.

δ_γ	δ_μ	MaxEWMA 0.00	0.25	0.50	1.00	2.00	MaxDEWMA 0.00	0.25	0.50	1.00	2.00
0.25	$m+$	0	0	0	45	985	0	0	0	6	100
	$m-$	0	0	0	0	0	0	0	0	0	0
	$v+$	0	0	0	0	0	0	0	0	0	0
	$v-$	1000	1000	1000	658	0	1000	1000	100	524	0
	$++$	0	0	0	0	0	0	0	0	0	0
	$+-$	0	0	0	297	15	0	0	0	470	0
	$-+$	0	0	0	0	0	0	0	0	0	0
	$--$	0	0	0	0	0	0	0	0	0	0
0.50	$m+$	0	0	68	864	1000	0	0	35	940	1000
	$m-$	0	0	0	0	0	0	0	0	0	0
	$v+$	0	0	0	0	0	6	6	5	0	0
	$v-$	1000	1000	864	22	0	994	994	882	4	0
	$++$	0	0	0	0	0	0	0	0	0	0
	$+-$	0	0	68	114	0	0	0	78	56	0
	$-+$	0	0	0	0	0	0	0	0	0	0
	$--$	0	0	0	0	0	0	0	0	0	0
1.00	$m+$	272	976	993	1000	1000	239	852	919	990	1000
	$m-$	245	0	0	0	0	255	120	76	10	0
	$v+$	238	10	4	0	0	236	11	1	0	0
	$v-$	244	13	0	0	0	270	16	3	0	0
	$++$	1	0	3	0	0	0	1	0	0	0
	$+-$	0	1	0	0	0	0	0	1	0	0
	$-+$	0	0	0	0	0	0	0	0	0	0
	$--$	0	0	0	0	0	0	0	0	0	0
1.5	$m+$	27	169	405	794	977	5	72	310	843	997
	$m-$	37	3	0	0	0	11	18	51	36	2
	$v+$	923	788	526	120	4	884	797	476	48	0
	$v-$	0	0	0	0	0	95	82	64	8	0
	$++$	6	40	69	86	19	0	18	80	52	1
	$+-$	0	0	0	0	0	2	0	8	10	0
	$-+$	7	0	0	0	0	3	11	11	3	0
	$--$	0	0	0	0	0	0	2	0	0	0

"+−," "−+," and "−−" even though in most cases. Since our proposed control chart is the extension of the CMEC control chart proposed by Zaman, Riaz, et al. (2016) and similar behavior can be seen in the CMEC control chart. It may be possible that when the shifts occur in both location and dispersion parameters, their effect will be either confounding each other or will counterbalance the effect of each other, and hence we cannot note any signal corresponds to "++," "+−," "−+," and "−−."

5.8. Main outcomes of the study

Some interesting outcomes of the proposed $CMEC_{AIB}$ control chart are listed as follows:

(i) The mixed technique improves the performance of the proposed $CMEC_{AIB}$ control chart.
(ii) The use of auxiliary information certainly increases the shift detection ability of the proposed $CMEC_{AIB}$ control chart (see Table 2–5).
(iii) The ARL values of the proposed $CMEC_{AIB}$ control chart are smaller as compared to CMEC, CMDEC, CC, MaxDEWMA, $MaxEWMA_{AIB}$, and $MaxHEWMA_{AIB}$ control charts at the different choices of parameters.
(iv) At high values of the ρ, the proposed $CMEC_{AIB}$ control chart provides the best performance.
(v) The proposed $CMEC_{AIB}$ control chart produces smaller ARL_1 for higher values of ρ, λ_1, and k_1.
(vi) As the h_1 decreases, the λ_1 and k_1 increased.
(vii) The proposed $CMEC_{AIB}$ control chart has better diagnostic abilities as well.

6. Real-life application

This section describes the real-life application of the proposed $CMEC_{AIB}$ versus other existing CMEC control charts. Section 6.1 contains the detail of the variable of interest and the implementation procedure of the proposed $CMEC_{AIB}$ control chart for real-life data is provided in Section 6.2.

6.1. Variable of interest

The wall thicknesses and strength levels are two very essential characteristics of the bottles and glass containers process. The thin glass can result in damage during the filling process or transportation, and thick glass can result in excessive materials consumption (Hinken & Beller, 2006). Similarly, hardness is combined with transparency, and chemical resistance causes the extensive application of glass products. Modern high-rise commercial and residential buildings, sports complexes, military, space technology, glass bottles, all types of transport, and even every phase of life require glasses with high strength (Min'ko & Nartsev, 2013). Also, the glass hardness and strength always depend on the thickness level of the glass wall. Different methods, such as the thickness measuring system for container glass (TMC), are widely used by glass manufacturers. The TMC system can measure during dynamic

Table 10
Real-life dataset example with proposed $CMEC_{AIB}$ versus CMEC control charts.

	y_{i1}	y_{i2}	y_{i3}	y_{i4}	y_{i5}	x_{i1}	x_{i2}	x_{i3}	x_{i4}	x_{i5}
1	5.69	17.97	25.41	23.08	18.13	0.27	1.38	2.01	1.71	1.41
2	29.09	22.65	18.02	22.76	24.29	2.22	1.69	1.40	1.70	1.87
3	10.3	20.13	24.88	4.47	15.56	0.75	1.57	1.97	0.19	1.20
4	5.83	18.17	19.69	13.40	9.54	0.28	1.43	1.55	0.97	0.72
5	8.31	18.63	8.19	24.23	15.04	0.61	1.49	0.61	1.86	1.14
6	23.36	11.68	21.8	14.89	7.16	1.74	0.85	1.64	1.12	0.49
7	3.43	12.45	15.26	18.64	14.16	0.12	0.91	1.19	1.50	1.05
8	19.67	20.40	13.78	28.32	26.15	1.54	1.61	1.02	2.19	2.10
9	13.62	11.90	21.82	30.31	23.87	0.98	0.86	1.65	2.33	1.79
10	24.52	27.40	12.74	31.33	17.29	1.91	2.15	0.94	2.39	1.34
11	25.62	16.05	22.53	27.31	7.23	2.06	1.24	1.69	2.14	0.54
12	19.14	19.85	19.48	11.65	23.12	1.50	1.55	1.54	0.84	1.74
13	4.86	5.17	19.44	15.31	17.16	0.20	0.20	1.52	1.20	1.33
14	17.05	24.27	9.45	9.12	18.61	1.31	1.86	0.68	0.66	1.47
15	19.5	16.15	25.18	26.80	7.98	1.54	1.24	1.98	2.12	0.55
16	18.37	20.9	16.43	17.51	18.78	1.46	1.61	1.25	1.35	1.50
17	23.77	29.65	16.46	26.54	24.78	1.77	2.31	1.26	2.12	1.94
18	9.41	6.41	36.95	2.66	24.03	0.67	0.43	2.57	0.04	1.84
19	38.23	24.38	17.04	24.64	10.14	3.26	1.89	1.31	1.92	0.74
20	5.85	31.38	18.34	10.06	20.37	0.37	2.42	1.45	0.74	1.60
21	17.34	23.27	27.75	27.27	29.24	0.76	1.15	1.45	1.37	1.51
22	9.51	20.45	48.03	11.30	25.43	0.41	0.96	2.49	0.50	1.29
23	32.09	26.18	22.22	30.54	18.08	1.64	1.32	1.07	1.56	0.86
24	28.76	13.28	15.08	26.22	38.40	1.50	0.64	0.73	1.33	1.99
25	41.13	21.90	43.52	20.93	35.73	2.14	1.07	2.20	0.99	1.76
26	22.80	25.25	32.99	21.30	37.59	1.12	1.28	1.64	1.03	1.94
27	51.80	22.40	43.59	21.44	22.85	2.57	1.09	2.20	1.04	1.13
28	34.11	19.68	40.08	33.71	8.57	1.68	0.94	2.12	1.67	0.26
29	17.37	33.32	25.98	44.31	29.33	0.77	1.67	1.29	2.23	1.51
30	36.32	44.40	40.91	39.11	17.70	1.81	2.29	2.12	2.07	0.80
31	30.15	14.19	33.92	39.50	19.65	1.77	0.75	2.27	2.65	1.47
32	17.30	16.26	16.50	17.93	18.05	1.09	0.85	0.91	1.15	1.22
33	40.52	21.90	47.15	9.53	15.47	2.66	1.48	2.78	0.48	0.81
34	16.71	22.68	6.95	33.17	32.67	1.07	1.49	0.18	2.02	1.88
35	33.36	29.18	25.05	19.02	18.32	2.19	1.67	1.54	1.45	1.30
36	20.79	13.26	26.18	24.84	5.54	1.48	0.70	1.58	1.54	0.15
37	12.58	9.41	35.93	9.801	8.22	0.68	0.46	2.44	0.50	0.36
38	17.46	6.39	19.14	16.53	10.80	1.11	0.16	1.47	1.05	0.52
39	16.11	25.61	35.79	24.24	29.34	0.84	1.56	2.40	1.50	1.74
40	28.95	12.57	35.34	18.90	14.73	1.65	0.61	2.33	1.33	0.79

Continue Table 10: Real-life dataset example with proposed $CMEC_{AIB}$ versus CMEC control charts

	CMEC(ρ = 0)						$CMEC_{AIB}$ (ρ = 0.905)					
	$CMECL_i^+$	$CMECL_i^-$	$CMECV_i^+$	$CMECV_i^-$	H_i	$-H_i$	P_t^+	P_t^-	Q_t^+	Q_t^-	H_i	$-H_i$
1	0.00	0.00	0.00	0.00	1.17	−1.17	0.00	0.00	0.00	0.00	1.19	−1.19
2	0.00	0.00	0.00	0.00	1.43	−1.43	0.00	−1.04	0.00	0.00	1.45	−1.45
3	0.00	0.00	0.00	0.00	1.54	−1.54	0.00	−0.99	0.00	0.00	1.57	−1.57
4	0.00	0.00	0.00	0.00	1.59	−1.59	0.00	−0.12	0.00	0.00	1.62	−1.62
5	0.00	0.00	0.00	0.00	1.62	−1.62	0.00	0.00	0.00	0.00	1.64	−1.64
6	0.00	0.00	0.00	0.00	1.63	−1.63	0.18	0.00	0.00	0.00	1.66	−1.66
7	0.00	0.00	0.00	0.00	1.63	−1.63	0.71	0.00	0.00	0.00	1.66	−1.66
8	0.00	0.00	0.00	0.00	1.64	−1.64	0.00	0.00	0.00	0.00	1.66	−1.66
9	0.00	0.00	0.00	0.00	1.64	−1.64	0.00	0.00	0.00	0.00	1.67	−1.67
10	0.00	0.00	0.00	0.00	1.64	−1.64	0.00	−0.51	0.00	0.00	1.67	−1.67
11	0.00	0.00	0.00	0.00	1.64	−1.64	0.00	−0.95	0.00	0.00	1.67	−1.67
12	0.00	0.00	0.00	0.00	1.64	−1.64	0.00	−1.76	0.00	0.00	1.67	−1.67
13	0.00	0.00	0.00	−0.05	1.64	−1.64	0.00	−1.04	0.00	−0.05	1.67	−1.67
14	0.00	0.00	0.00	−0.05	1.64	−1.64	0.00	−0.08	0.00	−0.05	1.67	−1.67
15	0.00	0.00	0.00	−0.03	1.64	−1.64	0.00	0.00	0.00	−0.03	1.67	−1.67
16	0.00	0.00	0.00	−0.17	1.64	−1.64	0.00	−0.41	0.00	−0.17	1.67	−1.67
17	0.00	0.00	0.00	−0.29	1.64	−1.64	0.00	−2.14	0.00	−0.29	1.67	−1.67
18	0.00	0.00	0.00	−0.05	1.64	−1.64	0.00	−1.54	0.00	−0.05	1.67	−1.67
19	0.00	0.00	0.00	0.00	1.64	−1.64	0.00	−1.70	0.00	0.00	1.67	−1.67
20	0.00	0.00	0.00	0.00	1.64	−1.64	0.00	−1.23	0.00	0.00	1.67	−1.67
21	0.00	0.00	0.00	0.00	1.64	−1.64	0.00	−0.82	0.00	0.00	1.67	−1.67
22	0.00	0.00	0.00	0.00	1.64	−1.64	0.68	0.00	0.00	0.00	1.67	−1.67
23	0.00	0.00	0.00	0.00	1.64	−1.64	0.91	0.00	0.00	0.00	1.67	−1.67
24	0.00	0.00	0.03	0.00	1.64	−1.64	1.58	0.00	0.03	0.00	1.67	−1.67
25	0.00	0.00	0.25	0.00	1.64	−1.64	1.80	0.00	0.25	0.00	1.67	−1.67
26	0.00	0.00	0.43	0.00	1.64	−1.64	1.69	0.00	0.43	0.00	1.67	−1.67
27	0.00	0.00	1.01	0.00	1.64	−1.64	2.00	0.00	1.01	0.00	1.67	−1.67
28	0.12	0.00	1.66	0.00	1.64	−1.64	2.92	0.00	1.66	0.00	1.67	−1.67
29	0.08	0.00	2.29	0.00	1.64	−1.64	3.29	0.00	2.29	0.00	1.67	−1.67

(continued on next page)

Table 10 (*continued*)

Continue Table 10: Real-life dataset example with proposed $CMEC_{AIB}$ versus CMEC control charts

	CMEC(ρ = 0)						$CMEC_{AIB}$ (ρ = 0.905)					
	$CMECL_i^+$	$CMECL_i^-$	$CMECV_i^+$	$CMECV_i^-$	H_i	$-H_i$	P_t^+	P_t^-	Q_t^+	Q_t^-	H_i	$-H_i$
30	0.00	0.00	2.88	0.00	1.64	−1.64	2.56	0.00	2.88	0.00	1.67	−1.67
31	0.00	0.00	3.20	0.00	1.64	−1.64	1.36	0.00	3.20	0.00	1.67	−1.67
32	0.00	0.00	2.42	0.00	1.64	−1.64	0.16	0.00	2.42	0.00	1.67	−1.67
33	0.00	0.00	2.09	0.00	1.64	−1.64	0.00	0.00	2.09	0.00	1.67	−1.67
34	0.00	0.00	1.84	0.00	1.64	−1.64	0.15	0.00	1.84	0.00	1.67	−1.67
35	0.00	0.00	1.69	0.00	1.64	−1.64	0.00	0.00	1.69	0.00	1.67	−1.67
36	0.00	0.00	1.39	0.00	1.64	−1.64	0.07	0.00	1.39	0.00	1.67	−1.67
37	0.00	0.00	1.04	0.00	1.64	−1.64	1.45	0.00	1.04	0.00	1.67	−1.67
38	0.00	0.00	0.25	0.00	1.64	−1.64	3.13	0.00	0.25	0.00	1.67	−1.67
39	0.00	0.00	0.00	0.00	1.64	−1.64	3.43	0.00	0.00	0.00	1.67	−1.67
40	0.00	0.00	0.00	0.00	1.64	−1.64	3.89	0.00	0.00	0.00	1.67	−1.67

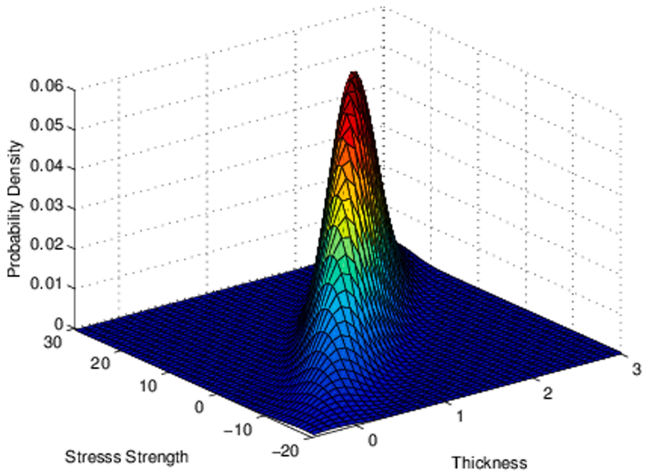

Fig. 1. Probability density plot of bivariate normal distribution using real-life data.

production or random sampling. The TMC system measures the lower and upper acceptable limits and other significant parameters that empower to assess the distribution of glass wall thickness. On the other hand, a general inspection of stress strength is carried out using polarized light, and the residual stress is calculated by American standard ASTM C148. Based on the information obtained by the TMC system and stress strength tests, the manufacturers readjust the glass production system to maintain the quality insurance. The stress strength of glass bottles is highly correlated to the thickness of glass. A real-life data set of the glass thickness (X) and its impact on stress strength (Y) of glass bottles (see Table 10) is taken from Asadzadeh and Kiadaliry (2017).

6.2. *Implementation of $CMEC_{AIB}$ versus CMEC control charts*

To demonstrate the performance of the proposed control chart in real-world situations, we applied the $CMEC_{AIB}$ and CMEC control charts to monitor the stress strength of glass bottles. The data consists of 40 samples of stress strength (kg/cm^2), thickness (cm), and each of size 5. The variables X and Y follow the bivariate normal distribution (see Fig. 1). The goodness of fit is obtained using "mshapiro.test" function in "mvnormtest" package of R software. The numerical value of mshapiro test is 0.949, with p-value is 0.069. This shows that the data is well fitted to the specified distribution. The proposed control chart is constructed under the assumption when parameters are known, but in the case of real-life data application of the proposed control chart, the population parameters are not available. So, for the practical implementation of the control chart, the estimated parameters are used for empirical quantification of the required quantities to show the implementation of the proposed control charts. Therefore, it is necessary to regard the sample

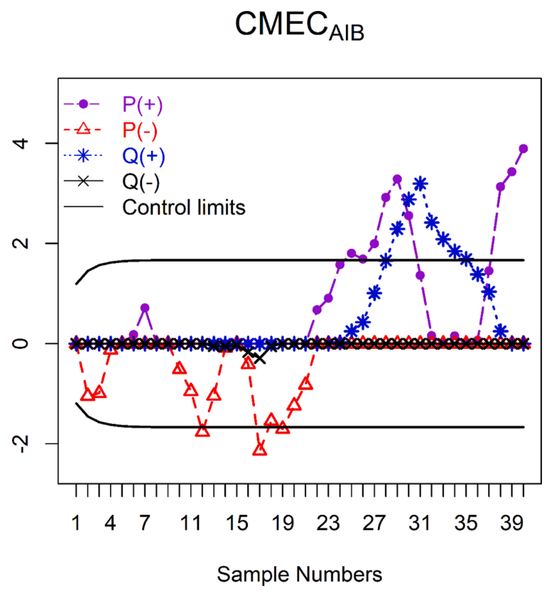

Fig. 2. $CMEC_{AIB}$ control chart when $\lambda = 0.30$, $k_1 = 1.5$, $\rho = 0.905, h_1 = 3.97$, and $ARL_0 = 250$.

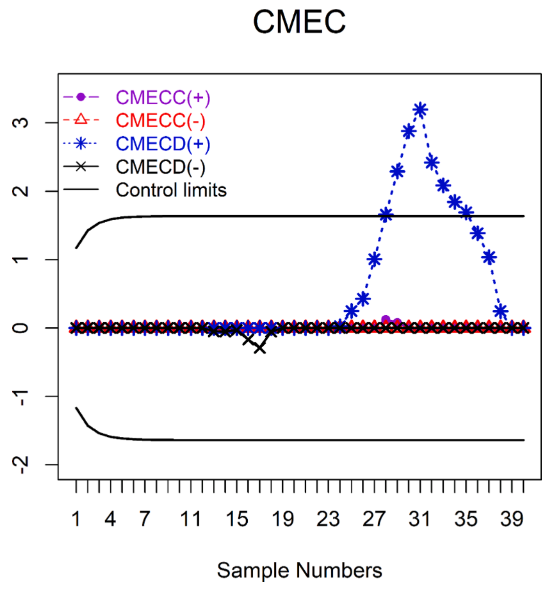

Fig. 3. CMEC control chart when $\lambda = 0.30$, $k = 1.5$, $\rho = 0.0, h = 3.90$, and $ARL_0 = 250$.

Table 11
Dimensions and out-of-control signals for the proposed $CMEC_{AIB}$ versus CMEC control charts.

Source of	CMEC($\rho = 0$)				$CMEC_{AIB}$ ($\rho = 0.905$)			
out of	$k = 0.5$	$k = 1$	$k = 1.5$	$k = 2$	$k_1 = 0.5$	$k_1 = 1$	$k_1 = 1.5$	$k_1 = 2$
Control Signals	$\lambda = 0.3$	$\lambda = 0.3$	$\lambda = 0.3$	$\lambda = 0.3$	$\lambda_1 = 0.3$	$\lambda_1 = 0.3$	$\lambda_1 = 0.3$	$\lambda_1 = 0.3$
$m+$	0	0	0	0	4	8	9	9
$m-$	0	0	0	0	0	0	3	5
$v+$	2	9	7	5	2	9	7	4
$v-$	0	0	0	0	0	0	0	0
**Total**	**2**	**9**	**7**	**5**	**6**	**17**	**19**	**18**

estimates as population parameters (Abbasi & Haq, 2019). The estimates of process parameters are given as: $\hat{\mu}_Y = 6.36$, $\hat{\sigma}_Y = 8.92$, $\hat{\mu}_X = 1.38$, $\hat{\sigma}_X = 0.62$, and $\hat{\rho} = 0.905$. First 20 samples are considered as in-control, the rest 20 samples are treated as out-of-control. By following Haq (2017b) and Anwar, Aslam, Riaz, et al. (2020), we multiply 1.5 to the Y and then add 0.5 for the out-of-control scenario. The design parameters of the proposed $CMEC_{AIB}$ control chart are $\lambda_1 = 0.3, h_1 = 3.9, k_1 = 1.5$ at $ARL_0 = 250$, whereas, the design parameters of the existing CMEC control chart are $\lambda = 0.3, h = 3.9, k = 1.5$ at $ARL_0 = 250$. The proposed $CMEC_{AIB}$ control chart detects the first out-of-control signals at sample number 12 (see Fig. 2), though the CMEC control chart detects the first out-of-control signal at sample number 29 (see Fig. 3). Furthermore, the $CMEC_{AIB}$ control chart overall detects 19 out-of-control points, but the CMEC control chart detects only seven out-of-control. Similarly, the $CMEC_{AIB}$ control chart also can detect out-of-control in different directions (see Table 11). This shows that the $CMEC_{AIB}$ control chart is more sensitive than the CMEC control chart.

7. Summary, conclusions, and recommendations

This study presented a new combined mixed EWMA-CUSUM based auxiliary-information, denoted as $CMEC_{AIB}$ control chart for the simultaneous monitoring of the process parameters (location and/or dispersion). The $CMEC_{AIB}$ control chart is constructed by mixing features of the EWMA and CUSUM control charts in the presence of auxiliary information. The $CMEC_{AIB}$ control chart structure is used two independent plotting statistics of the process location and dispersion (of the EWMA structure) as input for the classical CUSUM control charts. The performance measures such as average run length and diagnostic abilities are used to evaluate the performance of the proposed $CMEC_{AIB}$ control chart. The proposed $CMEC_{AIB}$ control chart outperformed against some existing control charts such as combined mixed EWMA-CUSUM (CMEC), combined double mixed EWMA-CUSUM (CDMEC), combined CUSUM (CC), maximum double EWMA (MaxDEWMA), maximum EWMA based on auxiliary information ($MaxEWMA_{AIB}$), and maximum hybrid EWMA based on auxiliary information ($MaxHEWMA_{AIB}$). Also, the proposed $CMEC_{AIB}$ control chart performs very well for small-to-moderate shifts, especially in the case of large correlation coefficient values. Similarly, the proposed $CMEC_{AIB}$ control chart has better diagnostic abilities against existing control charts, too. Furthermore, it is vital to mention, some existing control charts like the classical CUSUM, CMEC, and MaxCUSUM control charts are special cases of the proposed control $CMEC_{AIB}$ control chart with the special value of their parameters. Finally, to show the vitality of the proposed study from a practical point of view, a real-life case study in the bottle manufacturing industry is also provided. This study has been performed under restriction when a monitoring characteristic follows the normal distribution, but it can be extended for non-normal distribution and the multivariate case as well.

CRediT authorship contribution statement

Syed Masroor Anwar: Conceptualization, Methodology, Software, Formal analysis, Investigation, Data curation, Writing - original draft, Writing - review & editing, Visualization. **Muhammad Aslam:** Conceptualization, Methodology, Validation, Resources, Supervision, Project administration. **Babar Zaman:** Methodology, Validation, Investigation, Resources, Visualization, Supervision. **Muhammad Riaz:** Validation, Resources, Supervision.

References

Abbas, N., Riaz, M., & Does, R. J. M. M. (2012a). CS-EWMA Chart for Monitoring Process Dispersion. *Quality and Reliability Engineering International, 29*, 653–663.
Abbas, N., Riaz, M., & Does, R. J. M. M. (2012b). Mixed exponentially weighted moving average–cumulative sum charts for process monitoring. *Quality and Reliability Engineering International, 29*(3), 345–356.
Abbas, N., Riaz, M., & Does, R. J. M. M. (2014). An EWMA-Type Control Chart for Monitoring the Process Mean Using Auxiliary Information. *Communications in Statistics – Theory and Methods, 43*(16), 3485–3498.
Abbasi, S., & Haq, A. (2019). Optimal CUSUM and adaptive CUSUM charts with auxiliary information for process mean. *Journal of Statistical Computation and Simulation, 89* (2), 337–361. https://doi.org/10.1080/00949655.2018.1548619
Adegoke, N. A., Riaz, M., Sanusi, R. A., Smith, A. N., & Pawley, M. D. (2017). EWMA-type scheme for monitoring location parameter using auxiliary information. *Computers & Industrial Engineering, 114*, 114–119.
Adeoti, Q. A., & Malela-Majika, J. C. (2019). Double exponentially weighted moving average control chart with supplementary runs-rules. *Quality Technology & Quantitative Management.* https://doi.org/10.1080/16843703.2018.1560603
Ahmad, S., Abbasi, S. A., Riaz, M., & Abbas, N. (2014). On efficient use of auxiliary information for control charting in SPC. *Computers & Industrial Engineering, 67*, 173–184.
Ajadi, J. O., & Riaz, M. (2017). Mixed multivariate EWMA-CUSUM control charts for an improved process monitoring. *Communications in Statistics – Theory and Methods, 46* (14), 6980–6993.
Anwar, S. M., Aslam, M., Ahmad, S., & Riaz, M. (2020). A modified-mxEWMA location chart for the improved process monitoring using auxiliary information and its application in wood industry. *Quality Technology & Quantitative Management, 17*(5), 561–579.
Anwar, S. M., Aslam, M., Riaz, M., & Zaman, B. (2020). On mixed memory control charts based on auxiliary information for efficient process monitoring. *Quality and Reliability Engineering International, 36*(6), 1949–1968. https://doi.org/10.1002/qre.2667
Asadzadeh, S., & Kiadaliry, F. (2017). Monitoring type-2 censored reliability data in multistage processes. *Quality and Reliability Engineering International, 33*(8), 2551–2561.
Aslam, M., & Anwar, S. M. (2020). An improved Bayesian Modified-EWMA location chart and its applications in mechanical and sport industry. *PLoS ONE, 15*(2), Article e0229422. https://doi.org/10.1371/journal.pone.0229422
Chao-Wen, L., & Reynolds, M. J. (2018). Control charts for monitoring the mean and variance of autocorrelated processes. *Journal of Quality Technology, 31*(3), 259–274.
Chen, G., & Cheng, S. W. (1998). Max-chart: Combining X-bar chart and S chart. *Statistica Sinica, 8*, 263–271.
Chen, G., Cheng, W. S., & Xie, H. (2001). Monitoring process mean and variability with one EWMA chart. *Journal of Quality Technology, 33*(2), 223–233.
Chen, G., Cheng, S. W., & Xie, H. (2016). A New EWMA Control Chart for Monitoring Both Location and Dispersion. *Quality Technology & Quantitative Management, 1*(2), 217–231.
Chen, G., Cheng, S. W., & Xie, H. (2018). Monitoring process mean and variability with one EWMA chart. *Journal of Quality Technology, 33*(2), 223–233.
Gan, F. F. (1991). An optimal design of CUSUM quality control charts. *Journal of Quality Technology, 23*, 279–286.
Gan, F. F. (1995). Joint monitoring of process mean and variance using exponentially weighted moving average control charts. *Technometrics, 37*(4), 446–453.

Gan, F. F., Ting, K. W., & Chang, T. C. (2004). Interval Charting Schemes for Joint Monitoring of Process Mean and Variance. *Quality and Reliability Engineering International, 20*, 291–303.
Garcia, M., & Cebrian, A. (1996). Repeated substitution method: The ratio estimator for the population variance. *Metrika, 43*(1), 101–105.
Grover, L. K., & Kaur, P. (2014). A generalized class of ratio type exponential estimators of population mean under linear transformation of auxiliary variable. *Communications in Statistics-Simulation and Computation, 43*(7), 1552–1574.
Haq, A. (2017a). New EWMA control charts for monitoring process dispersion using auxiliary information. *Quality and Reliability Engineering International, 33*(8), 2597–2614.
Haq, A. (2017b). A new maximum EWMA control chart for simultaneously monitoring process mean and dispersion using auxiliary information. *Quality and Reliability Engineering International, 33*(7), 1577–1587.
Haq, A. (2018). A new adaptive EWMA control chart using auxiliary information for monitoring the process mean. *Communications in Statistics - Theory and Methods, 47*(19), 4840–4858.
Haq, A., & Khoo, M. B. C. (2016). A new synthetic control chart for monitoring process mean using auxiliary information. *Journal of Statistical Computation and Simulation, 86*(15), 3068–3092.
Hinken, J. H., & Beller, T. (2006). Contactless Thickness Measurements of Glass Walls by Using Microwave Reflections. *Paper presented at the ECNDT Hochschule Magdeburg-Stendal (FH)*. Magdeburg, Germany: University of Applied Sciences.
Hussain, S., Song, L., Ahmad, S., & Riaz, M. (2019). New Interquartile Range EWMA Control Charts with Applications in Continuous Stirred Tank Rector Process. *Arabian Journal for Science and Engineering, 44*, 2467–2485.
Javaid, A., Noor-ul-Amin, M., & Hanif, M. (2020). A new Max-HEWMA control chart using auxiliary information. *Communications in Statistics - Simulation and Computation, 49*(5), 1285–1305. https://doi.org/10.1080/03610918.2018.1494282
Khoo, M. B. C., Teh, S., & Wu, Z. (2010). Monitoring process mean and variability with one double EWMA chart. *Communications in Statistics - Theory and Methods, 39*(20), 3678–3694.
Lee, H., Aslam, M., Shakeel, Q. U., Lee, W., & Jun, C. H. (2015). A control chart using an auxiliary variable and repetitive sampling for monitoring process mean. *Journal of Statistical Computation and Simulation, 85*(16), 3289–3296.
McCracken, A. K., & Chakraborti, S. (2013). Control Charts for Joint Monitoring of Mean and Variance: An Overview. *Quality Technology and Quantitative Management, 10*(1), 17–36.
Min'ko, N. I., & Nartsev, V. M. (2013). Factors Affecting the Strength of the Glass (Review). *Middle-East Journal of Scientific Research, 18*(11), 1616–1624.
Mughal, M. A., Azam, M., & Aslam, M. (2018). An EWMA-DiD Control Chart to Capture Small Shifts in the Process Average Using Auxiliary Information. *Technologies, 6*, 1–16.
Mukherjee, A., McCracken, A. K., & Chakraborti, S. (2015). Control Charts for Simultaneous Monitoring of Parameters of a Shifted Exponential Distribution. *Journal of Quality Technology, 47*(2), 176–192. https://doi.org/10.1080/00224065.2015.11918123
Noor-ul-Amin, M., Tariq, S., & Hanif, M. (2019). Control charts for simultaneously monitoring of process mean and coefficient of variation with and without auxiliary information. *Quality and Reliability Engineering International, 35*(8), 2639–2656.
Osei-Aning, R., Abbasi, S. A., & Riaz, M. (2017). Mixed EWMA-CUSUM and mixed CUSUM-EWMA modified control charts for monitoring first order autoregressive processes. *Quality Technology & Quantitative Management, 14*(4), 429–453.
Page, E. S. (1954). Continuous Inspection Schemes. *Biometrika, 41*, 100–115.
Quesenberry, C. P. (1995). On properties of Q charts variables. *Journal of Quality Technology, 27*(3), 184–203.
Riaz, M. (2008). Monitoring Process Mean Level using Auxiliary Information. *Statistica Neerlandica, 62*(4), 458–481.
Riaz, M., Mehmood, R., Ahmad, S., & Abbasi, S. A. (2013). On the Performance of Auxiliary based Control Charting under Normality and Nonnormality with Estimation Effects. *Quality and Reliability Engineering International, 29*(8), 1165–1179.
Roberts, S. W. (1959). Control Chart Tests Based on Geometric Moving Averages. *Technometrics, 1*(3), 239–250.
Sanusi, R. A., Abbas, N., & Riaz, M. (2017). On efficient CUSUM-type location control charts using auxiliary information. *Quality Technology & Quantitative Management, 15*(1), 87–105.
Sheu, S. H., Huang, C. J., & Hsu, T. S. (2012). Extended maximum generally weighted moving average control chart for monitoring process mean and variability. *Computers & Industrial Engineering, 62*(1), 216–225.
Shewhart, W. A. (1931). Economic control of quality manufactured product. from D. Van Nostrand(reprinted by the American Society for Quality Control in 1980, Milwaukee, WI).
Singh, H. P., & Solanki, R. S. (2013). A new procedure for variance estimation in simple random sampling using auxiliary information. *Statistical Papers, 54*(2), 479–497.
Sodipo, A. (2010). Difference-type and regression-type estimators for the population mean based on poststratification and subsampling of the nonrespondents. *European Journal of Scientific Research, 43*(4), 445–451.
Thaga, K. (2004). *Contributions to statistical process control tools* (PhD). The University of Manitoba.
Zaman, B., Abbas, N., Riaz, M., & Lee, M. H. (2016). Mixed CUSUM-EWMA chart for monitoring process dispersion. *The International Journal of Advanced Manufacturing Technology, 86*, 3025–3039.
Zaman, B., Riaz, M., Abbas, N., & Does, R. J. M. M. (2015). Mixed cumulative sum–exponentially weighted moving average control charts: An efficient way of monitoring process location. *Quality and Reliability Engineering International, 31*, 1407–1421.
Zaman, B., Riaz, M., & Lee, M. H. (2016). On the Performance of Control Charts for Simultaneous Monitoring of Location and Dispersion Parameters. *Quality and Reliability Engineering International, 33*(1), 37–57.
