# Supplementary material for: Improved adaptive EWMA control chart for process location with applications in groundwater physicochemical parameters and glass manufacturing industry
Source: PLoS One. 2022 Aug 22;17(8):e0272584. doi: 10.1371/journal.pone.0272584 (PMC9394848; doi:10.1371/journal.pone.0272584)
Supplement: S2 Data — (PDF) [file pone.0272584.s002.pdf]

Article

Transactions of the Institute of Measurement and Control
1–15
© The Author(s) 2016
Reprints and permissions:
sagepub.co.uk/journalsPermissions.nav
DOI: 10.1177/0142331215622248
tim.sagepub.com
SAGE

On the extended use of auxiliary information under skewnesss correction for process monitoring

Rashid Mehmood, Muhammad Riaz, Tahir Mahmood, Saddam Akbar Abbasi and Nasir Abbas

Abstract

In this article, we have extended the design structures of dual auxiliary information-based control charts under a variety of sampling strategies and runs rules schemes. We have considered the cases of known and unknown skewed distributions by using the skewness correction (SC) method. The design structures under the skewness correction method are based on the degree of skewness of the study variable, amount of correlation between study variable and auxiliary variable, and sample size. We have investigated the performance of the developed structures in terms of probability of signals, false alarm rate and average run length by considering the symmetrical distribution, skewed distributions, heavy tailed distributions and contamination environments. Outcomes of the current article showed that control charts based on extreme ranked set strategies have higher probability of detecting an out-of-control signal and are comparatively more robust than other control charts, especially for known distributions. Furthermore, control charts for unknown skewed process distributions under extreme ranked set strategies are relatively more robust for a small sample size, followed by other ranked set strategies-based control charts for a large sample size. Moreover, we have included a real-life example for the monitoring of ground water variables to highlight the application of our proposals.

Keywords

Auxiliary information, contamination, control chart, distributions, false alarm rate, probability of signals, robustness, runs rules

Introduction

Statistical process control (SPC) consists of tools that are used to monitor a special cause of variation in the process parameters (location and dispersion), and the most important one is quality control chart. A control chart is a graphical display for monitoring a process characteristic of interest and has wide applications in various disciplines such as industrial processes, medical sciences and environmental sciences. The idea of quality control charts was initiated by Walter A. Shewhart in 1931. The most commonly used Shewhart-type control charts are $\bar{X}$, R, S, S^2, the design structures of which mainly depend on a study variable. Sometimes one may have extra (auxiliary) information, which is particularly or completely known and correlated with the study variable. For such situations, auxiliary information has been incorporated with the design structures of Shewhart-type control charts in the form of either ranking the units of interest or estimation of parameters, or both (cf. Abbasi and Riaz, 2015; Abujiya and Muttlak, 2004; Mehmood et al., 2013; Muttlak and Al-Sabah, 2003; Riaz, 2008).

One of the popular mechanisms for utilizing the auxiliary information is ranking the units of interest, which was given by Muttlak and Al-Sabah (2003). They developed the location control charts under single ranked set strategies, which include ranked set sampling (RSS), median ranked set sampling (MRSS) and extreme ranked set sampling (ERSS). Abujiya and Muttlak (2004) then extended the idea of single ranked set strategies to double ranked set strategies such as double ranked set sampling (DRSS) and double extreme ranked set sampling (DERSS). They demonstrated that control charts based on double ranked set strategies are more efficient than control charts based on single ranked set strategies. In addition, control charts proposed by Muttlak and Al-Sabah (2003), and Abujiya and Muttlak (2004) were based on one point decision rule (process can be declared out-of-control if single plotting statistic outside the control limits), which usually considered less efficient for the detection of smaller shifts. The limitation of one point decision rule is discussed in many studies such as Montgomery (2009) and Riaz et al. (2011). Later on, Mehmood et al. (2013) attached more runs rules with the different ranked set strategies-based structures and improved their detection ability towards smaller shifts. One recent application of a single use of auxiliary information to design the dispersion control charts under

Department of Mathematics and Statistics, King Fahad University of Petroleum and Minerals, Dhahran 31261, Saudi Arabia

Corresponding author:
Muhammad Riaz, Department of Mathematics and Statistics, King Fahad University of Petroleum and Minerals, Hamza Street, Dhahran 31261, Saudi Arabia.
Email: riaz76qau@yahoo.com

Downloaded from tim.sagepub.com at UNIV CALIFORNIA SAN DIEGO on March 15, 2016

various ranked set strategies may be seen in Abujiya et al. (2015).

Another popular mechanism for utilizing the auxiliary information has been seen at the estimation stage to design a control chart. Riaz (2008) considered the auxiliary information at estimation stage and proposed a control chart for the monitoring of location parameter. Similar usage of auxiliary information has seen in the articles by Riaz et al. (2014) and Ahmed et al. (2014).

Yu and Lam (1997) and Muttlak (2001) recommended the use of auxiliary information for dual purposes instead of using it either for ranking of units or estimation. For this, they suggested regression type estimators under RSS (cf. Yu and Lam, 1997), MRSS and ERSS (cf. Muttlak, 2001). Moreover, one recent application of the dual use of auxiliary information has seen for the design of structures of control charts. Abbasi and Riaz (2015) promoted the idea of dual use of auxiliary information in control charts. They proposed location control charts based on single ranked set strategies (include RSS, ERSS and MRSS) and the one point decision rule.

The aforementioned studies (e.g. Abbasi and Riaz, 2015; Abujiya et al., 2015) were designed for known process distribution (and only limited to such processes that follow normal distribution) and performance of the control charts was investigated only under the uncontaminated scenarios. It is important to mention here that in many practical situations the process distribution may be either non-normal or unknown (cf. Chan and Cui, 2003; Schoonhoven and Does, 2011). Moreover, the performance of a control chart under a sampling strategy may be varied due to the violations of distributional assumption and contaminated scenarios (Shoonhoven et al., 2011; Tatum, 1997). One may refer to Sindhu et al. (2015), Riaz and Ali (2015) and Ahmad et al. (2013) and the references therein for more relevant work on process monitoring.

In this paper, we mainly intend to propose more generalized location control charts (based on dual use of auxiliary information) for known process distributions (following Abbasi and Riaz, 2015) as well as for unknown skewed process distributions (following Chan and Cui, 2003). To design the said control charts, we consider a variety of distributions, the existing regression estimator under single ranked set strategies, and propose a regression estimator under double ranked set strategies and runs rules. Furthermore, the skewness correction method is used to design the control charts for unknown skewed distributions (Chan and Cui, 2003). The design structure based on the skewness correction method is developed by taking into account the skewness of the study variable Y, the amounts of correlation between study variable Y and auxiliary variable X, and the sample size, instead of a strict assumption of normality (cf. Abbasi and Riaz, 2015). The performance of the proposed control charts are investigated through probability of signals and false alarm rate by considering the skewed distributions, heavy tailed distributions and their contaminated cases.

The rest of the article is as follows: in the next section, we explain the regression estimator under different sampling strategies. Then, we propose a set of location control charts for known process distributions and unknown skewed process distributions separately. We investigate the performance of proposed charts in terms of the probability of signals (also termed as power), false alarm rate and average run length, and elaborate results and discussion based on the performance measures. An application of the proposed charts in groundwater monitoring is presented and finally concluding remarks for the whole study are provided.

Regression estimators under different sampling strategies

In the subsequent sections, we illustrate regression estimators under different ranked set strategies, which utilize the auxiliary information for ranking the units as well as for the estimation of parameter. Firstly, we explain regression estimators under single ranked set strategies, which were introduced by Yu and Lam (1997), and Muttlak (2001). Then, on the same guideline of Yu and Lam (1997) and Muttlak (2001), we propose regression estimators under double ranked set strategies. The single ranked set strategies include RSS and ERSS, whereas double ranked set strategies cover DRSS and DERSS.

Regression estimator under ranked set sampling (RSS)

The procedure of gathering a sample under RSS is provided in the following steps:

Step 1: Select n^2 random sample from the population of interest, divide them into n sets each of size n.
Step 2: Sort n sets with respect to X.
Step 3: Select a unit from the first set that is associated with the smallest measured value of X. Similarly, select a unit from the second set associated with the second smallest value of X.
Step 4: Continue the following mechanism until the nth unit is selected associated with the largest value of X. In this way, an RSS sample of size n is selected.

In order to collect r ranked set samples of size n, repeat the above mentioned procedure r times, and finally, nr data values of actual measurements are gathered.

Let $\left(Y_{[i,rss],j}, X_{(i,rss),j}\right)$, where $i = 1,2,3,\ldots,n$ and $j = 1,2,3,\ldots,r$ denote jth ranked set sample of size n. Then a regression estimator (Muttlak, 2001; Yu and Lam, 1997) for the jth sample of size n is given below:

$$V_{RSS,j} = \bar{Y}_{RSS,j} + \left[r_{YX_{RSS,j}} \frac{s_{Y_{RSS,j}}}{s_{X_{RSS,j}}} \right] \left[\mu_X - \bar{X}_{RSS,j} \right],\ j = 1,2,3,\ \ldots,r \tag{1}$$

Variance of the above estimator is given as:

$$\sigma^2_{V_{RSS,j}} = \frac{\sigma^2_Y}{n}\left(1-\rho^2_{YX}\right)\left[1 + E\left(\frac{\bar{Z}^2_{RSS}}{S^2_{Z_{RSS}}}\right)\right], \tag{2}$$

where $\bar{Y}_{RSS,j}$ and $\bar{X}_{RSS,j}$ are sample means for the jth RSS sample of size n. Moreover, other quantities are defined as:

Downloaded from tim.sagepub.com at UNIV CALIFORNIA SAN DIEGO on March 15, 2016

$$\bar{Z}_{RSS} = \frac{1}{n}\sum_{i=1}^{n} Z_{(i,rss),j}, S^2_{Z_{RSS}} = \frac{1}{n}\sum_{i=1}^{n}\left[Z_{(i,rss),j} - \bar{Z}_{RSS}\right],$$
$$Z_{(i,rss),j} = \frac{X_{(i,rss),j-\mu_X}}{\sigma_X},$$

Regression estimator under extreme ranked set sampling (ERSS)

In ERSS, steps 1, 2 and 4 remain the same as in the case of RSS. The step 3 depends on the sample size n. So, if n is odd, from first $(\frac{n-1}{2})$ sets select the units for actual measurement of Y that are associated with the smallest measured value of X; from the other $(\frac{n-1}{2})$ sets select the units that are associated with largest measured value of X; and from the remaining set pick the unit at the median position. In the case where n is even, select units from $(\frac{n}{2})$ sets that are associated with the smallest measured value of X and from the other $(\frac{n}{2})$ sets that are associated with largest measured value of X. In this way, an ERSS-based sample is obtained.

Let $\left(Y_{[i,erss],j}, X_{(i,erss),j}\right)$, where $i = 1,2,3,\ldots,n$ and $j = 1,2,3,\ldots,r$ denote the jth ranked set sample of size n. Then regression estimators (Muttlak, 2001; Yu and Lam, 1997) for the jth sample of size n are given below:

$$V_{ERSS,j} = \bar{Y}_{ERSS,j} + \left(r_{YX_{ERSS,j}}\frac{s_{Y_{ERSS,j}}}{s_{X_{ERSS,j}}}\right)\left[\mu_X - \bar{X}_{ERSS,j}\right], \quad j = 1,2,3,\ldots,r \tag{3}$$

Variance of the above estimators is given below:

$$\sigma^2_{V_{ERSS,j}} = \frac{\sigma^2_Y}{n}\left(1-\rho^2_{YX}\right)\left[1 + E\left(\frac{\bar{Z}^2_{ERSS}}{S^2_{Z_{ERSS}}}\right)\right], \tag{4}$$

where $\bar{Y}_{ERSS,j}$ and $\bar{X}_{ERSS,j}$ are sample means for the jth ERSS sample of size n. Moreover, other quantities are defined as:

$$\bar{Z}_{ERSS} = \frac{1}{n}\sum_{i=1}^{n} Z_{(i,erss),j}, S^2_{Z_{ERSS}} = \frac{1}{n}\sum_{i=1}^{n}\left[Z_{(i,erss),j} - \bar{Z}_{ERSS}\right],$$
$$Z_{(i,erss),j} = \frac{X_{(i,erss),j-\mu_X}}{\sigma_X}$$

Regression estimators under double ranked set sampling (DRSS) and double extreme ranked set sampling (DERSS)

The procedures of DRSS and DERSS are explained as follows:

Step 1: Select n random samples of size n^2.
Step 2: Apply the RSS procedure on each random sample, which results in n ranked set samples of size n.
Step 3: Again apply the RSS procedure on the resulted ranked set samples of size n.

Finally, the following procedure provide us double ranked set sample of size n. Similarly, one can proceed for DERSS.

On the same guidelines of Yu and Lam (1997) and Muttlak (2001), we propose regression estimators under DRSS and DERSS.

Let $\left(Y_{[i,drss],j}, X_{(i,drss),j}\right)$, and $\left(Y_{[i,derss],j}, X_{(i,derss),j}\right)$, where $i = 1,2,3,\ldots,n$ and $j = 1,2,3,\ldots,r$, denote the jth sample of size n, collected using the procedure of DRSS and DERSS, respectively. Then, regression estimators for the jth DRSS and DERSS of size n are as follows:

$$V_{DRSS,j} = \bar{Y}_{DRSS,j} + \left[r_{YX_{DRSS,j}}\frac{s_{Y_{DRSS,j}}}{s_{X_{DRSS,j}}}\right]\left[\mu_X - \bar{X}_{DRSS,j}\right], \quad j = 1,2,3,\ldots,r \tag{5}$$

$$V_{DERSS,j} = \bar{Y}_{DERSS,j} + \left[r_{YX_{DERSS,j}}\frac{s_{Y_{DERSS,j}}}{s_{X_{DERSS,j}}}\right]\left[\mu_X - \bar{X}_{DERSS,j}\right], \quad j = 1,2,3,\ldots,r \tag{6}$$

Variances of the above estimators are given as:

$$\sigma^2_{V_{DRSS,j}} = \frac{\sigma^2_Y}{n}\left(1-\rho^2_{YX}\right)\left[1 + E\left(\frac{\bar{Z}^2_{DRSS}}{S^2_{Z_{DRSS}}}\right)\right], \tag{7}$$

$$\sigma^2_{V_{DERSS,j}} = \frac{\sigma^2_Y}{n}\left(1-\rho^2_{YX}\right)\left[1 + E\left(\frac{\bar{Z}^2_{DERSS}}{S^2_{Z_{DERSS}}}\right)\right], \tag{8}$$

where $\bar{Y}_{DRSS,j}, \bar{Y}_{DERSS,j}\bar{X}_{DRSS,j}$ and $\bar{X}_{DERSS,j}$ are sample means for the jth DRSS and DERSS samples of size n, like in the single ranked set strategies. Moreover, other quantities are defined as:

$$\bar{Z}_{DRSS} = \frac{1}{n}\sum\nolimits_{i=1}^{n} Z_{(i,drss),j}, S^2_{Z_{DRSS}} = \frac{1}{n}\sum\nolimits_{i=1}^{n}\left[Z_{(i,drss),j} - \bar{Z}_{DRSS}\right],$$

$$Z_{(i,drss),j} = \frac{X_{(i,drss),j-\mu_X}}{\sigma_X}, \bar{Z}_{DERSS} = \frac{1}{n}\sum\nolimits_{i=1}^{n} Z_{(i,drss),j},$$

$$S^2_{Z_{DERSS}} = \frac{1}{n}\sum\nolimits_{i=1}^{n}\left[Z_{(i,derss),j} - \bar{Z}_{DERSS}\right], Z_{(i,derss),j} = \frac{X_{(i,derss),j-\mu_X}}{\sigma_X}$$

Proposed location control charts

In this section, we develop two design structures of location control charts (based on dual auxiliary information) using the regression estimators (see previously). For more involvement, control charts for a known process distribution and unknown skewed process distribution are denoted by $V_{(G,D)}$ and $V_{(G,SC)}$, respectively, where subscript G denotes a sampling strategy, D refers to a bivariate distribution and SC represents the skewness correction method.

Design structure when process distribution is known

Let $V_{G,1}, V_{G,2,}, \ldots, V_{G,r}$, denote r plotting statistics based on the samples of size n that are collected using any of the sampling strategy G (RSS, ERSS, DRSS and DERSS) under consideration with a known statistical distribution D of process characteristics [such as bivariate normal (BN), bivariate t

Downloaded from tim.sagepub.com at UNIV CALIFORNIA SAN DIEGO on March 15, 2016

(BT) and bivariate lognormal (BLN)]. The design structure of the proposed control charts are:

$$LCL_{V_{(G,D)}} = \mu_Y + L_{(G,D,n,\rho_{YX},\frac{\alpha}{2})}\sigma_Y, \quad \text{and } UCL_{V_{(G,D)}} = \mu_Y + L_{(G,D,n,\rho_{YX},1-\frac{\alpha}{2})}\sigma_Y \tag{9}$$

where μ_Y and σ_Y are the population mean and standard deviation of the variable of interest Y,$L_{(G,D,n,\rho_{YX},\frac{p}{2})}$ and $L_{(G,D,n,\rho_{YX},1-\frac{p}{2})}$ are control limit factors depending on sampling strategy G, distribution D, sample size n and correlation ρ_{YX} between Y and X. The structure given in Equation (9) is based on the one point decision rule. In order to make a design structure (given in Equation 1) that is more sensitive for smaller and moderate shifts, we have defined and attached more runs rules (following Mehmood et al., 2013, 2014; Riaz et al., 2011): "Process can be declared out-of-control, if at least $k-m$ values of the test statistic ($V_{G,j}, j=1,2,3$) out of k consecutive values of test statistic either exceed the lower control limit $LCL_{V_{(G,D)}}$ or upper control limit $UCL_{V_{(G,D)}}$." The following term can be represented as $k-m|k$ with the condition that $0 \le m \le k-1$.

$$LCL_{V_{(G,D)}} = \mu_Y + L_{(G,D,n,k-m,k,\rho_{YX},\frac{p}{2})}\sigma_Y, \quad UCL_{V_{(G,D)}} = \mu_Y + L_{(G,D,n,k-m,k,\rho_{YX},1-\frac{p}{2})}\sigma_Y \tag{10}$$

where $k-m$ denotes the decision observations in order to declare a process out-of-control, k is the total observations in a given rule and p is the probability of a single point going outside the signalling limits. The value of p can be obtained by solving the expression $\alpha = \sum_{k-m \le k} \frac{k!}{(k-m)!m!} p^{k-m}(1-p)^m$, where $0 \le m \le k-1$, for a given value of $k-m$, k and false alarm rate (α). The remaining quantities have been defined earlier. It is important to mention here that $V_{(RSS,BN)}$ and $V_{(ERSS,BN)}$ control charts are special cases of existing control charts (Abbasi and Riaz, 2015) when $k-m=1$ and $k=1$.

Furthermore, control limits factors are derived through Monte Carlo simulation. Before illustrating the computation procedure, it is important to mention here that for BLN distribution (which is a case of skewed distribution), we consider $Y = lnQ$ and $Y = lnW$ with means and variances $\mu_Y = \ln\left(\mu_Q\right) - \left(\frac{\sigma_Q^2}{2}\right)$, $\mu_X = ln(\mu_W) - \left(\frac{\sigma_W^2}{2}\right)$, $\sigma_Y^2 = ln\left(1 + \frac{\sigma_Q^2}{\mu_Q^2}\right)$ and $\sigma_X^2 = ln\left(1 + \frac{\sigma_W^2}{\mu_W^2}\right)$, where (Q, W) follows BLN distribution with means μ_Q and μ_W, and variances σ_Q^2 and σ_W^2, respectively (for some details see the Appendix). Details of the following transformation are provided by Stedinger et al. (1993) and Yerel and Konuk (2009). Moreover, it is helpful to bring the non-linear random variable into linear form. Assumption of linearity of a variable is usually considered important when using a difference estimator and regression estimator. Yu and Lam (1997) and Muttlak (2001) concluded that regression estimators work efficiently when (Y, X) is linear. They also concluded some kind of transformation can be used for using the regression estimator when the variables under consideration are non-linear.

Then, we consider different value of design parameters $(G, Dk-m, k, n, \rho_{YX})$, random samples are generated from a given bivariate distribution D ($\mu_Y = 0$, $\mu_X = 0$, $\sigma_Y^2 = 1$, $\sigma_X^2 = 1$ and $v = 30$) and the charting statistic are calculated 10^5 times. Finally, the control limits factors (L) are obtained by taking the $\left(1-\frac{p}{2}\right)$th and $\left(\frac{p}{2}\right)$th quantiles of the sampling distribution of test statistic for given values of $G, D, k-m, k, n,$ and ρ_{YX}. Moreover, in this study we have tabulated control limits factors for selective choices of control charts in the form of Tables 1 and 2 under varying values of design parameters $(G, D, k-m, k, n, \rho_{YX})$ at $\alpha = 0.0027$. Similarly, control limits factors can be derived for the other choice of design parameters and α.

Design structure for unknown process distribution under skewness correction

Let $V_{G,1}, V_{G,2}, \ldots, V_{G,r}$, denote r plotting statistics based on the samples of size n that are collected using any of the sampling strategy G (include RSS, ERSS, DRSS and DERSS) under consideration with known values of skewness of the study variable $Y(k_3)$, means ($\mu_Y$andμ_X), standard deviations (σ_Y andσ_X) and correlation (ρ_{YX}) between the study variable Y and auxiliary variable X. The proposed skewness adjustment control charts for unknown skewed distribution are:

$$LCL_{V_{(G,SC)}} = \mu_Y + \left[Z_{(\frac{\alpha}{2})}k_2 + c_4^*\right]\sigma_Y, \text{and } UCL_{V_{(G,SC)}} = \mu_Y + \left[Z_{(1-\frac{\alpha}{2})}k_2 + c_4^*\right]\sigma_Y \tag{11}$$

where $c_4^* = \frac{\left(Z^2_{\left(\frac{\alpha}{2}\right)} - 1\right)k_3(V)k_2}{6 \over 1+0.2k_3(V)} = \frac{\left(Z^2_{\left(1-\frac{\alpha}{2}\right)} - 1\right)k_3(V)k_2}{6 \over 1+0.2k_3(V)}$ is the skewness adjustment factor, $Z_{(\frac{\alpha}{2})}$ and $Z_{(1-\frac{\alpha}{2})}$ are $(\frac{\alpha}{2})$ th and $(1-\frac{\alpha}{2})$ th quantiles of the standard normal distribution for a given false alarm rate α, $k_3(V)$ refers to skewness of the statistics $(V_{G,j}, j=1,2,\ldots,r)$ and k_2 is the ratio of standard deviation of statistics $V_{G,j}$ and standard deviation of the study variable σ_Y, i.e. $\frac{\sigma_{V_{G,j}}}{\sigma_Y} = \sqrt{\frac{1}{n}(1-\rho_{YX}^2)\left[1 + E\left(\frac{\bar{Z}_G^2}{S_{Z_G}^2}\right)\right]}$. We have computed k_2 through Monte Carlo simulation described thus: for a given value of n, ρ_{YX} and G, random samples are generated (10^5 times) from any of the bivariate distribution with no restriction on the choice of parameters. Compute the statistics $(V_{G,j}, j=1,2,\ldots 10^5)$ and finally the ratio of the standard deviations of the computed statistic and the study variable σ_Y is our desired k_2. We have provided k_2 for some selective choices n and ρ_{YX} with varying sampling strategies (cf. Table 3).

An alternative approach of representing the Equation (11) is as follows:

$$LCL_{V_{(G,SC)}} = \mu_Y + B_L^*\sigma_Y, \text{and } UCL_{V_{(G,SC)}} = \mu_Y + B_U^*\sigma_Y \tag{12}$$

where $B_L^* = \left[Z_{(\frac{\alpha}{2})}k_2 + c_4^*\right]$ and $B_U^* = \left[Z_{(1-\frac{\alpha}{2})}k_2 + c_4^*\right]$ are the control limits factors.

After defining the control charting structures (Equations 11 and 12) for unknown skewed distributions, we have calculated the skewness adjustment factor c_4^* following Chan and Cui (2003) for different choices of skewness (which depend on

Downloaded from tim.sagepub.com at UNIV CALIFORNIA SAN DIEGO on March 15, 2016

Table 1. Control limits factors of $\boldsymbol{V}_{(ERSS,\,BN)}$ control charts with different choices of $\boldsymbol{k-m}, k, n$ **and** $\boldsymbol{\rho_{YX}}$ at $\alpha = 0.0027$.

ρ_{YX}	n	Control limits factors	$\boldsymbol{k-m \vert k}$					
			1|1	2|3	2|4	9|9	8|9	7|9
0.30	4	$L_{(G,D,k-m,k,n,\rho_{YX},\frac{p}{2})}$	−1.57	−1.1022	−1.1719	−0.3233	−0.4383	−0.5441
		$L_{(G,D,k-m,k,n,\rho_{YX}1-,\frac{p}{2})}$	1.5664	1.0933	1.1627	0.3239	0.4374	0.5418
	5	$L_{(G,D,k-m,k,n,\rho_{YX},\frac{p}{2})}$	−1.4365	−1.0023	−1.0665	−0.2918	−0.3959	−0.4908
		$L_{(G,D,k-m,k,n,\rho_{YX}1-,\frac{p}{2})}$	1.4355	1.0036	1.069	0.297	0.4014	0.4972
	6	$L_{(G,D,k-m,k,n,\rho_{YX},\frac{p}{2})}$	−1.0757	−0.7554	−0.8042	−0.2242	−0.3034	−0.3764
		$L_{(G,D,k-m,k,n,\rho_{YX}1-,\frac{p}{2})}$	1.0901	0.763	0.8125	0.2236	0.3042	0.3773
	8	$L_{(G,D,k-m,k,n,\rho_{YX},\frac{p}{2})}$	−0.7189	−0.5018	−0.5355	−0.1489	−0.201	−0.2495
		$L_{(G,D,k-m,k,n,\rho_{YX}1-,\frac{p}{2})}$	0.712	0.5038	0.5356	0.1474	0.2	0.2477
0.50	4	$L_{(G,D,k-m,k,n,\rho_{YX},\frac{p}{2})}$	−1.3395	−0.9594	−1.0185	−0.285	−0.3854	−0.4775
		$L_{(G,D,k-m,k,n,\rho_{YX}1-,\frac{p}{2})}$	1.3407	0.9575	1.015	0.2851	0.3859	0.4767
	5	$L_{(G,D,k-m,k,n,\rho_{YX},\frac{p}{2})}$	−1.2165	−0.8704	−0.9259	−0.2585	−0.3496	−0.434
		$L_{(G,D,k-m,k,n,\rho_{YX}1-,\frac{p}{2})}$	1.2195	0.8732	0.9253	0.2567	0.3483	0.4331
	6	$L_{(G,D,k-m,k,n,\rho_{YX},\frac{p}{2})}$	−0.9334	−0.6672	−0.7094	−0.1971	−0.2675	−0.3313
		$L_{(G,D,k-m,k,n,\rho_{YX}1-,\frac{p}{2})}$	0.9266	0.6635	0.7044	0.1984	0.268	0.332
	8	$L_{(G,D,k-m,k,n,\rho_{YX},\frac{p}{2})}$	−0.6168	−0.4399	−0.4681	−0.1307	−0.1769	−0.2193
		$L_{(G,D,k-m,k,n,\rho_{YX}1-,\frac{p}{2})}$	0.6091	0.4402	0.4683	0.1305	0.1769	0.2189
0.75	4	$L_{(G,D,k-m,k,n,\rho_{YX},\frac{p}{2})}$	−1.1926	−0.8613	−0.9116	−0.2553	−0.3452	−0.428
		$L_{(G,D,k-m,k,n,\rho_{YX}1-,\frac{p}{2})}$	1.2093	0.8598	0.9129	0.2586	0.3498	0.433
	5	$L_{(G,D,k-m,k,n,\rho_{YX},\frac{p}{2})}$	−1.0781	−0.7783	−0.8274	−0.2304	−0.313	−0.3882
		$L_{(G,D,k-m,k,n,\rho_{YX}1-,\frac{p}{2})}$	1.0814	0.7776	0.8271	0.2338	0.3158	0.3909
	6	$L_{(G,D,k-m,k,n,\rho_{YX},\frac{p}{2})}$	−0.8304	−0.5934	−0.6315	−0.1775	−0.2394	−0.2967
		$L_{(G,D,k-m,k,n,\rho_{YX}1-,\frac{p}{2})}$	0.8326	0.5966	0.6332	0.1763	0.2397	0.2977
	8	$L_{(G,D,k-m,k,n,\rho_{YX},\frac{p}{2})}$	−0.5412	−0.3932	−0.4172	−0.1168	−0.1575	−0.1955
		$L_{(G,D,k-m,k,n,\rho_{YX}1-,\frac{p}{2})}$	0.5477	0.3918	0.4165	0.1171	0.1584	0.1963
0.90	4	$L_{(G,D,k-m,k,n,\rho_{YX},\frac{p}{2})}$	−1.0184	−0.7336	−0.7781	−0.2198	−0.2965	−0.3661
		$L_{(G,D,k-m,k,n,\rho_{YX}1-,\frac{p}{2})}$	1.02	0.7366	0.7826	0.2211	0.2975	0.3682
	5	$L_{(G,D,k-m,k,n,\rho_{YX},\frac{p}{2})}$	−0.9307	−0.6683	−0.7108	−0.1996	−0.2697	−0.3344
		$L_{(G,D,k-m,k,n,\rho_{YX}1-,\frac{p}{2})}$	0.9255	0.6715	0.7157	0.2003	0.2709	0.3358
	6	$L_{(G,D,k-m,k,n,\rho_{YX},\frac{p}{2})}$	−0.7053	−0.5122	−0.5449	−0.1518	−0.2056	−0.255
		$L_{(G,D,k-m,k,n,\rho_{YX}1-,\frac{p}{2})}$	0.7092	0.5106	0.5418	0.152	0.2059	0.2562
	8	$L_{(G,D,k-m,k,n,\rho_{YX},\frac{p}{2})}$	−0.4639	−0.3364	−0.3564	−0.1011	−0.1368	−0.1694
		$L_{(G,D,k-m,k,n,\rho_{YX}1-,\frac{p}{2})}$	0.4664	0.3378	0.3587	0.1008	0.1361	0.168

the value of parameters of a given distribution), n, ρ_{YX} and sampling strategy G at $\alpha = 0.0027$. The results of the following constant are tabulated in Table 4. We also provided the control limits coefficients (B_L^*, B_U^*) in Table 5. The theoretical justifications of control limits coefficients are provided in the Appendix.

Performance evaluation of proposed control charts

Previously, we have developed two control charting structures of $V_{(G,D)}$ and $V_{(G,SC)}$ control charts, which are given in the form of Equations (10)–(12). In the following section, we investigate the performance of $V_{(G,D)}$ and $V_{(G,SC)}$ control charts. For the said purposes, we use the probability of signals, false alarm rate and average run length as the performance measures for evaluating the performance of $V_{(G,D)}$ and $V_{(G,SC)}$ control charts. The probability of signals is the detection probability of a given control chart when the process actually out-of-control. The false alarm rate is the probability of process breaching outside the control limits $\left(LCL_{V_{(G,SC)}}, UCL_{V_{(G,SC)}}\right)$ when it is in fact in control. The average run length (ARL) is defined as: 'the average number of sample points that must be plotted before a point indicates an out-of-control (see Montgomery (2009))'. In more detail, if in fact a process is in control, then ARL is denoted by ARL_0, and if a process is out of control, then ARL is expressed by ARL_1. The ARL_0 and ARL_1 can easily be computed through false alarm rate and probability of signals. If the process measured observations are independent, then for any Shewhart control charts, ARL_0 and ARL_1 can be computed through the following relations (for single point decision rule): $ARL_o = \frac{1}{\text{false alarm rate}}$ and $ARL_1 = \frac{1}{\text{probability to signals}}$.

Performance evaluation of the $V_{(G,D)}$ control charts

In order to compute the probability of signals and ARL_1, we assume the shift δ occurs in the process parameter of the variable of interest μ_Y. The shift in the process parameter of the variable of interest Y is defined as: $\mu_{Y(\delta)} = \mu_Y + \delta\sigma_Y$. Moreover, $\delta = 0$ implies that no shift occurs in the process parameter of the variable of interest and the process behaving

Downloaded from tim.sagepub.com at UNIV CALIFORNIA SAN DIEGO on March 15, 2016

Table 2. Control limits factors of $V_{(ERSS, BT)}$ control charts with different choices of $k-m, k, n$ and ρ_{YX} at $\alpha = 0.0027$.

ρ_{YX}	n	Control limits factors	$k-m\|k$					
			1|1	2|3	2|4	9|9	8|9	7|9
0.30	4	$L_{(G,D,k-m,k,n,\rho_{YX},\frac{\alpha}{2})}$	−1.1022	−1.1719	−0.3233	−0.4383	−0.5441	−1.1022
		$L_{(G,D,k-m,k,n,\rho_{YX},1-\frac{\alpha}{2})}$	1.0933	1.1627	0.3239	0.4374	0.5418	1.0933
	5	$L_{(G,D,k-m,k,n,\rho_{YX},\frac{\alpha}{2})}$	−1.0023	−1.0665	−0.2918	−0.3959	−0.4908	−1.0023
		$L_{(G,D,k-m,k,n,\rho_{YX},1-\frac{\alpha}{2})}$	1.0036	1.069	0.297	0.4014	0.4972	1.0036
	6	$L_{(G,D,k-m,k,n,\rho_{YX},\frac{\alpha}{2})}$	−0.7554	−0.8042	−0.2242	−0.3034	−0.3764	−0.7554
		$L_{(G,D,k-m,k,n,\rho_{YX},1-\frac{\alpha}{2})}$	0.763	0.8125	0.2236	0.3042	0.3773	0.763
	8	$L_{(G,D,k-m,k,n,\rho_{YX},\frac{\alpha}{2})}$	−0.5018	−0.5355	−0.1489	−0.201	−0.2495	−0.5018
		$L_{(G,D,k-m,k,n,\rho_{YX},1-\frac{\alpha}{2})}$	0.5038	0.5356	0.1474	0.2	0.2477	0.5038
0.50	4	$L_{(G,D,k-m,k,n,\rho_{YX},\frac{\alpha}{2})}$	−0.9594	−1.0185	−0.285	−0.3854	−0.4775	−0.9594
		$L_{(G,D,k-m,k,n,\rho_{YX},1-\frac{\alpha}{2})}$	0.9575	1.015	0.2851	0.3859	0.4767	0.9575
	5	$L_{(G,D,k-m,k,n,\rho_{YX},\frac{\alpha}{2})}$	−0.8704	−0.9259	−0.2585	−0.3496	−0.434	−0.8704
		$L_{(G,D,k-m,k,n,\rho_{YX},1-\frac{\alpha}{2})}$	0.8732	0.9253	0.2567	0.3483	0.4331	0.8732
	6	$L_{(G,D,k-m,k,n,\rho_{YX},\frac{\alpha}{2})}$	−0.6672	−0.7094	−0.1971	−0.2675	−0.3313	−0.6672
		$L_{(G,D,k-m,k,n,\rho_{YX},1-\frac{\alpha}{2})}$	0.6635	0.7044	0.1984	0.268	0.332	0.6635
	8	$L_{(G,D,k-m,k,n,\rho_{YX},\frac{\alpha}{2})}$	−0.4399	−0.4681	−0.1307	−0.1769	−0.2193	−0.4399
		$L_{(G,D,k-m,k,n,\rho_{YX},1-\frac{\alpha}{2})}$	0.4402	0.4683	0.1305	0.1769	0.2189	0.4402
0.75	4	$L_{(G,D,k-m,k,n,\rho_{YX},\frac{\alpha}{2})}$	−0.8613	−0.9116	−0.2553	−0.3452	−0.428	−0.8613
		$L_{(G,D,k-m,k,n,\rho_{YX},1-\frac{\alpha}{2})}$	0.8598	0.9129	0.2586	0.3498	0.433	0.8598
	5	$L_{(G,D,k-m,k,n,\rho_{YX},\frac{\alpha}{2})}$	−0.7783	−0.8274	−0.2304	−0.313	−0.3882	−0.7783
		$L_{(G,D,k-m,k,n,\rho_{YX},1-\frac{\alpha}{2})}$	0.7776	0.8271	0.2338	0.3158	0.3909	0.7776
	6	$L_{(G,D,k-m,k,n,\rho_{YX},\frac{\alpha}{2})}$	−0.5934	−0.6315	−0.1775	−0.2394	−0.2967	−0.5934
		$L_{(G,D,k-m,k,n,\rho_{YX},1-\frac{\alpha}{2})}$	0.5966	0.6332	0.1763	0.2397	0.2977	0.5966
	8	$L_{(G,D,k-m,k,n,\rho_{YX},\frac{\alpha}{2})}$	−0.3932	−0.4172	−0.1168	−0.1575	−0.1955	−0.3932
		$L_{(G,D,k-m,k,n,\rho_{YX},1-\frac{\alpha}{2})}$	0.3918	0.4165	0.1171	0.1584	0.1963	0.3918
0.90	4	$L_{(G,D,k-m,k,n,\rho_{YX},\frac{\alpha}{2})}$	−0.7336	−0.7781	−0.2198	−0.2965	−0.3661	−0.7336
		$L_{(G,D,k-m,k,n,\rho_{YX},1-\frac{\alpha}{2})}$	0.7366	0.7826	0.2211	0.2975	0.3682	0.7366
	5	$L_{(G,D,k-m,k,n,\rho_{YX},\frac{\alpha}{2})}$	−0.6683	−0.7108	−0.1996	−0.2697	−0.3344	−0.6683
		$L_{(G,D,k-m,k,n,\rho_{YX},1-\frac{\alpha}{2})}$	0.6715	0.7157	0.2003	0.2709	0.3358	0.6715
	6	$L_{(G,D,k-m,k,n,\rho_{YX},\frac{\alpha}{2})}$	−0.5122	−0.5449	−0.1518	−0.2056	−0.255	−0.5122
		$L_{(G,D,k-m,k,n,\rho_{YX},1-\frac{\alpha}{2})}$	0.5106	0.5418	0.152	0.2059	0.2562	0.5106
	8	$L_{(G,D,k-m,k,n,\rho_{YX},\frac{\alpha}{2})}$	−0.3364	−0.3564	−0.1011	−0.1368	−0.1694	−0.3364
		$L_{(G,D,k-m,k,n,\rho_{YX},1-\frac{\alpha}{2})}$	0.3378	0.3587	0.1008	0.1361	0.168	0.3378

Table 3. K_2 with varying value of n, ρ_{YX} and G.

n	ρ_{YX}	G			
		RSS	ERSS	DRSS	DERSS
5	0.50	0.41	0.4	0.42	0.39
	0.75	0.31	0.31	0.31	0.3
	0.90	0.21	0.2	0.2	0.2
7	0.50	0.34	0.33	0.33	0.33
	0.75	0.26	0.25	0.25	0.25
	0.90	0.17	0.17	0.17	0.17
9	0.50	0.29	0.29	0.29	0.28
	0.75	0.23	0.22	0.22	0.22
	0.90	0.15	0.15	0.15	0.15

naturally, whereas $\delta > 0$ means that a special cause of variation is interrupting the process and switching it from an in-control state to an out-of-control one. The shift that can occur in the variable of interest while dealing with the auxiliary information-based location control charts is illustrated by Ahmad et al. (2014), Riaz (2008) and Riaz et al. (2014). In more detail, for the computation of power, we assume the process follows bivariate distribution D(such as BN, BT and BLN) with known in-control parameters $\mu_Y = 0$, $\mu_X = 0$, $\sigma_Y^2 = 1$, $\sigma_X^2 = 1$ and $v = 30$ (one may continue for other choices of parameters). Thus, for a given value of design parameters G, D, $k-m$, k, n and ρ_{YX} at $\alpha = 0.0027$, pick the required control limits factors given in Tables 1 and 2, and construct the control limits (given in Equation 10). In

Downloaded from tim.sagepub.com at UNIV CALIFORNIA SAN DIEGO on March 15, 2016

Table 4. Skewness correction factor c_4^* of $V_{(G,SC)}$ charts for varying values of n, ρ_{YX}, k_3 and G.

n	ρ_{YX}	k_3	G			
			RSS	ERSS	DRSS	DERSS
5	0.50	0.8	0.18	0.18	0.17	0.16
		2	0.46	0.44	0.42	0.35
		2.4	0.59	0.48	0.52	0.4
	0.75	0.8	0.11	0.12	0.09	0.1
		2	0.31	0.25	0.26	0.21
		2.4	0.32	0.27	0.3	0.24
	0.90	0.8	0.05	0.05	0.05	0.04
		2	0.08	0.1	0.11	0.09
		2.4	0.16	0.15	0.15	0.11
	0.50	0.8	0.13	0.1	0.13	0.11
		2	0.33	0.28	0.31	0.24
		2.4	0.38	0.31	0.34	0.29
7	0.75	0.8	0.07	0.06	0.07	0.06
		2	0.18	0.17	0.18	0.15
		2.4	0.23	0.18	0.23	0.16
	0.90	0.8	0.03	0.03	0.03	0.02
		2	0.08	0.06	0.08	0.07
		2.4	0.11	0.08	0.1	0.08
9	0.50	0.8	0.02	0.09	0.08	0.08
		2	0.22	0.19	0.22	0.18
		2.4	0.28	0.23	0.27	0.27
	0.75	0.8	0.06	0.05	0.06	0.05
		2	0.15	0.12	0.12	0.12
		2.4	0.16	0.13	0.15	0.14
	0.90	0.8	0.02	0.03	0.02	0.02
		2	0.06	0.05	0.06	0.05
		2.4	0.08	0.06	0.08	0.06

the next step, for a given value of design parameters and δ, generate the random samples from bivariate distribution D (such as BN, BT and BLN) and check whether the value of test statistic is inside or outside the control limits ($LCL_{V_{(G,D)}}$, $UCL_{V_{(G,D)}}$). The following procedure is repeated 10^5 time for varying values of the design parameters with different amount of δ and finally, proportions of the test statistic beyond the control limits ($LCL_{V_{(G,D)}}$, $UCL_{V_{(G,D)}}$) are required for the probability of signals (power) and are displayed in Figures 1–4. We also computed ARL_1 and displayed it in Figures 5 and 6. It is important to mention here that for a fixed false alarm rate, we expect a larger value of probability of signals and a smaller value of ARL_1.

Performance evaluation of $V_{(G,SC)}$ control charts

To evaluate the performance of $V_{(G,SC)}$ control charts in term of the false alarm rate, we consider the bivariate skewed distribution (bivariate gamma), bivariate heavy tailed distribution (BLN) and different contaminated situations. For a contaminated environment, we consider localized variance disturbance (LVD) and localized mean disturbance (LMD). The details of LVD and LMD can be seen in the articles of Tatum (1997) and Shoonhoven et al. (2011). Moreover, in the case of LVD, 95% probability of each sample being drawn from a BLN distribution (represents a heavy tailed distribution) with scale matrix is $\Sigma = \begin{bmatrix} \sigma^2_{Y(s)} & \sigma_{Y(s)}\sigma_{X(s)}\rho_{YX} \\ \sigma_{Y(s)}\sigma_{X(s)}\rho_{YX} & \sigma^2_{X(s)} \end{bmatrix}$ and 5% probability of being drawn from $\Sigma = \begin{bmatrix} a^2\sigma^2_{Y(s)} & a\sigma_{Y(s)}\sigma_{X(s)}\rho_{YX} \\ a\sigma_{Y(s)}\sigma_{X(s)}\rho_{YX} & \sigma^2_{X(s)} \end{bmatrix}$, where $a > 1$ is the amount of disturbance in the variance of study variable, and $\sigma^2_{Y(s)}$ and $\sigma^2_{X(s)}$ are variances of the scale matrix. Likewise, in the case of LVD, 95% probability of each sample being drawn from a given BLN distribution with the given location vector is $\mu = ln\begin{bmatrix} 1 \\ 1 \end{bmatrix}$ and 5% probability of being drawn from $\mu = ln\begin{bmatrix} b \\ 1 \end{bmatrix}$, respectively, where $b > 1$ is the amount of disturbances in the mean of study variable. The remaining steps for computing the false alarm rate is as follows:

Step 1: For a given value of design parameters G, k_3, n and ρ_{YX} at $\alpha = 0.0027$, pick the required control limits factors given in Table 5, and construct the control limits (given in Equation 12).

Step 2: random samples of size n are generated from skewed distribution, heavy tailed distribution and their contaminated environments for a given value of G, k_3, n, ρ_{YX}, a and b.

Downloaded from tim.sagepub.com at UNIV CALIFORNIA SAN DIEGO on March 15, 2016

Table 5. Control limits coefficients (B_L^*, B_U^*) of $\mathbf{V}_{(G,SC)}$ charts at varying values of n, ρ_{YX}, k_3 and G.

n	ρ_{YX}	k_3	G							
			RSS		ERSS		DRSS		DERSS	
			B_L^*	B_U^*	B_L^*	B_U^*	B_L^*	B_U^*	B_L^*	B_U^*
5	0.50	0.8	1.04	1.40	1.02	1.37	1.1	1.44	1	1.33
		2	0.76	1.68	0.75	1.64	0.85	1.68	0.81	1.51
		2.4	0.63	1.81	0.71	1.68	0.74	1.79	0.76	1.56
	0.75	0.8	0.83	1.04	0.82	1.05	0.83	1.02	0.78	0.99
		2	0.63	1.25	0.69	1.19	0.67	1.18	0.68	1.09
		2.4	0.61	1.26	0.67	1.21	0.62	1.22	0.65	1.13
	0.90	0.8	0.57	0.68	0.56	0.66	0.56	0.66	0.56	0.10
		2	0.54	0.7	0.51	0.71	0.49	0.72	0.51	0.15
		2.4	0.46	0.78	0.47	0.76	0.46	0.75	0.46	0.17
7	0.5	0.8	0.89	1.14	0.89	1.09	0.87	1.13	0.87	1.09
		2	0.69	1.35	0.71	1.28	0.69	1.31	0.74	1.22
		2.4	0.63	1.4	0.68	1.31	0.65	1.34	0.69	1.27
	0.75	0.8	0.71	0.85	0.69	0.82	0.69	0.83	0.68	0.81
		2	0.6	0.96	0.59	0.92	0.58	0.95	0.6	0.89
		2.4	0.55	1.01	0.58	0.93	0.54	0.99	0.59	0.9
	0.90	0.8	0.49	0.54	0.48	0.53	0.47	0.54	0.47	0.52
		2	0.43	0.59	0.44	0.57	0.43	0.58	0.43	0.57
		2.4	0.4	0.62	0.42	0.59	0.41	0.6	0.42	0.58
9	0.50	0.8	0.86	0.91	0.78	0.95	0.78	0.95	0.76	0.93
		2	0.66	1.1	0.67	1.06	0.64	1.08	0.67	1.02
		2.4	0.6	1.17	0.63	1.1	0.59	1.13	0.57	1.12
	0.75	0.8	0.62	0.74	0.62	0.73	0.6	0.71	0.62	0.71
		2	0.53	0.83	0.55	0.79	0.53	0.78	0.55	0.78
		2.4	0.52	0.85	0.54	0.8	0.51	0.8	0.53	0.8
	0.90	0.8	0.42	0.47	0.42	0.47	0.42	0.47	0.42	0.46
		2	0.38	0.5	0.39	0.5	0.39	0.5	0.4	0.49
		2.4	0.36	0.52	0.39	0.5	0.37	0.52	0.39	0.5

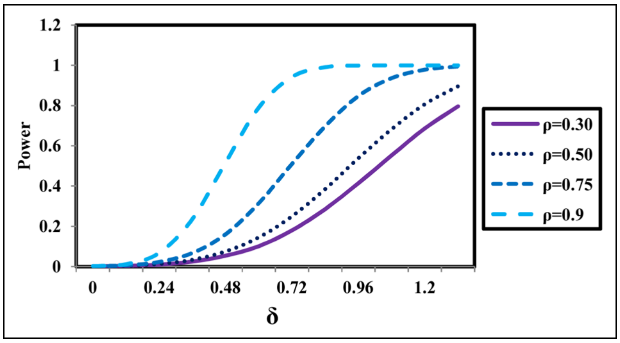

Figure 1. Power curves of $V_{(RSS,BN)}$ control charts with an increase in ρ_{YX} at $\alpha = 0.0027$.

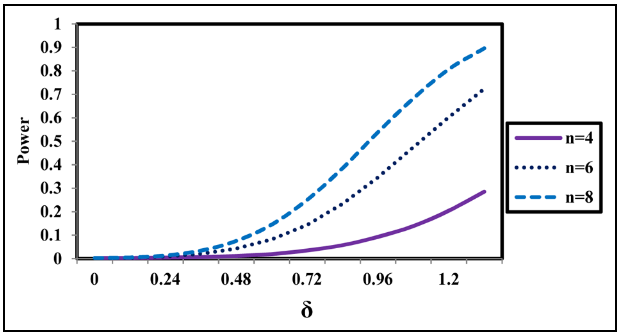

Figure 2. Power curves of $V_{(RSS,BN)}$ control charts with different sample sizes n at $\alpha = 0.0027$.

Step 3: The proportion of test statistics ($V_{G,j}$, $j = 1, 2, 3, \ldots r$) going outside the control limits ($LCL_{V_{(G,D)}}$,$UCL_{V_{(G,D)}}$) is considered the false alarm rate.
We have provided results in Tables 6–8 for some selective choices of k_3, n, ρ_{YX}, a and b at $\alpha = 0.0027$.

Results and discussion

We have observed the following results based on the Figures 1–6 and Tables 6–8.

Performances analysis of the proposed charts

- Performance of $V_{(G,D)}$ control charts is increasing with an increase in $k - m, k, n, \rho_{YX}$ and δ (Figures 1–6).
- Attaching more runs rules with the design structures of $V_{(G,D)}$ control charts are helpful for detection of smaller and moderate shifts (Figure 3).
- Among different sampling strategies, double ranked set strategies-based $V_{(G,D)}$ control charts have the best performance compared with single ranked set

Downloaded from tim.sagepub.com at UNIV CALIFORNIA SAN DIEGO on March 15, 2016

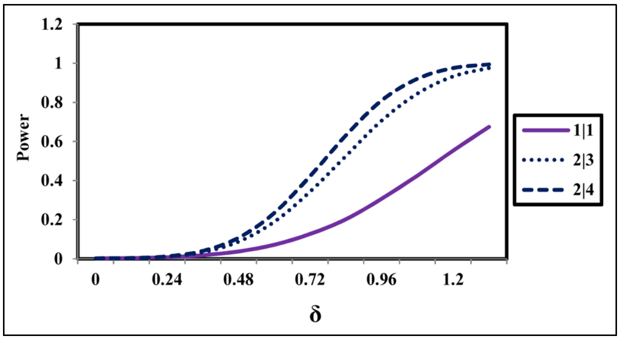

Figure 3. Power curves of $V_{(ERSS,BN)}$ control charts with different choices of $k-m$ and k at $\alpha = 0.0027$.

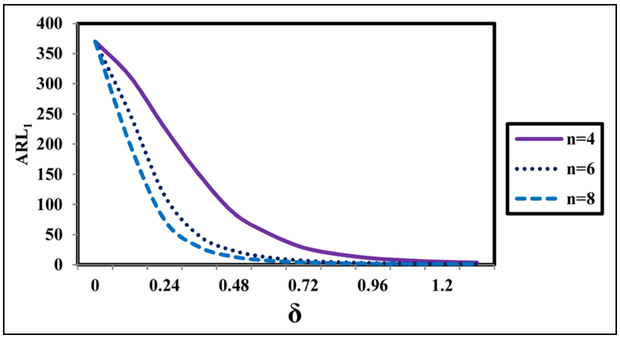

Figure 6. ARL_1 curves of $V_{(RSS,BN)}$ control charts with different sample sizes n at $\alpha = 0.0027$.

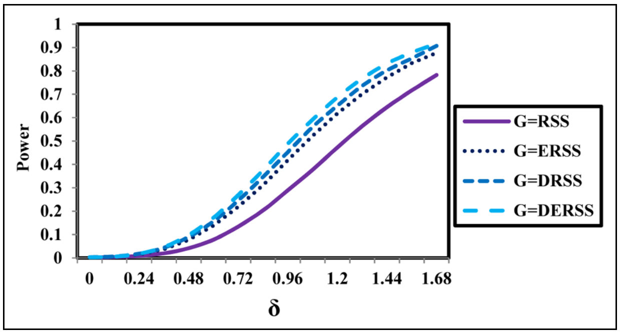

Figure 4. Power curves of $V_{(G,BLN)}$ control charts for different choices G at $\alpha = 0.0027$.

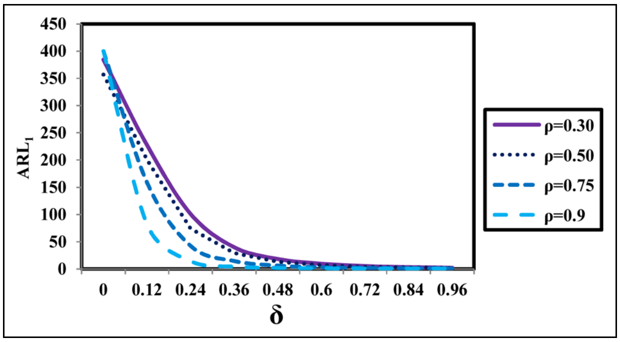

Figure 5. ARL_1 curves of $V_{(RSS,BN)}$ control charts with an increase in ρ_{YX} at $\alpha = 0.0027$

strategies-based control charts in general (Figure 4). Moreover, among single ranked set strategies, the ERSS-based control chart is first followed by RSS, whereas in double ranked set strategies, DERSS comes first followed by DRSS.

- Performance of $V_{(G,D)}$ control charts with varying sampling strategies and runs rules comes closer to each other for large sample size n, but in most of the practices we have a limited sample size (such as $n = 4$ and $n = 6$).

Comparative analysis of proposed control charts

- When the design structure of $V_{(G,D)}$ control charts are based on a known symmetrical distribution (such as BN), but an on-going process follows a skewed, heavy tailed (such as BLN and bivariate gamma) distribution or contaminated skewed distribution, then in such a situation extreme ranked set strategies-based control charts are comparatively more robust than ranked set strategies-based control charts. It is important to mention here that for skewed distribution with a large sample size and high skewness level, the false alarm rate of double extreme ranked set strategies-based control charts is noticeably smaller than other ranked set strategies-based control charts (Table 6).
- Skewness correction control charts $V_{(G,SC)}$ are more robust than known symmetrical distribution-based control charts, when process distributions are skewed and heavy tailed (with small sample size $n = 5$). Among different skewness correction control charts, extreme ranked set strategies-based control charts are ranked first due to their ability of maintaining the false alarm rate followed by ranked set strategies-based control charts, especially for high skewness ($k_3 \geq 2$), small sample size ($n = 5$) and $\rho_{YX} > 0.5$. Furthermore, for large sample size ($n \geq 7$), ranked set strategies-based $V_{(G,SC)}$ control charts were ranked first, followed by extreme ranked set strategies-based control charts.
- When the sample size is large ($n > 7$), the $V_{(\text{DERSS,BN})}$ control chart is relatively more robust than the $V_{(G,SC)}$ control charts (Table 6). However, it is a fact that in most of the practices the practitioner prefers a small sample with high precision.

Special cases

- The design structures (Equations 11 and 12) of the $V_{(G,SC)}$ control charts merge with other existing design structures such as those of Chan and Cui (2003) and Riaz et al. (2015a, 2015b) when $\rho_{YX} = 0$.

Downloaded from tim.sagepub.com at UNIV CALIFORNIA SAN DIEGO on March 15, 2016

Table 6. False alarm rate of $V_{(G,SC)}$ and $V_{(G,BN)}$ control charts under skewed and heavy tailed distribution with varying values of $\rho_X n$, k_3 and G at $\alpha = 0.0027$.

$k_3 = 0.8$

ρ_{YX}	Distributions	Methods	RSS		ERSS		DRSS		DERSS	
			n=5	*n*=7	*n*=5	*n*=7	*n*=5	*n*=7	*n*=5	*n*=7
0.50	Lognormal	$V_{(G,SC)}$	0.0041	0.0026	0.0032	0.0031	0.0038	0.0042	0.0033	0.0040
		$V_{(G,BN)}$	0.0050	0.0028	0.0044	0.0034	0.0048	0.0042	0.0036	0.0026
	Gamma	$V_{(G,SC)}$	0.0034	0.0028	0.0035	0.0028	0.0030	0.0038	0.0022	0.0022
		$V_{(G,BN)}$	0.0042	0.0032	0.0045	0.0038	0.0030	0.0038	0.0032	0.0032
0.75	Lognormal	$V_{(G,SC)}$	0.0034	0.0026	0.0028	0.0028	0.0027	0.0044	0.0041	0.0050
		$V_{(G,BN)}$	0.0040	0.0040	0.0034	0.0026	0.0036	0.0036	0.0026	0.0026
	Gamma	$V_{(G,SC)}$	0.0041	0.0031	0.0025	0.0028	0.0035	0.0028	0.0028	0.0018
		$V_{(G,BN)}$	0.0042	0.0031	0.0030	0.0034	0.0035	0.0031	0.0028	0.0019
0.90	Lognormal	$V_{(G,SC)}$	0.0042	0.0027	0.0032	0.0018	0.0030	0.0022	0.0032	0.0050
		$V_{(G,BN)}$	0.0046	0.0036	0.0035	0.0026	0.0030	0.0031	0.0021	0.0031
	Gamma	$V_{(G,SC)}$	0.0035	0.0022	0.0026	0.0021	0.0036	0.0030	0.0025	0.0014
		$V_{(G,BN)}$	0.0036	0.0033	0.0026	0.0021	0.0042	0.0036	0.0024	0.0017

$k_3 = 2$

ρ_{YX}	Distributions	Methods	RSS		ERSS		DRSS		DERSS	
			n=5	*n*=7	*n*=5	*n*=7	*n*=5	*n*=7	*n*=5	*n*=7
0.50	Lognormal	$V_{(G,SC)}$	0.0070	0.0054	0.0050	0.0052	0.0046	0.0040	0.0056	0.0100
		$V_{(G,BN)}$	0.0102	0.0082	0.0089	0.0062	0.0098	0.0067	0.0062	0.0040
	Gamma	$V_{(G,SC)}$	0.0062	0.0038	0.0037	0.0024	0.0032	0.0036	0.0017	0.0019
		$V_{(G,BN)}$	0.0094	0.0068	0.0084	0.0055	0.0080	0.0050	0.0064	0.0048
0.75	Lognormal	$V_{(G,SC)}$	0.0056	0.0047	0.0052	0.0038	0.0054	0.0040	0.0045	0.0078
		$V_{(G,BN)}$	0.0086	0.0076	0.0072	0.0049	0.0078	0.0067	0.0054	0.0040
	Gamma	$V_{(G,SC)}$	0.0062	0.0051	0.0050	0.0014	0.0041	0.0036	0.0016	0.0010
		$V_{(G,BN)}$	0.0072	0.0064	0.0063	0.0033	0.0058	0.0050	0.0034	0.0026
0.90	Lognormal	$V_{(G,SC)}$	0.0132	0.0052	0.0042	0.0037	0.0040	0.0026	0.0036	0.0040
		$V_{(G,BN)}$	0.0065	0.0061	0.0059	0.0048	0.0064	0.0050	0.0039	0.0026
	Gamma	$V_{(G,SC)}$	0.0163	0.0050	0.0039	0.0016	0.0044	0.0025	0.0010	0.0004
		$V_{(G,BN)}$	0.0062	0.0050	0.0040	0.0018	0.0045	0.0039	0.0018	0.0010

$k_3 = 2.4$

ρ_{YX}	Distributions	Methods	RSS		ERSS		DRSS		DERSS	
		n=5	*n*=7	*n*=5	*n*=7	*n*=5	*n*=7	*n*=5	*n*=7	
0.50	Lognormal	$V_{(G,SC)}$	0.0096	0.0057	0.0079	0.0056	0.0070	0.0046	0.0094	0.0168
		$V_{(G,BN)}$	0.0124	0.011	0.011	0.0061	0.0115	0.0104	0.0084	0.0065
	Gamma	$V_{(G,SC)}$	0.0104	0.0046	0.0056	0.0025	0.0052	0.0032	0.0021	0.0024
		$V_{(G,BN)}$	0.0121	0.0099	0.0106	0.0060	0.0120	0.0086	0.0078	0.0064
0.75	Lognormal	$V_{(G,SC)}$	0.0071	0.0044	0.0056	0.0048	0.0054	0.0041	0.0050	0.0082
		$V_{(G,BN)}$	0.0102	0.0094	0.0080	0.0050	0.0099	0.0081	0.0063	0.0036
	Gamma	$V_{(G,SC)}$	0.0134	0.0058	0.0054	0.0014	0.0066	0.0034	0.0015	0.0006
		$V_{(G,BN)}$	0.0120	0.0064	0.0067	0.0030	0.0070	0.0060	0.0046	0.0024
0.90	Lognormal	$V_{(G,SC)}$	0.0045	0.0044	0.0053	0.0040	0.0038	0.0039	0.0034	0.0038
		$V_{(G,BN)}$	0.0075	0.0052	0.0068	0.0044	0.0060	0.0057	0.0050	0.0036
	Gamma	$V_{(G,SC)}$	0.0107	0.0050	0.0046	0.0013	0.0065	0.0032	0.0010	0.0003
		$V_{(G,BN)}$	0.0088	0.0048	0.0050	0.0026	0.0066	0.0044	0.0018	0.0007

- The efficient design structures given in Equation (10) can be considered the generalized form of other existing studies such as Abbasi and Riaz (2015) when $G = (RSS \text{ and } ERSS), D = BN, k - m = 1, k = 1$ (Figures 1–3).

A real application

In this section, we provide a real-life application of the proposed control charts for monitoring the stability of physicochemical parameters of groundwater. The stability of ground

Downloaded from tim.sagepub.com at UNIV CALIFORNIA SAN DIEGO on March 15, 2016

Table 7. False alarm rate of $\boldsymbol{V}_{(G,SC)}$ and $\boldsymbol{V}_{(G,BN)}$ control charts under contaminated (localized variance disturbances) heavy tailed distribution (bivariate lognormal) with varying values of $\boldsymbol{\rho_{YX}n}$, $\boldsymbol{k_3}$ and $\boldsymbol{G}$ at $\boldsymbol{\alpha} = 0.0027$.

$\boldsymbol{\rho_{YX}}$	$\boldsymbol{n}$	Methods	RSS	ERSS	DRSS	DERSS
$\boldsymbol{k_3} = 2$ and $a = 1.50$						
0.5	5	$\boldsymbol{V_{(G,SC)}}$	0.0080	0.0100	0.0121	0.0097
		$\boldsymbol{V_{(G,BN)}}$	0.0170	0.0150	0.0167	0.0130
	7	$\boldsymbol{V_{(G,SC)}}$	0.0088	0.0107	0.0089	0.0153
		$\boldsymbol{V_{(G,BN)}}$	0.0153	0.0125	0.0152	0.0100
0.75	5	$\boldsymbol{V_{(G,SC)}}$	0.0113	0.0104	0.0084	0.0114
		$\boldsymbol{V_{(G,BN)}}$	0.0157	0.0153	0.0136	0.0162
	7	$\boldsymbol{V_{(G,SC)}}$	0.0100	0.0114	0.0093	0.0156
		$\boldsymbol{V_{(G,BN)}}$	0.0141	0.0143	0.0150	0.0157
$\boldsymbol{k_3} = 2.4$ and $a = 1.20$						
0.5	5	$\boldsymbol{V_{(G,SC)}}$	0.0113	0.0112	0.0070	0.0067
		$\boldsymbol{V_{(G,BN)}}$	0.0119	0.0115	0.0136	0.0093
	7	$\boldsymbol{V_{(G,SC)}}$	0.0080	0.0062	0.0060	0.0158
		$\boldsymbol{V_{(G,BN)}}$	0.0130	0.0094	0.0112	0.0059
0.75	5	$\boldsymbol{V_{(G,SC)}}$	0.0076	0.0060	0.0054	0.0050
		$\boldsymbol{V_{(G,BN)}}$	0.0113	0.0097	0.0098	0.0072
	7	$\boldsymbol{V_{(G,SC)}}$	0.0068	0.0057	0.0042	0.0100
		$\boldsymbol{V_{(G,BN)}}$	0.0098	0.0079	0.0085	0.0070

Table 8. False alarm rate of $V_{(G,SC)}$ and $V_{(G,BN)}$ control charts under contaminated (localized mean disturbances) heavy tailed distribution (bivariate lognormal) with varying values of $\rho_{YX}n, k_3$ and G at $\alpha = 0.0027$.

$\boldsymbol{\rho_{YX}}$	$\boldsymbol{n}$	Methods	RSS	ERSS	DRSS	DERSS
$k_3 = 2$ and $b = 1.50$						
0.5	5	$\boldsymbol{V_{(G,S)}}$	0.0071	0.0080	0.0054	0.0077
		$\boldsymbol{V_{(G,BN)}}$	0.0122	0.0118	0.0120	0.0096
	7	$\boldsymbol{V_{(G,SC)}}$	0.0048	0.0053	0.0051	0.0110
		$\boldsymbol{V_{(G,BN)}}$	0.0099	0.0084	0.0116	0.0062
0.75	5	$\boldsymbol{V_{(G,SC)}}$	0.0059	0.0056	0.0050	0.0055
		$\boldsymbol{V_{(G,BN)}}$	0.0112	0.0099	0.0110	0.0081
	7	$\boldsymbol{V_{(G,SC)}}$	0.0048	0.0058	0.0052	0.0072
		$\boldsymbol{V_{(G,BN)}}$	0.0107	0.0083	0.0096	0.0063
$\boldsymbol{k_3} = 2.4$ and $b = 1.20$						
0.5	5	$\boldsymbol{V_{(G,SC)}}$	0.0090	0.0077	0.0069	0.0062
		$\boldsymbol{V_{(G,BN)}}$	0.0118	0.0106	0.0131	0.0088
	7	$\boldsymbol{V_{(G,SC)}}$	0.0074	0.0060	0.0055	0.0157
		$\boldsymbol{V_{(G,BN)}}$	0.0132	0.0092	0.0133	0.0063
0.75	5	$\boldsymbol{V_{(G,SC)}}$	0.0085	0.0063	0.0060	0.0053
		$\boldsymbol{V_{(G,BN)}}$	0.0137	0.0120	0.0110	0.0085
	7	$\boldsymbol{V_{(G,SC)}}$	0.0046	0.0059	0.0050	0.0089
		$\boldsymbol{V_{(G,BN)}}$	0.0105	0.0098	0.0116	0.0066

water parameters is always considered important for industrial processes, crop yields and for drinking water, which all ultimately affect industrial production, production of a crop and human health, respectively (cf. Sundaram et al., 2009). More specifically, crop yield depends on a number of factors such as colour, acidity, hardness, PH, temperature and sulphite. In order to show the application of the proposed control charts, we consider two physico-chemical parameters of groundwater, which include total dissolved solids and total hardness of water. In more detail, total dissolved solids is considered a study variable Y and measured in terms of electric conductivity (EC), whereas the total hardness of water is considered an auxiliary variable X and measured in terms of calcium magnesium carbonates.

Downloaded from tim.sagepub.com at UNIV CALIFORNIA SAN DIEGO on March 15, 2016

In order to show the significance of the proposed location control charts, we consider groundwater (used for irrigation of crops) of District Rahim Yar Khan, Pakistan. In more detail, we randomly selected 30 locations and from each location a sample of size 5 is gathered under ERSS [the procedure of ERSS can be seen above, and further details may also be seen in Mehmood et al. (2013), Abbasi and Riaz (2015) and Riaz et al. (2015a, 2015b)]. The actual measurements of electric conductivity and calcium magnesium carbonate are tabulated in Table 9. We also draw a probability density plot of the electric conductivity and calcium magnesium carbonate after standardizing the variables (Figure 7). Before implementing the control charts, we calculated the statistic ($V_{ERSS,j}, j = 1, 2, 3, \ldots r$) using the data set given in Table 9 with known in-control parameters: $\mu_Y = 836.06$, $\mu_X = 4.93, \sigma_Y^2 = 1000$, $\sigma_X^2 = 1.53$, and $\rho_{YX} = 0.50$.

After calculating the charting statistic, we have implemented the $V_{(ERSS,BN)}$ control chart with two runs rules (1|1 and 2|3) for monitoring the variation of each water sample of a given location with respect to the electric conductivity (Figure 8).

From Figure 8, it is clear that no signal is detected by the first point decision rule ($(k-m)|k = 1|1$), whereas two signals are triggered by $(k-m)|k = 2|3$. This shows that attaching more runs rules with the design structure of the usual auxiliary information based on control charts boosts the performance of the control chart. The ensuing outcomes are in accordance with results and discussion (see earlier in the paper).

Concluding remarks

In the current article, we considered regression estimators under different ranked set strategies. These estimators utilized the auxiliary information at both stages instead of using it either for ranking the units or estimation. Based on these estimators we proposed location control charts for known process distributions and unknown skewed process distributions. We have investigated the performance of proposed control charts using the probability of signals, false alarm rate and average run length by taking into accounts symmetrical, skewed and heavy tailed distributions along with their contaminated cases.

The results of the current study indicated that control charts based on extreme ranked set strategies have higher signalling probability than control charts based on ranked set strategies. Moreover, design structures of extreme ranked set strategies-based control charts under known symmetrical distribution are more robust than ranked set strategies-based control charts for skewed, heavy tailed and contaminated distributions.

Likewise we have investigated the performance of skewness correction control charts $V_{(G,SC)}$. The results revealed that $V_{(G,SC)}$ control charts are more robust for skewed and heavy tailed processes (especially for small sample size) compared with known symmetrical distribution-based on control charts. Our further analysis, among different $V_{(G,SC)}$ control charts and extreme ranked set strategies-based control charts (with high skewness) are ranked first for small sample sizes, followed by ranked set strategies-based control charts for

Table 9. Actual measurement of electric conductivity Y and calcium-magnesium carbonates X.

Location (j)		Observation (i)				
		1	2	3	4	5
1	$Y_{[i,\,erss],\,1}$	860	817	880	912	856
	$X_{(i,\,erss),\,1}$	6.4	5.8	4.4	6.5	4.8
2		846	830	845	890	897
		3.8	3.5	3.6	3.8	4.2
3		850	828	879	803	887
		7.5	6.3	6	6.2	6.9
4		806	835	780	790	757
		4.7	2.7	3	3.7	5.1
5		750	792	760	720	791
		4.2	5.8	4.6	3.7	3
6		744	720	790	775	782
		3.2	3.6	3.5	4.9	4.7
7		870	810	840	815	820
		1.9	1.8	1.7	2	3
8		888	825	860	880	895
		4.5	5.1	4.8	4.7	5
9		775	792	750	812	742
		5	4	4.9	3	3.7
10		868	885	885	900	860
		3.5	3.8	4	4.5	3.2
11		825	810	825	850	830
		2.5	1.8	1.7	1.8	2
12		792	811	816	845	870
		5.8	5.9	4.7	5.3	6
13		933	870	909	933	925
		6	6.5	6	6.3	6.6
14		910	909	888	933	860
		5.3	7.2	7.3	6.3	6.2
15		950	960	927	990	890
		7	6.3	6.6	7.3	5.1
16		967	960	914	895	935
		5	6	4.5	6.5	6.4
17		920	909	867	890	945
		6.4	6.2	5.2	5.9	7.2
18		850	828	879	803	887
		7.5	6.3	6	6.2	6.9
19		760	725	790	795	750
		5.7	5.5	5.9	5.8	5.6
20		781	740	798	803	812
		4	3.6	5	5	5.2
21		870	845	835	828	773
		6.8	6.3	6.4	6.2	6
22		858	873	880	900	820
		3.1	3.3	3	3.5	3.7
23		745	773	730	780	732
		3.8	4.8	3.6	3.9	4.3
24		990	933	940	980	933
		.	6	4.7	4	6.3
25		872	907	914	830	856
		7	6.7	7.3	6.4	6
26		830	780	867	820	825
		5.7	5.5	5.9	5.7	5.6
27		750	790	860	860	810
		5.8	4.8	5.5	5.3	5
28		880	840	867	909	867
		5	4.3	4.9	5.2	4.8
29		880	918	915	890	840

(continued)

Downloaded from tim.sagepub.com at UNIV CALIFORNIA SAN DIEGO on March 15, 2016

Table 9. Continued

Location (j)	Observation (i)				
	1	2	3	4	5
	6.3	6.8	6.6	6.8	6
30	747	730	730	720	790
	2.9	3.6	2.2	3.6	3.2

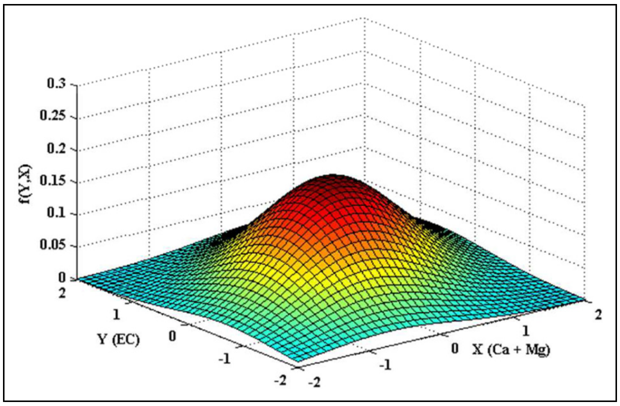

Figure 7. Probability density plot of the standardized bivariate variables (electric conductivity and calcium magnesium carbonates).

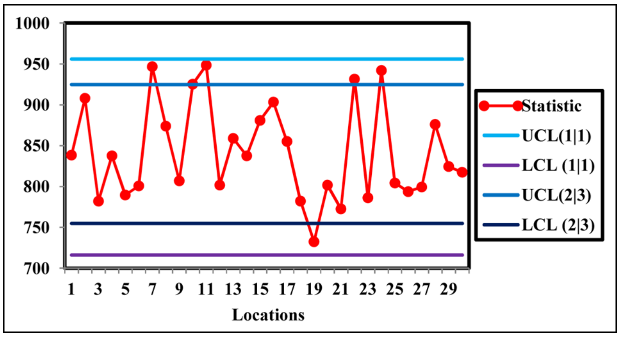

Figure 8. Monitoring the location parameter of electric conductivity through control charts.

larger sample sizes. Lastly, the real-life example shows favourable results.

Acknowledgements

The authors are indebted to King Fahd University of Petroleum and Minerals (KFUPM), Dhahran, Saudi Arabia, for providing excellent research facilities and a dynamic research environment.

Conflict of Interest Statement

The author(s) declared no potential conflicts of interest with respect to the research, authorship, and/or publication of this article.

Funding

The author(s) disclosed receipt of the following financial support for the research, authorship, and/or publication of this article: This work is sponsored by Deanship of Scientific Research KFUPM under project number FT131017.

References

Abbasi SA and Riaz M (2015) On dual use of auxiliary information for efficient monitoring. *Quality and Reliability Engineering International*, doi: 10.1002/qre.1785.

Abujiya MR and Muttlak HA (2004) Quality control chart for the mean using doublerank set sampling. *Journal of Applied Statistics* 31: 1185–1201.

Abujiya MR, Riaz M and Lee MH (2015) Enhanced cumulative sum charts for monitoring process dispersion. *PLoS ONE* 10(4): e0124520. doi:10.1371/journal.pone.0124520.

Ahmad S, Abbasi SA, Riaz M, et al. (2014) On efficient use of auxiliary information for control charting in SPC. *Computers & Industrial Engineering* 67: 173–184.

Ahmad L, Aslam M and Jun CH (2013) Designing of X-bar control charts based on process capability index using repetitive sampling. *Transactions of the Institute of Measurement and Control*, doi: 0142331213502070.

Chan LK and Cui HJ (2003) Skewness correction $\bar{X}$ and R charts for skewed distributions. *Naval Research Logistics* 50(6): 555–573.

Mehmood R, Riaz M and Does RJMM (2013) Control charts for location based on different sampling schemes. *Journal of Applied Statistics* 40(3): 483–494.

Mehmood R, Riaz M and Does RJMM (2014) On the application of different ranked set sampling schemes. *Quality Engineering* 26(3): 370–378.

Montgomery DC (2009) *Introduction to Statistical Quality Control*. New York: John Wiley & Sons.

Muttlak HA (2001) Regression estimator in extreme and median ranked set samples. *Journal of Applied Statistics* 28(8): 1003–1017.

Muttlak HA and Al-Sabah WS (2003) Statistical quality control based on ranked set sampling. *Journal of Applied Statistics* 30: 1055–1078.

Riaz M (2008) Monitoring process mean level using auxiliary information. *Statistica Neerlandica* 62(4): 458–481.

Riaz M, Abbasi SA, Ahmad S, et al. (2014) On efficient phase II process monitoring charts. *The International Journal of Advanced Manufacturing Technology* 70: 2263–2274.

Riaz M and Ali S (2015) On process monitoring using location control charts under different loss functions. *Transactions of the Institute of Measurement and Control*, doi: 10.1177/0142331215583325.

Riaz M, Mehmood R and Does RJMM (2011) On the performance of different control charting rules. *Quality and Reliability Engineering International* 27(8): 1059–1067.

Riaz M, Mehmood R, Abbas N, et al. (2015a) On effective dual use of auxiliary information in variability control charts. *Quality and Reliability Engineering International*, doi: 10.1002/qre.1848.

Riaz M, Mehmood R, Iqbal R, et al. (2015b) On efficient skewness correction charts under contaminations and non-normality. *Quality and Reliability Engineering International*, doi: 10.1002/qre.1795.

Schoonhoven M and Does RJMM (2011) The $\bar{X}$ control chart under non-normality. *Quality and Reliability Engineering International* 26(2): 167–176.

Schoonhoven M, Nazir HZ, Riaz M, et al. (2011) Robust location estimators for the $\bar{X}$ control chart. *Journal of Quality Technology* 43(4): 363–379.

Sindhu TN, Riaz M, Aslam M, et al. (2015) Bayes estimation of mixture Gumbel models with industrial applications. *Transactions of*

Downloaded from tim.sagepub.com at UNIV CALIFORNIA SAN DIEGO on March 15, 2016

the Institute of Measurement and Control, doi: 10.1177/0142331215578690.

Stedinger JR, Vogel RM and Foufoula-Georgiou E (1993) Frequency analysis of extreme events. In: Maidment DR (ed.) *Handbook of Hydrology*. New York: McGraw-Hill.

Sundaram B, Feitz AJ, Caritat P de, et al. (2009) Groundwater sampling and analysis – a field guide. *Geoscience Australia*, Record, 2009/27.

Tatum LG (1997) Robust estimation of the process standard deviation for control charts. *Technometrics* 39(2): 127–141.

Yerel S and Konuk A (2009) Bivariate lognormal distribution model of cutoff grade impurities: a case study of magnesite ore deposits. *Scientific Research and Essay* 4(12): 1500.

Yu PLH and Lam K (1997) Regression estimator in ranked set sampling. *Biometrics* 53: 1070–1080.

Appendix

Overview of bivariate distribution

Bivariate normal distribution. The probability density function (pdf) of BN distribution and the probability density plot are

$$f(Y,X) = \frac{1}{2\pi\sigma_Y\sigma_X\sqrt{1-\rho_{YX}^2}} e^{\left\{\frac{-1}{2\left(1-\rho_{YX}^2\right)}\left[\left(\frac{Y-\mu_Y}{\sigma_Y}\right)^2+\left(\frac{X-\mu_X}{\sigma_X}\right)^2-2\rho_{YX}\left(\frac{Y-\mu_Y}{\sigma_Y}\right)\left(\frac{X-\mu_X}{\sigma_X}\right)\right]\right\}},$$

$$-\infty<Y<\infty,\ -\infty<X<\infty, \sigma_Y>0, \sigma_X>0,\ -1<\rho_{YX}<+1.$$

where μ_Y and μ_X are population means of Y and X, respectively, σ_Y^2 and σ_X^2 are population variances of Y and X, respectively, ρ_{YX} denotes a population regression coefficient between Y and X and expressed as: $\rho_{YX} = \frac{\sigma_{YX}}{\sigma_Y\sigma_X}$, and σ_{YX} represents population covariance between Y and X (Figure 9).

Bivariate t distribution

The pdf of the location scale BT distribution and probability density plot are

$$f(Y,X) = \frac{1}{2\pi\sigma_Y\sigma_X\sqrt{1-\rho_{YX}^2}} \left[1+\frac{\left(\frac{Y-\mu_Y}{\sigma_Y}\right)^2+\left(\frac{X-\mu_X}{\sigma_X}\right)^2-2\rho\left(\frac{Y-\mu_Y}{\sigma_Y}\right)\left(\frac{X-\mu_X}{\sigma_X}\right)}{v(1-\rho_{YX}^2)}\right]^{-\frac{(v+2)}{2}}$$

$$-\infty<Y<\infty,\ -\infty<X<\infty, \sigma_Y>0, \sigma_X>0,\ -1<\rho_{YX}<+1.$$

where μ_Y and μ_X are population means of Y and X, respectively, σ_Y^2 and σ_X^2 are population variances of Y and X, respectively, ρ_{YX} denotes a population regression coefficient between Y and X (Figure 10).

Bivariate lognormal distribution

Two positive random variables Q and W with means and variances μ_Q, $\mu_W\sigma_Q^2$ and σ_W^2 respectively are said to be BLN distributed if $Y = lnQ$ and $X = lnW$ follow BN distribution with means and variances are μ_Y, $\mu_X\sigma_Y^2$ and σ_X^2.

The pdf of BLN distribution and probability density plot are

$$f(Q,W) = \frac{1}{2\pi\sigma_Y\sigma_X\sqrt{1-\rho_{YX}^2}} e^{\left\{\frac{-1}{2\left(1-\rho_{YX}^2\right)}\left[\left(\frac{\log(Q)-\mu_Y}{\sigma_Y}\right)^2+\left(\frac{\log(W)-\mu_X}{\sigma_X}\right)^2-2\rho\left(\frac{\log(Q)-\mu_Y}{\sigma_Y}\right)\left(\frac{\log(W)-\mu_X}{\sigma_X}\right)\right]\right\}},$$

$$0<Q<\infty, 0<W<\infty, \sigma_Y>0, \sigma_X>0,\ -1<\rho_{YX}<+1.$$

where $\mu_Y = ln\left(\mu_Q\right) - \left(\frac{\sigma_Q^2}{2}\right), \mu_X = ln(\mu_W) - \left(\frac{\sigma_W^2}{2}\right), \sigma_Y^2 = ln\left(1+\frac{\sigma_Q^2}{\mu_Q^2}\right)$ and $\sigma_X^2 = ln\left(1+\frac{\sigma_W^2}{\mu_W^2}\right)$. Moreover, ρ_{YX} denotes a population regression coefficient between Y and X. For more details, one can concern Stedinger et al. (1993) and Yerel and Konuk (2009) (Figure 11).

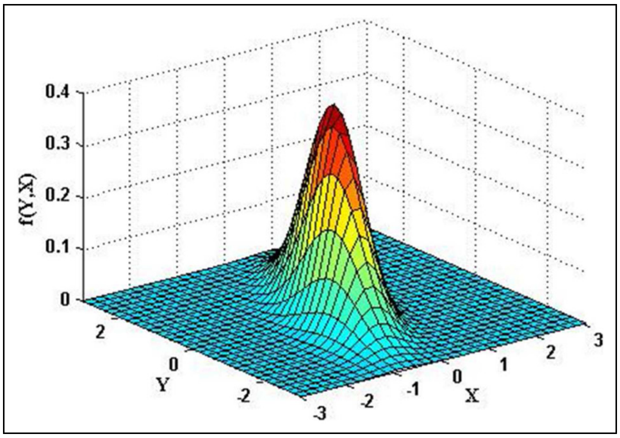

Figure 9. Probability density plot of BN distribution.

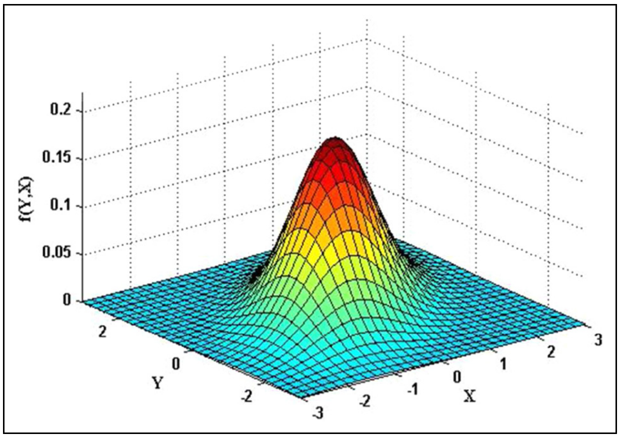

Figure 10. Probability density plot of location scale BT distribution.

Downloaded from tim.sagepub.com at UNIV CALIFORNIA SAN DIEGO on March 15, 2016

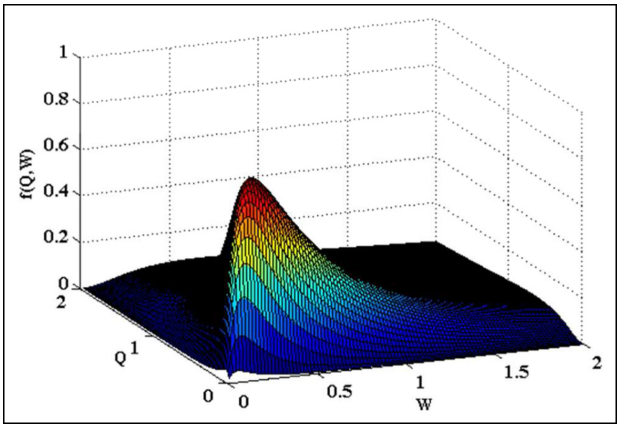

Figure 11. Probability density plot of BLN distribution.

Derivation of control limits factors using the Cornish fisher expansion

Let B be the standardized random variable with mean zero and standard deviation one, B_α and Z_α be the pth quantile of B and the standard normal distribution respectively, and k_r be the rth cumulant of X ($r \geq 3$). Then B_α has the Cornish fisher expansion (Chan and Cui, 2003) as follows:

$$B_\alpha = Z_\alpha + \frac{1}{6}\left(Z_\alpha^2 - 1\right)k_3 + \frac{1}{24}\left(Z_\alpha^3 - 3Z_\alpha\right)k_4 - \frac{1}{36}\left(2Z_\alpha^3 - 5Z_\alpha\right)k_3 + \ldots$$

In the above expression, the pth quantile is divided into $(\frac{p}{2})th$ and $(1-\frac{p}{2})th$ quantiles, and written as:

$$B_{(\frac{\alpha}{2})} = Z_{(\frac{\alpha}{2})} + \frac{1}{6}\left(Z_{(\frac{\alpha}{2})}^2 - 1\right)k_3 + \frac{1}{24}\left(Z_{(\frac{\alpha}{2})}^3 - 3Z_{(\frac{\alpha}{2})}\right)k_4 - \frac{1}{36}\left(2Z_{(\frac{\alpha}{2})}^3 - 5Z_{(\frac{\alpha}{2})}\right)k_3 + \ldots \quad (13)$$

$$B_{(1-\frac{\alpha}{2})} = Z_{(1-\frac{\alpha}{2})} + \frac{1}{6}\left(Z_{(1-\frac{\alpha}{2})}^2 - 1\right)k_3 + \frac{1}{24}\left(Z_{(1-\frac{\alpha}{2})}^3 - 3Z_{(1-\frac{\alpha}{2})}\right)k_4 - \frac{1}{36}\left(2Z_{(1-\frac{\alpha}{2})}^3 - 5Z_{(1-\frac{\alpha}{2})}\right)k_3 + \ldots \quad (14)$$

In brief, Chan and Cui (2003) considered the usual estimator ($\bar{Y} = \frac{\sum_{i=1}^{n} Y}{n}$) of the population mean under simple random sampling and proposed control limits coefficients ($B_L^* = B_{(\frac{\alpha}{2})}, B_U^* = B_{(1-\frac{\alpha}{2})}$) by using (13) and (14):

$$B_L^* = \left[Z_{(\frac{\alpha}{2})} + \frac{\frac{1}{6}\left(Z_{(\frac{\alpha}{2})}^2 - 1\right)k_3(\bar{Y})}{1 + 0.2k_3^2(\bar{Y})}\right]k_2,$$
$$B_U^* = \left[Z_{(1-\frac{\alpha}{2})} + \frac{\frac{1}{6}\left(Z_{(1-\frac{\alpha}{2})}^2 - 1\right)k_3(\bar{Y})}{1 + 0.2k_3^2(\bar{Y})}\right]k_2 \quad (15)$$

where k_2 is the ratio of the standard deviation of the sampling distribution of the statistic $\bar{Y}$ and the standard deviation of the variable of interest σ_Y, that is $k_2 = \frac{\sigma_{\bar{Y}}}{\sigma_Y} = \frac{1}{\sqrt{n}}$. Furthermore, $k_3(\bar{Y})$ represents the skewness of the statistic $\bar{Y}$, depending on the skewness of study variable (k_3) and sample size n. The control limits factors given in Equation (15) are based on usual estimator, and therefore do not have the ability to work in such circumstances when extra information about the study variable is available. Thus, we consider the regression estimators under different sampling strategies

$$V_{RSS,j} = \bar{Y}_{RSS,j} + \left(r_{YX_{RSS,j}}\frac{s_{Y_{RSS,j}}}{s_{X_{RSS,j}}}\right)\left[\mu_X - \bar{X}_{RSS,j}\right],$$

$$V_{ERSS,j} = \bar{Y}_{ERSS,j} + \left(r_{YX_{RSS,j}}\frac{s_{Y_{RSS,j}}}{s_{X_{RSS,j}}}\right)\left[\mu_X - \bar{X}_{RSS,j}\right],$$

$$V_{DRSS,j} = \bar{Y}_{DRSS,j} + \left(r_{YX_{DRSS,j}}\frac{s_{Y_{DRSS,j}}}{s_{X_{DRSS,j}}}\right)\left[\mu_X - \bar{X}_{DRSS,j}\right],$$

$$V_{DERSS,j} = \bar{Y}_{DRSS,j} + \left(r_{YX_{DRSS,j}}\frac{s_{Y_{DRSS,j}}}{s_{X_{DRSS,j}}}\right)\left[\mu_X - \bar{X}_{DRSS,j}\right]$$

and proposed the control limits factors ($B_L^* = B_{(\frac{\alpha}{2})}$, $B_U^* = B_{(1-\frac{\alpha}{2})}$) by using (13) and (14) for $V_{(G,SC)}$ control charts:

$$B_L^* = \left[Z_{(\frac{\alpha}{2})} + \frac{\frac{1}{6}\left(Z_{(\frac{\alpha}{2})}^2 - 1\right)k_3(V)}{1 + 0.2k_3^2(V)}\right]k_2, B_U^* = \left[Z_{(1-\frac{\alpha}{2})} + \frac{\frac{1}{6}\left(Z_{(1-\frac{\alpha}{2})}^2 - 1\right)k_3(V)}{1 + 0.2k_3^2(V)}\right]k_2,$$

where $k_3(V)$ refers to skewness of the statistics ($V_{G,j}$, $j = 1,2,\ldots,r$), k_2 is the ratio of standard deviation of statistics $V_{G,j}$ and standard deviation of the study variable σ_Y, i.e. $\frac{\sigma_{V_{G,j}}}{\sigma_Y} = \sqrt{\frac{1}{n}\left(1 - \rho_{YX}^2\right)\left[1 + E\left(\frac{\bar{Z}_G^2}{S_{Z_G}^2}\right)\right]}$. Moreover, $k_3(V)$ depends on the skewness of the study variable Y (whatever the level of skewness of auxiliary variable X), the amount of correlation between the study variable Y and auxiliary variable X, and sample size n. Thus B_L^* and B_U^* are dependent on the skewness of the study variable, the amount of correlation between Y and X, and sample size n instead of restricted assumptions of distribution.

Downloaded from tim.sagepub.com at UNIV CALIFORNIA SAN DIEGO on March 15, 2016
